# Supplementary material for: Determinants and Experiences of Care‐Seeking for Childhood Pneumonia in a Rural Indian Setting: A Mixed‐Methods Study
Source: Health Expect. 2025 Apr 16;28(2):e70263. doi: 10.1111/hex.70263 (PMC12002083; doi:10.1111/hex.70263)
Supplement: Supplementary file 1 — Annexure I Enrollment_checklist. [file HEX-28-e70263-s008.pdf]

Data Dictionary Codebook

Enrollment (PID: 27)

02-17-2025 15:50

**Instruments**		
**Instrument**	**Form Name**	**Events**
First Day	first_day	first_day_arm_1
Compliance Eighth Day	compliance_eighth_day	eigth_day_arm_1
Sixteenth Day	sixteenth_day	16th_day_arm_1

**Events**	
**Event Name**	**Unique event name**
First day	first_day_arm_1
Eigth day	eigth_day_arm_1
16th day	16th_day_arm_1

	#	Variable / Field Name	Field Label<br>*Field Note*	Field Attributes (Field Type, Validation, Choices, Calculations, etc.)
**Instrument: First Day (first_day)**				
	1	[record_id]	Record ID	text
	2	[date_of_form_filling_2]	Date of Form Filling:	text (datetime_dmy)
	3	[worker_code_2]	Project team member code	dropdown<br>1 201 - Pancham<br>2 202 - Krishan Dagar<br>3 401 - Durg Pal<br>4 203 - Sandeep<br>5 402 - Prem Kishor<br>6 403 - Harphool Malik<br>7 204 - Rameshwar Sahu<br>8 205 - Yashpal<br>9 206 - Sunil<br>10 207 - Anuj<br>11 301 - Kavita<br>12 302 - Deepti<br>13 303 - Aarti
	4	[child_name]	Child Name	text
	5	[id]	ID	text
	6	[child_available_2]	Is the child available?	yesno, Required<br>1 Yes<br>0 No
	7	[child_not_reason_2]<br>Show the field ONLY if:<br>[child_available_2] = '0'	If child is not available, please select reason	dropdown, Required<br>1 Admit<br>2 Shifted out of study area<br>3 Death<br>4 Other
	8	[child_admit_place_2]<br>Show the field ONLY if:<br>[child_not_reason_2] = '1'	Where is the child admitted ?	text
	9	[child_shifted_place_2]<br>Show the field ONLY if:	Where the child shifted?	text

		[child_not_reason_2] = '2'		
	10	[date_of_death_2]<br><br>Show the field ONLY if:<br>[child_not_reason_2] = '3'	Date of Death	text (date_dmy)
	11	[summary_of_death_2]<br><br>Show the field ONLY if:<br>[child_not_reason_2] = '3'	Summary of Death	notes
	12	[child_not_reason_other_2]<br><br>Show the field ONLY if:<br>[child_not_reason_2] = '4'	Please specify other	text
	13	[name_of_child_enrollment]<br><br>Show the field ONLY if:<br>[child_available_2]='1'	Name of child	text, Required
	14	[enrol_case_identified_at]	Case Identified at:	dropdown<br>1 Government Facility<br>2 Private (Formal)<br>3 Private Facility (Non-Formal)
	15	[enrol_gov_facility_type]<br><br>Show the field ONLY if:<br>[enrol_case_identified_at] ='1'	Type of Government Facility:	dropdown<br>1 GH Palwal<br>2 CHC<br>3 PHC<br>4 HWC
	16	[enrol_name_of_chc]<br><br>Show the field ONLY if:<br>[enrol_gov_facility_type] ='2'	Name of CHC	dropdown<br>1 Aurangabad<br>2 Hathin<br>3 Dudhola<br>4 Alawalpur
	17	[enrol_name_of_phc]<br><br>Show the field ONLY if:<br>[enrol_gov_facility_type] ='3'	Name of PHC	dropdown<br>1 Alika<br>2 Amarpur<br>3 Aurangabad<br>4 Deghot<br>5 Kalsada<br>6 Alawalpur
	18	[enrol_name_of_hwc]<br><br>Show the field ONLY if:<br>[enrol_gov_facility_type] ='4'	Name of HWC	dropdown<br>1 Rundhi<br>2 Seilothi<br>3 Pingore<br>4 Gudrana<br>5 Phoolwari<br>6 Durgapur<br>7 Bhanguri

				8 Maheshpur<br>9 Rampur Khor<br>10 Ghori<br>11 Out of study area
	19	[enrol_private_formal]<br><br>Show the field ONLY if:<br>[enrol_case_identified_at] ='2'	Name of Private facility (Formal)	text
	20	[enrol_private_non_formal]<br><br>Show the field ONLY if:<br>[enrol_case_identified_at] ='3'	Name of Private facility (Non-Formal)	text
	21	[specify_dr_name]<br><br>Show the field ONLY if:<br>[enrol_case_identified_at] ='2' or [enrol_case_identified_at]='3'	Please specify Dr. Name	text
	22	[child_diagnosed_by]	Child Diagnosed by:	dropdown<br>1 CHO<br>2 MO<br>3 ANM
	23	[child_classified_as]	Child classified as:	dropdown<br>1 PSBI<br>2 Sever Pneumonia<br>3 Pneumonia
	24	[household_name_enr]<br><br>Show the field ONLY if:<br>[child_available_2] = '1'	1. Name of household head:	text
	25	[caste_household_enr]<br><br>Show the field ONLY if:<br>[child_available_2] = '1'	2. What is the caste of household?	dropdown<br>11 Scheduled caste<br>12 Scheduled tribe<br>13 OBC<br>14 General<br>15 Other
	26	[religion_of_household]<br><br>Show the field ONLY if:<br>[child_available_2] = '1'	3. What is the religion of the head of household?	dropdown, Required<br>1 Hindu<br>2 Muslim<br>3 Sikh<br>4 Christian<br>5 Buddhist<br>6 Other
	27	[religion_other_specify]<br><br>Show the field ONLY if:	If other religion, specify	text

		[religion_of_household] ='6'		
	28	[no_of_persons_in_house]<br>Show the field ONLY if: [child_available_2] = '1'	4. What is the total number of people in the household?	text (number, Min: 1)
	29	[persons_slept_last_night]	5. How many persons slept in the house last night ?	text
	30	[no_of_child_less_than_5]<br>Show the field ONLY if: [child_available_2] = '1'	6. How many children less than 5 years of age typically sleep under the same roof as you?	text (number, Min: 1)
	31	[name_of_father]<br>Show the field ONLY if: [child_available_2] = '1'	7. Name of father	text, Required
	32	[age_of_father_in_years]<br>Show the field ONLY if: [child_available_2] = '1'	8. Age of father (in years)	text, Required
	33	[occupation_of_father]<br>Show the field ONLY if: [child_available_2] = '1'	9. What is the occupation of father?	dropdown, Required<br>1 Salaried worker (e.g. teacher, nurse, office)<br>2 Small business<br>3 Business owner<br>4 Skilled labor<br>5 Unskilled labor<br>6 Subsistence farming<br>7 Commercial farming<br>8 Fishing<br>9 Not applicable/Not working<br>10 Other
	34	[f_occupation_other_specify]<br>Show the field ONLY if: [occupation_of_father] = '10'	If other please specify	text, Required
	35	[name_of_mother]<br>Show the field ONLY if: [child_available_2] = '1'	10. Name of mother	text, Required
	36	[age_of_mother_in_years]<br>Show the field ONLY if: [child_available_2] = '1'	11. Age of mother (in years)	text, Required
	37	[mother_occupation]<br>Show the field ONLY if: [child_available_2] = '1'	12. What is mother's occupation?	dropdown, Required<br>1 Salaried worker (e.g. teacher, nurse, office)<br>2 Small business<br>3 Business owner

				4 Skilled labor<br>5 Unskilled labor<br>6 Subsistence farming<br>7 Commercial farming<br>8 Fishing<br>9 Not applicable/Not working<br>10 Other
	38	[m_occupation_other_specify]<br><br>Show the field ONLY if: [mother_occupation] = '10'	If other please specify	text, Required
	39	[total_monthly_income]<br><br>Show the field ONLY if: [child_available_2] = '1'	13. What is the total monthly income of the household?	text
	40	[type_of_house]<br><br>Show the field ONLY if: [child_available_2] = '1'	14. What is the type of house?	dropdown, Required<br>1 Kuccha<br>2 Pucca<br>3 Semi-pucca<br>4 Other
	41	[type_house_other_specify]<br><br>Show the field ONLY if: [type_of_house] = '4'	if other specify	text
	42	[total_no_of_rooms]<br><br>Show the field ONLY if: [child_available_2] = '1'	15. What is the total number of rooms in your house?	text, Required
	43	[total_no_of_windows]<br><br>Show the field ONLY if: [child_available_2] = '1'	16. What is the total number of windows in your house?	text
	44	[main_cooking_fuel]<br><br>Show the field ONLY if: [child_available_2] = '1'	17. What is the main cooking fuel used by the household?	checkbox, Required<br>1 main_cooking_fuel___1 LPG/ Natural gas<br>2 main_cooking_fuel___2 Kerosene<br>3 main_cooking_fuel___3 Coal<br>4 main_cooking_fuel___4 Charcoal<br>5 main_cooking_fuel___5 Wood<br>6 main_cooking_fuel___6 Dung cakes<br>7 main_cooking_fuel___7 Straw/ shrub/ grass<br>8 main_cooking_fuel___8 Agricultural crop waste<br>9 main_cooking_fuel___9 Biogas<br>10 main_cooking_fuel___10 Electric stove<br>11 main_cooking_fuel___11 Solar cooker

				12 | main_cooking_fuel___12 | Combination of any of the above<br>13 | main_cooking_fuel___13 | Other
	45	[cooking_fuel_other]<br>Show the field ONLY if: [main_cooking_fuel(13)]= 1	If other, please specify	text
	46	[place_of_cooking]<br>Show the field ONLY if: [child_available_2] = '1'	18. Where is the place of cooking?	dropdown, Required<br>1 | Inside house (no separate kitchen)<br>2 | Separate kitchen inside the house<br>3 | Outside the house<br>4 | Both inside and outside the house<br>5 | Separate kitchen outside the house<br>6 | Not applicable
	47	[separate_kitchen_window]<br>Show the field ONLY if: [place_of_cooking]='2'	If separate kitchen, does the kitchen have windows for ventilation?	yesno<br>1 | Yes<br>0 | No
	48	[main_source_of_water]<br>Show the field ONLY if: [child_available_2] = '1'	19. What is the main source of drinking-water for your household?	dropdown, Required<br>1 | Piped water into dwelling<br>2 | Public tap<br>3 | Tube-well or borehole or hand pump<br>4 | Open well<br>5 | Closed well<br>6 | Tanker truck<br>7 | Surface water (river/dam/lake/pond/stream/canal)<br>8 | Bottled water<br>9 | Rain water<br>10 | Combination of any of the above<br>11 | Other
	49	[water_source_other]<br>Show the field ONLY if: [main_source_of_water] = '11'	If other, please specify	text, Required
	50	[treatment_drinking_water]<br>Show the field ONLY if: [child_available_2] = '1'	20. What is the treatment you use before drinking water?	dropdown, Required<br>1 | Untreated<br>2 | Boiling<br>3 | Cloth filter<br>4 | Candle filter<br>5 | Reverse osmosis (RO) plant<br>6 | Other
	51	[water_treatment_other]<br>Show the field ONLY if:	If other please specify	text

		[treatment_drinking_water] = '6'		
	52	[main_garbage_disposal]<br>Show the field ONLY if:<br>[child_available_2] = '1'	21. What is the main method of garbage disposal used by your household?	dropdown, Required<br>1 Garbage dump<br>2 On the road<br>3 In drain/trench<br>4 In public pits<br>5 Panchayat/corporation services<br>6 Vacant plot<br>7 Burning<br>8 No designated place<br>9 Zamadarani
	53	[main_sewage_disposal]<br>Show the field ONLY if:<br>[child_available_2] = '1'	22. What is the main method of sewage disposal used by your household?	dropdown, Required<br>1 Municipal drainage system<br>2 Septic tanks<br>3 Connected to open drains<br>4 Let off into fields/ vacant plot<br>5 Other<br>6 Not applicable (for houses without toilets)
	54	[sewage_other]<br>Show the field ONLY if:<br>[main_sewage_disposal] = '5'	If other, please specify	text
	55	[kind_of_toilet_facility]<br>Show the field ONLY if:<br>[child_available_2] = '1'	23. What kind of toilet facility do members of your household usually use?	dropdown<br>1 Flush/pour flush to piped sewer system<br>2 Flush/pour flush to septic tank<br>3 Flush/pour flush to pit latrine<br>4 Flush/pour flush, don't know where<br>5 Ventilated improved pit latrine<br>6 Pit latrine with slab<br>7 Pit latrine without slab/open pit<br>8 Composting toilet<br>9 Bucket toilet<br>10 Hanging toilet/hanging latrine<br>11 No facility/bush/field<br>12 Other
	56	[toilet_kind_other]<br>Show the field ONLY if:<br>[kind_of_toilet_facility] = '13'	If other, please specify	text
	57	[electricity]<br>Show the field ONLY if:<br>[child_available_2] = '1'	Section Header: *Does your household have any of following amenities?*<br>24. Electricity	yesno, Required<br>1 Yes<br>0 No

	58	[internet_access]<br><br>Show the field ONLY if:<br>[child_available_2] = '1'	25. Internet access	yesno, Required<br>1 Yes<br>0 No
	59	[non_mobile_phone]<br><br>Show the field ONLY if:<br>[child_available_2] = '1'	26. Non-mobile phone/Landline	yesno<br>1 Yes<br>0 No
	60	[home_mobile_specify]<br><br>Show the field ONLY if:<br>[non_mobile_phone] = '1'	Please specify the phone number	text (number), Required
	61	[house_mobile]<br><br>Show the field ONLY if:<br>[child_available_2] = '1'	27. Does your household have a mobile telephone?	yesno, Required<br>1 Yes<br>0 No
	62	[mobile_home_specify]<br><br>Show the field ONLY if:<br>[house_mobile] = '1'	Please specify the mobile phone number	text (number, Min: 1000000000, Max: 9999999999)<br>Field Annotation: @FORCE-MINMAX
	63	[have_access_mobile]<br><br>Show the field ONLY if:<br>[child_available_2] = '1'	28. Do you have access to a mobile phone?	yesno, Required<br>1 Yes<br>0 No
	64	[yes_have_access]<br><br>Show the field ONLY if:<br>[have_access_mobile] = '1'		radio<br>1 Yes, I have my own mobile phone<br>2 Yes, but I share the mobile phone with other people
	65	[radio]<br><br>Show the field ONLY if:<br>[child_available_2] = '1'	Section Header: *Do you or any member of your household own any of the following items? [For quantities, use best estimate]*<br><br>29. Radio	yesno<br>1 Yes<br>0 No
	66	[television]<br><br>Show the field ONLY if:<br>[child_available_2] = '1'	30. Television	yesno<br>1 Yes<br>0 No
	67	[refrigerator]<br><br>Show the field ONLY if:<br>[child_available_2] = '1'	31. Refrigerator	yesno<br>1 Yes<br>0 No
	68	[computer]<br><br>Show the field ONLY if:<br>[child_available_2] = '1'	32. Computer	yesno<br>1 Yes<br>0 No
	69	[watch]<br><br>Show the field ONLY if:<br>[child_available_2] = '1'	33. Watch	yesno<br>1 Yes<br>0 No
	70	[bycycle]<br><br>Show the field ONLY if:<br>[child_available_2] = '1'	34. Bicycle	yesno<br>1 Yes<br>0 No
	71	[motorcycle]<br><br>Show the field ONLY if:<br>[child_available_2] = '1'	35. Motorcycle	yesno<br>1 Yes<br>0 No

	72	[motor_scooter]<br><br>Show the field ONLY if:<br>[child_available_2] = '1'	36. Motor Scooter	yesno<br>1 Yes<br>0 No
	73	[car_or_truck]<br><br>Show the field ONLY if:<br>[child_available_2] = '1'	37. Car or truck	yesno<br>1 Yes<br>0 No
	74	[boat_with_motor]<br><br>Show the field ONLY if:<br>[child_available_2] = '1'	38. Boat with motor	yesno<br>1 Yes<br>0 No
	75	[animal_drawn_cart]<br><br>Show the field ONLY if:<br>[child_available_2] = '1'	39. Animal drawn cart	yesno<br>1 Yes<br>0 No
	76	[own_agri_land]<br><br>Show the field ONLY if:<br>[child_available_2] = '1'	40. Does your household own any agricultural land?	yesno<br>1 Yes<br>0 No
	77	[own_farm_herd_poultry]<br><br>Show the field ONLY if:<br>[child_available_2] = '1'	Section Header: *Specify type of livestock, herds, farm animal/poultry*<br><br>41. Does your household own any livestock, herds, farm animals/poultry?	yesno<br>1 Yes<br>0 No
	78	[cattle]<br><br>Show the field ONLY if:<br>[own_farm_herd_poultry] = '1'	Cattle	yesno<br>1 Yes<br>0 No
	79	[goat]<br><br>Show the field ONLY if:<br>[own_farm_herd_poultry] = '1'	Goat	yesno<br>1 Yes<br>0 No
	80	[sheep]<br><br>Show the field ONLY if:<br>[own_farm_herd_poultry] = '1'	Sheep	yesno<br>1 Yes<br>0 No
	81	[poultry]<br><br>Show the field ONLY if:<br>[own_farm_herd_poultry] = '1'	Poultry	yesno<br>1 Yes<br>0 No
	82	[pigs]<br><br>Show the field ONLY if:<br>[own_farm_herd_poultry] = '1'	Pigs	yesno<br>1 Yes<br>0 No
	83	[donkey]<br><br>Show the field ONLY if:<br>[own_farm_herd_poultry] = '1'	Donkey	yesno<br>1 Yes<br>0 No
	84	[hourse]<br><br>Show the field ONLY if:<br>[own_farm_herd_poultry] = '1'	Hourse	yesno<br>1 Yes<br>0 No

	85	[other_animal_herd]<br><br>Show the field ONLY if: [own_farm_herd_poultry] = '1'	Other	yesno<br>1 Yes<br>0 No
	86	[other_herd_specify]<br><br>Show the field ONLY if: [other_animal_herd] = '1'	If other, please specify	text
	87	[prefer_seek_care]<br><br>Show the field ONLY if: [child_available_2] = '1'	Section Header: *Prefer to seek care location (We can ask in prefer number)*<br><br>42. Where does the family prefer to go to seek healthcare?	dropdown<br>1 Government Facility<br>2 Private Facility<br>3 At home<br>4 Other
	88	[please_specify_gov_facility]<br><br>Show the field ONLY if: [prefer_seek_care] = '1'	Please specify	text
	89	[specify_pvt_facility]<br><br>Show the field ONLY if: [prefer_seek_care] = '2'	Please specify	text
	90	[specify_other_facility]<br><br>Show the field ONLY if: [prefer_seek_care] = '4'	Please specify	text
	91	[who_makes_decision]<br><br>Show the field ONLY if: [child_available_2] = '1'	43. In the household, who usually makes the decision about where to go to seek care?	dropdown<br>1 Participant<br>2 Husband/partner<br>3 Participant and husband/partner jointly<br>4 Mother-in-law<br>5 Father-in-law<br>6 Other
	92	[decision_maker_other]<br><br>Show the field ONLY if: [who_makes_decision] = '6'	if other, please specify the decision maker's relationship to participant	text
	93	[asha]<br><br>Show the field ONLY if: [child_available_2] = '1'	Section Header: *41. Where did the family seek the treatment for Pneumonia?*<br><br>ASHA	checkbox<br>1 asha___1 1<br>2 asha___2 2<br>3 asha___3 3<br>4 asha___4 4<br>5 asha___5 5<br>6 asha___6 6
	94	[anm]<br><br>Show the field ONLY if: [child_available_2] = '1'	ANM	checkbox<br>1 anm___1 1<br>2 anm___2 2<br>3 anm___3 3

				4 anm___4 4<br>5 anm___5 5<br>6 anm___6 6
	95	[aww]<br>Show the field ONLY if:<br>[child_available_2] = '1'	AWW	checkbox<br>1 aww___1 1<br>2 aww___2 2<br>3 aww___3 3<br>4 aww___4 4<br>5 aww___5 5<br>6 aww___6 6
	96	[sub_center]<br>Show the field ONLY if:<br>[child_available_2] = '1'	Sub center	checkbox<br>1 sub_center___1 1<br>2 sub_center___2 2<br>3 sub_center___3 3<br>4 sub_center___4 4<br>5 sub_center___5 5<br>6 sub_center___6 6
	97	[health_and_wellness_center]<br>Show the field ONLY if:<br>[child_available_2] = '1'	Health and wellness center	checkbox<br>1 health_and_wellness_center___1 1<br>2 health_and_wellness_center___2 2<br>3 health_and_wellness_center___3 3<br>4 health_and_wellness_center___4 4<br>5 health_and_wellness_center___5 5<br>6 health_and_wellness_center___6 6
	98	[primary_health_centre]<br>Show the field ONLY if:<br>[child_available_2] = '1'	Primary Health Centre	checkbox<br>1 primary_health_centre___1 1<br>2 primary_health_centre___2 2<br>3 primary_health_centre___3 3<br>4 primary_health_centre___4 4<br>5 primary_health_centre___5 5<br>6 primary_health_centre___6 6
	99	[community_health_center]<br>Show the field ONLY if:<br>[child_available_2] = '1'	Community health center	checkbox<br>1 community_health_center___1 1<br>2 community_health_center___2 2<br>3 community_health_center___3 3<br>4 community_health_center___4 4<br>5 community_health_center___5 5<br>6 community_health_center___6 6
	100	[district_government_hospit]<br>Show the field ONLY if:<br>[child_available_2] = '1'	District/Government hospital/ Sub district hospital	checkbox<br>1 district_government_hospit___1 1<br>2 district_government_hospit___2 2<br>3 district_government_hospit___3 3

				<table><tr><td>4</td><td>district_government_hospit___4</td><td>4</td></tr><tr><td>5</td><td>district_government_hospit___5</td><td>5</td></tr><tr><td>6</td><td>district_government_hospit___6</td><td>6</td></tr></table>
	101	[chemist_shop]<br><br>Show the field ONLY if:<br>[child_available_2] = '1'	Chemist shop	checkbox<br><table><tr><td>1</td><td>chemist_shop___1</td><td>1</td></tr><tr><td>2</td><td>chemist_shop___2</td><td>2</td></tr><tr><td>3</td><td>chemist_shop___3</td><td>3</td></tr><tr><td>4</td><td>chemist_shop___4</td><td>4</td></tr><tr><td>5</td><td>chemist_shop___5</td><td>5</td></tr><tr><td>6</td><td>chemist_shop___6</td><td>6</td></tr></table>
	102	[private_practitioner_mbbs]<br><br>Show the field ONLY if:<br>[child_available_2] = '1'	Private practitioner (MBBS + specialization)	checkbox<br><table><tr><td>1</td><td>private_practitioner_mbbs___1</td><td>1</td></tr><tr><td>2</td><td>private_practitioner_mbbs___2</td><td>2</td></tr><tr><td>3</td><td>private_practitioner_mbbs___3</td><td>3</td></tr><tr><td>4</td><td>private_practitioner_mbbs___4</td><td>4</td></tr><tr><td>5</td><td>private_practitioner_mbbs___5</td><td>5</td></tr><tr><td>6</td><td>private_practitioner_mbbs___6</td><td>6</td></tr></table>
	103	[private_practitioner_medic]<br><br>Show the field ONLY if:<br>[child_available_2] = '1'	Private practitioner (Medical Doctor, MBBS)	checkbox<br><table><tr><td>1</td><td>private_practitioner_medic___1</td><td>1</td></tr><tr><td>2</td><td>private_practitioner_medic___2</td><td>2</td></tr><tr><td>3</td><td>private_practitioner_medic___3</td><td>3</td></tr><tr><td>4</td><td>private_practitioner_medic___4</td><td>4</td></tr><tr><td>5</td><td>private_practitioner_medic___5</td><td>5</td></tr><tr><td>6</td><td>private_practitioner_medic___6</td><td>6</td></tr></table>
	104	[private_practitioner_homeo]<br><br>Show the field ONLY if:<br>[child_available_2] = '1'	Private practitioner (Homeopathy, BHMS)	checkbox<br><table><tr><td>1</td><td>private_practitioner_homeo___1</td><td>1</td></tr><tr><td>2</td><td>private_practitioner_homeo___2</td><td>2</td></tr><tr><td>3</td><td>private_practitioner_homeo___3</td><td>3</td></tr><tr><td>4</td><td>private_practitioner_homeo___4</td><td>4</td></tr><tr><td>5</td><td>private_practitioner_homeo___5</td><td>5</td></tr><tr><td>6</td><td>private_practitioner_homeo___6</td><td>6</td></tr></table>
	105	[private_practitioner_ayurv]<br><br>Show the field ONLY if:<br>[child_available_2] = '1'	Private practitioner (Ayurveda, BAMS)	checkbox<br><table><tr><td>1</td><td>private_practitioner_ayurv___1</td><td>1</td></tr><tr><td>2</td><td>private_practitioner_ayurv___2</td><td>2</td></tr><tr><td>3</td><td>private_practitioner_ayurv___3</td><td>3</td></tr><tr><td>4</td><td>private_practitioner_ayurv___4</td><td>4</td></tr><tr><td>5</td><td>private_practitioner_ayurv___5</td><td>5</td></tr><tr><td>6</td><td>private_practitioner_ayurv___6</td><td>6</td></tr></table>
	106	[private_practitioner_rmp]<br><br>Show the field ONLY if:<br>[child_available_2] = '1'	Private practitioner (RMP)	checkbox<br><table><tr><td>1</td><td>private_practitioner_rmp___1</td><td>1</td></tr><tr><td>2</td><td>private_practitioner_rmp___2</td><td>2</td></tr><tr><td>3</td><td>private_practitioner_rmp___3</td><td>3</td></tr></table>

				4 | private_practitioner_rmp___4 | 4<br>5 | private_practitioner_rmp___5 | 5<br>6 | private_practitioner_rmp___6 | 6
	107	[private_practitioner_no_de]<br>Show the field ONLY if:<br>[child_available_2] = '1'	Private practitioner (No degree/ degree not known)	checkbox<br>1 | private_practitioner_no_de___1 | 1<br>2 | private_practitioner_no_de___2 | 2<br>3 | private_practitioner_no_de___3 | 3<br>4 | private_practitioner_no_de___4 | 4<br>5 | private_practitioner_no_de___5 | 5<br>6 | private_practitioner_no_de___6 | 6
	108	[private_nursing_home_hospi]<br>Show the field ONLY if:<br>[child_available_2] = '1'	Private nursing home/hospital	checkbox<br>1 | private_nursing_home_hospi___1 | 1<br>2 | private_nursing_home_hospi___2 | 2<br>3 | private_nursing_home_hospi___3 | 3<br>4 | private_nursing_home_hospi___4 | 4<br>5 | private_nursing_home_hospi___5 | 5<br>6 | private_nursing_home_hospi___6 | 6
	109	[not_sought]<br>Show the field ONLY if:<br>[child_available_2] = '1'	Not sought	checkbox<br>1 | not_sought___1 | 1<br>2 | not_sought___2 | 2<br>3 | not_sought___3 | 3<br>4 | not_sought___4 | 4<br>5 | not_sought___5 | 5<br>6 | not_sought___6 | 6
	110	[name_of_facility]<br>Show the field ONLY if:<br>[child_available_2] = '1'	44. If preference is other, Please specify	text
	111	[for_which_sign_and_symptom]<br>Show the field ONLY if:<br>[child_available_2] = '1'	45. For which symptoms family sought the treatment	checkbox, Required<br>1 | for_which_sign_and_symptom___1 | Unable to or drink<br>2 | for_which_sign_and_symptom___2 | Vomits<br>3 | for_which_sign_and_symptom___3 | Convulsio<br>4 | for_which_sign_and_symptom___4 | Unconsci<br>5 | for_which_sign_and_symptom___5 | Chest inc<br>6 | for_which_sign_and_symptom___6 | Fast brea<br>7 | for_which_sign_and_symptom___7 | Cough<br>8 | for_which_sign_and_symptom___8 | Cold<br>9 | for_which_sign_and_symptom___9 | Fever
	112	[referred_to_higher_facility]<br>Show the field ONLY if:<br>[child_available_2] = '1'	46. Was the child referred to higher facility for treatment of sever pneumonia?	yesno<br>1 | Yes<br>0 | No

	113	[asha_accompany_caregiver]<br><br>Show the field ONLY if:<br>[referred_to_higher_facility]='1'	47. Did ASHA accompany caregiver to referral facility?	yesno<br>1 Yes<br>0 No
	114	[ambulance_service_provided]<br><br>Show the field ONLY if:<br>[referred_to_higher_facility]='1'	48. Was ambulance service provided to child to visit referral facility?	yesno<br>1 Yes<br>0 No
	115	[ambulance_service_by]<br><br>Show the field ONLY if:<br>[ambulance_service_provided]='1'	Ambulance service provided by : -	dropdown<br>1 Family<br>2 CHW<br>3 other
	116	[reason_ambulance_not_available]<br><br>Show the field ONLY if:<br>[ambulance_service_provided]='0'	Reason why ambulance not provided ?	dropdown<br>1 Family refused to take ambulance.<br>2 Refused by CHW(Community health worker).<br>3 No facility of ambulance was available at facility.
	117	[prescrip_slip_available]<br><br>Show the field ONLY if:<br>[child_available_2] = '1'	49. Is prescription slip available ?	yesno<br>1 Yes<br>0 No
	118	[is_genta_advised_2]<br><br>Show the field ONLY if:<br>[child_available_2] = '1'	50. Is Gentamycin advised ?	yesno, Required<br>1 Yes<br>0 No
	119	[is_amox_advised_2]<br><br>Show the field ONLY if:<br>[child_available_2] = '1'	51. Is Amoxycillin advised?	yesno<br>1 Yes<br>0 No
	120	[amoxy_genta_table_2]	Advise Advise followed No of Days Dosage per day No of Days Dosage per day Amoxycillin {amox_receive_day_2} {amox_no_time_2} {amox_given_2} {amox_given_times_2} Gentamycin {genta_advise_day_2} {genta_no_times_2} {gentamycin_days_2} {genta_given_times_2}	descriptive
	121	[amox_receive_day_2]<br><br>Show the field ONLY if:<br>[is_amox_advised_2]='1'	If Amoxycillin advised, how many days did the child receive Amoxycillin	text (number)
	122	[amox_no_time_2]<br><br>Show the field ONLY if:<br>[is_amox_advised_2]='1'	If amoxicillin advised, number of times in a day it was advised for	text (number, Min: 1, Max: 12)
	123	[amox_given_2]<br><br>Show the field ONLY if:<br>[is_amox_advised_2]='1'	How many days did you give amoxicillin (Enter 0 if not given).	text (number, Min: 0, Max: 12)
	124	[amox_given_times_2]<br><br>Show the field ONLY if:	How many times in a day did you give amoxicillin to your infant	text (number, Min: 1)

		[is_amox_advised_2]=1		
	125	[genta_advise_day_2]<br>Show the field ONLY if:<br>[is_genta_advised_2]='1'	If Gentamycin advised, how many days did the child receive Gentamycin?	text, Required
	126	[genta_no_times_2]<br>Show the field ONLY if:<br>[is_genta_advised_2]='1'	If Gentamycin advised, number of times in a day it was advised for	text (number, Min: 1), Required
	127	[gentamycin_days_2]<br>Show the field ONLY if:<br>[is_genta_advised_2] = '1'	How many days did you give Gentamycin (Enter 0 if not given)	text (number), Required
	128	[genta_given_times_2]<br>Show the field ONLY if:<br>[is_genta_advised_2] = '1'	How many times in a day did you give Gentamycin to your infant ?	text, Required
	129	[other_medicine_advised_2]<br>Show the field ONLY if:<br>[child_available_2]='1'	52. Is there any other medicine given other than Amoxycillin/Gentamycin	yesno<br>1 Yes<br>0 No
	130	[what_other_advised_2]<br>Show the field ONLY if:<br>[other_medicine_advised_2]='1'	If yes, what was advised	text
	131	[amox_genta_conti_2]<br>Show the field ONLY if:<br>[child_available_2]='1'	53. Did you continue medication (amoxicillin/gentamycin) as per recommendation given by the health care providers	yesno, Required<br>1 Yes<br>0 No
	132	[home_treat_given_2]<br>Show the field ONLY if:<br>[child_available_2]='1'	54. Was any home treatment given for the illness ?	yesno, Required<br>1 Yes<br>0 No
	133	[yes_home_treated_2]<br>Show the field ONLY if:<br>[home_treat_given_2] = '1'	If yes what	radio, Required<br>1 Hot drink with ginger or lemon or tulsi leaves with or without sugar<br>2 Hot drink with ginger or lemon or mint with or without sugar<br>3 Tea with saunf or elaichi or ginger with or without sugar<br>4 Others
	134	[home_treatment_oth_2]<br>Show the field ONLY if:<br>[yes_home_treated_2] = 4	Specify other	text, Required
	135	[first_day_complete]	Section Header: *Form Status*<br>Complete?	dropdown<br>0 Incomplete<br>1 Unverified<br>2 Complete
**Instrument: Compliance Eighth Day (compliance_eighth_day)**				
	136	[date_of_form_filling_8]	Date of Form Filling:	text (datetime_dmy)
	137	[worker_code_8]	Project team member code	dropdown

				1 201 - Pancham<br>2 202 - Krishan Dagar<br>3 401 - Durg Pal<br>4 203 - Sandeep<br>5 402 - Prem Kishor<br>6 403 - Harphool Malik<br>7 204 - Rameshwar Sahu<br>8 205 - Yashpal<br>9 206 - Sunil<br>10 207 - Anuj<br>11 301 - Kavita<br>12 302 - Deepti<br>13 303 - Aarti
	138	[child_available_8]	Is the child available?	yesno, Required<br>1 Yes<br>0 No
	139	[child_not_reason_8]<br>Show the field ONLY if:<br>[child_available_8] = '0'	If child is not available, please select reason	dropdown, Required<br>1 Admit<br>2 Shifted out of study area<br>3 Death<br>4 Other
	140	[child_admit_place_8]<br>Show the field ONLY if:<br>[child_not_reason_8] = '1'	Where is the child admitted ?	text
	141	[child_shifted_place_8]<br>Show the field ONLY if:<br>[child_not_reason_8] = '2'	Where the child shifted?	text
	142	[date_of_death_8]<br>Show the field ONLY if:<br>[child_not_reason_8] = '3'	Date of Death	text (date_dmy)
	143	[summary_of_death_8]<br>Show the field ONLY if:<br>[child_not_reason_8] = '3'	Summary of Death	notes
	144	[child_not_reason_other_8]<br>Show the field ONLY if:<br>[child_not_reason_8] = '4'	Please specify other	text
	145	[is_genta_advised]<br>Show the field ONLY if:<br>[child_available_8] = '1'	1. Is Gentamycin advised ?	yesno, Required<br>1 Yes<br>0 No
	146	[is_amox_advised]<br>Show the field ONLY if:<br>[child_available_8] = '1'	2. Is Amoxycillin advised?	yesno<br>1 Yes<br>0 No

	147	[amoxy_genta_table]	Advise Advise followed No of Days Dosage per day No of Days Dosage per day Amoxycillin {amox_receive_day} {amox_no_time} {amox_given} {amox_given_times} Gentamycin {genta_advise_day} {genta_no_times} {gentamycin_days} {genta_given_times}	descriptive
	148	[amox_receive_day]<br><br>Show the field ONLY if:<br>[is_amox_advised]='1'	If Amoxycillin advised, how many days did the child receive Amoxycillin	text (number)
	149	[amox_no_time]<br><br>Show the field ONLY if:<br>[is_amox_advised]='1'	If amoxicillin advised, number of times in a day it was advised for	text (number, Min: 1, Max: 12)
	150	[amox_given]<br><br>Show the field ONLY if:<br>[is_amox_advised]='1'	How many days did you give amoxicillin (Enter 0 if not given).	text (number, Min: 0, Max: 12)
	151	[amox_given_times]<br><br>Show the field ONLY if:<br>[is_amox_advised]=1	How many times in a day did you give amoxicillin to your infant	text (number, Min: 1)
	152	[genta_advise_day]<br><br>Show the field ONLY if:<br>[is_genta_advised]='1'	If Gentamycin advised, how many days did the child receive Gentamycin?	text, Required
	153	[genta_no_times]<br><br>Show the field ONLY if:<br>[is_genta_advised]='1'	If Gentamycin advised, number of times in a day it was advised for	text (number, Min: 1), Required
	154	[gentamycin_days]<br><br>Show the field ONLY if:<br>[is_genta_advised] = '1'	How many days did you give Gentamycin (Enter 0 if not given)	text (number), Required
	155	[genta_given_times]<br><br>Show the field ONLY if:<br>[is_genta_advised] = '1'	How many times in a day did you give Gentamycin to your infant ?	text, Required
	156	[other_medicine_advised_8]<br><br>Show the field ONLY if:<br>[child_available_8]='1'	3. Is there any other medicine given other than Amoxycillin/Gentamycin	yesno<br>1 Yes<br>0 No
	157	[what_other_advised_8]<br><br>Show the field ONLY if:<br>[other_medicine_advised_8]='1'	If yes, what was advised	text
	158	[amox_genta_conti_cst_ch_1]<br><br>Show the field ONLY if:<br>[child_available_8]='1'	4. Did you continue medication (amoxicillin/gentamycin) as per recommendation given by the health care providers	yesno, Required<br>1 Yes<br>0 No
	159	[home_treat_given]<br><br>Show the field ONLY if:<br>[child_available_8]='1'	5. Was any home treatment given for the illness ?	yesno, Required<br>1 Yes<br>0 No
	160	[yes_home_treated]<br><br>Show the field ONLY if:<br>[home_treat_given] = '1'	If yes what	radio, Required<br>1 Hot drink with ginger or lemon or tulsi leaves with or without sugar

				2 Hot drink with ginger or lemon or mint with or without sugar<br>3 Tea with saunf or elaichi or ginger with or without sugar<br>4 Others
	161	[home_treatment_oth]<br>Show the field ONLY if:<br>[yes_home_treated] = 4	Specify other	text, Required
	162	[status_of_child]	6. What is the current status of child ?	dropdown, Required<br>1 Recovered<br>2 Not recovered/Still having symptoms
	163	[symp_on_eighth_day]<br>Show the field ONLY if:<br>[status_of_child]='2'	7. Does the child have one or more of the following signs:	dropdown<br>1 Unable to breastfeed or drink<br>2 Vomits<br>3 Convulsions/Lethargy<br>4 Unconscious<br>5 Chest indrawing<br>6 Fast breathing<br>7 Cough<br>8 Cold<br>9 Fever
	164	[compliance_eighth_day_complete]	Section Header: *Form Status*<br>Complete?	dropdown<br>0 Incomplete<br>1 Unverified<br>2 Complete
Instrument: **Sixteenth Day** (sixteenth_day)				
	165	[date_of_form_filling_16]	Date of Form Filling:	text (datetime_dmy)
	166	[worker_code_16]	Project team member code	dropdown<br>1 201 - Pancham<br>2 202 - Krishan Dagar<br>3 401 - Durg Pal<br>4 203 - Sandeep<br>5 402 - Prem Kishor<br>6 403 - Harphool Malik<br>7 204 - Rameshwar Sahu<br>8 205 - Yashpal<br>9 206 - Sunil<br>10 207 - Anuj<br>11 301 - Kavita<br>12 302 - Deepti<br>13 303 - Aarti
	167	[child_available_16]	1. Is the child available?	yesno

				1 Yes<br>0 No
	168	[child_not_reason_16]<br>Show the field ONLY if: [child_available_16] = '0'	If child is not available, please select reason	dropdown<br>1 Admit<br>2 Shifted out of study area<br>3 Death<br>4 Other
	169	[child_admit_place_16]<br>Show the field ONLY if: [child_not_reason_16] = '1'	Where is the child admitted ?	text
	170	[child_shifted_place_16]<br>Show the field ONLY if: [child_not_reason_16] = '2'	Where the child shifted?	text
	171	[child_not_reason_other_16]<br>Show the field ONLY if: [child_not_reason_2] = '4'	Please specify other	text
	172	[how_is_the_child_now]<br>Show the field ONLY if: [child_available_16]='1'	2. How is the child now ?	dropdown<br>1 Well<br>2 Unwell<br>3 Dead
	173	[date_of_death_16]<br>Show the field ONLY if: ([how_is_the_child_now] ='3') or ([child_not_reason_16]='3')	Date of Death	text (date_dmy)
	174	[symp_5_year_16]<br>Show the field ONLY if: [how_is_the_child_now] ='2'	3. Does the child have one or more of the following signs:	checkbox<br>1 symp_5_year_16___1 Unable to breastfeed or drink<br>2 symp_5_year_16___2 Vomits<br>3 symp_5_year_16___3 Convulsions/Lethargy<br>4 symp_5_year_16___4 Unconscious<br>5 symp_5_year_16___5 Chest indrawing<br>6 symp_5_year_16___6 Fast breathing<br>7 symp_5_year_16___7 Cough<br>8 symp_5_year_16___8 Cold<br>9 symp_5_year_16___9 None
	175	[does_the_child_hospitalize]<br>Show the field ONLY if: [child_available_16]='1'	4. Does the child hospitalized during the treatment period?	yesno, Required<br>1 Yes<br>0 No
	176	[hospitalization_days]<br>Show the field ONLY if:	for how many days ?	text (number, Min: 1), Required

		[does_the_child_hospitalize]='1'		
	177	[where_was_child_hospitalized]<br><br>Show the field ONLY if:<br>[does_the_child_hospitalize]='1'	where was the child hospitalized?	text
	178	[sixteenth_day_complete]	Section Header: *Form Status*<br><br>Complete?	dropdown<br>0 Incomplete<br>1 Unverified<br>2 Complete
