# Supplementary material for: Determinants and Experiences of Care‐Seeking for Childhood Pneumonia in a Rural Indian Setting: A Mixed‐Methods Study
Source: Health Expect. 2025 Apr 16;28(2):e70263. doi: 10.1111/hex.70263 (PMC12002083; doi:10.1111/hex.70263)
Supplement: Supplementary file 2 — Annexure II baselineform. [file HEX-28-e70263-s003.pdf]

Data Dictionary Codebook

IR - Pneumonia (PID: 15)

02-17-2025 15:48

Instruments		
**Instrument**	**Form Name**	**Events**
Base	base	baseline_arm_1
Unit	unit	baseline_arm_1
Child	child	baseline_arm_1
Hospitalization	hospitalization	baseline_arm_1

Events	
**Event Name**	**Unique event name**
Baseline	baseline_arm_1
Enrollment	enrollment_arm_1
Endline	endline_arm_1

	#	Variable / Field Name	Field Label *Field Note*	Field Attributes (Field Type, Validation, Choices, Calculations, etc.)
**Instrument: Base (base)**				
	1	[record_id]	Record ID	text
	2	[date_of_filling]	1. Date of Filling	text (datetime_seconds_dmy, Max: today), Required
	3	[chc]	2. CHC	dropdown, Required<br>1 CH PALWAL<br>2 Dudhola<br>3 ALAWAL PUR<br>4 Hathin<br>5 AURANGABAD<br>6 SDH HODAL<br>7 SONDHAD<br>9 Out of District
	4	[phc1]<br>Show the field ONLY if:<br>[chc] = 1	3. PHC	dropdown<br>1 CH PALWAL<br>2 Palwal city<br>3 Shyam nagar<br>4 Krishna colony
	5	[phc2]<br>Show the field ONLY if:<br>[chc] = 2	3. PHC	dropdown<br>5 Dudhola<br>6 Allika<br>7 RASULPUR
	6	[phc3]<br>Show the field ONLY if:<br>[chc] = 3	3. PHC	dropdown<br>8 Alawal pur<br>9 Amar pur<br>10 Sihol<br>11 Solra
	7	[phc4]<br>Show the field ONLY if:<br>[chc] = 4	3. PHC	dropdown<br>12 Hathin<br>13 Kalsada<br>14 Mandkola<br>15 Chhainsa<br>16 Uttawar

				17 Nagal Jat<br>18 Kot
	8	[phc5]<br>Show the field ONLY if:<br>[chc] = 5	3. PHC	dropdown<br>19 Aurangabad<br>20 Deeghot
	9	[phc6]<br>Show the field ONLY if:<br>[chc] = 6	3. PHC	dropdown<br>21 Hodal
	10	[phc7]<br>Show the field ONLY if:<br>[chc] = 7	3. PHC	dropdown<br>22 Sondhad<br>23 Bhulwana<br>24 Hassanpur<br>25 Tappa
	11	[phc9]<br>Show the field ONLY if:<br>[chc] = 9	3. PHC	dropdown<br>99 Out of District
	12	[village1]<br>Show the field ONLY if:<br>[phc1] = '1'	4. Village Name	dropdown<br>1 Omex city palwal<br>2 Sawal Vihar<br>3 Civil lines<br>4 Bhatia Cololny<br>5 Kailash appartment<br>6 G.H. Palwal<br>7 Panchwati Colony<br>8 Kalara Colony<br>9 Ekta Nagar<br>10 Krishna Colony Near Sohna road<br>11 Bass Colony<br>12 Parsuram Colony<br>13 Misson Colony<br>14 Takipura
	13	[village2]<br>Show the field ONLY if:<br>[phc1] = '2'	4. Village Name	dropdown<br>15 Khail Kalan<br>16 Khail Khurd<br>17 Neem talan<br>18 Thai Mohalla<br>19 Kanuka Mohalla<br>20 Kot Mohalla<br>21 Kumhaar Wada<br>22 Sariya Mohalla<br>23 Tola Mohalla<br>24 Shiv Puri<br>25 Paith Mohalla<br>26 Inder puri

				27 Gupta Ganj<br>28 Gorilla mohalla<br>29 Sayad Wada<br>30 Thakur wada<br>31 Line pura<br>32 Nangla Gulab<br>33 Dukariya<br>34 Jaindipura<br>35 Lakhi Vihar<br>36 Parjapati Mohalla<br>37 Sanjay Colony<br>38 Bhatia colony<br>39 H.B. Colony<br>40 Huda sec -2<br>41 Om Hospital
	14	[village3]<br><br>Show the field ONLY if:<br>[phc1] = '3'	4. Village Name	dropdown<br>42 Moti Colony<br>43 Arya Nagar<br>44 Saini Colony<br>45 Jawhar Nagar<br>46 Shiv Vihar<br>47 Sekhpura<br>48 Dev Nagar<br>49 Tuhiram Colony<br>50 Shyam Nagar<br>51 Geeta Colony<br>52 Bada Mohalla<br>53 Gandhi Ashram<br>54 Daya Basti<br>55 Dharam Nagar
	15	[village4]<br><br>Show the field ONLY if:<br>[phc1] = '4'	4. Village Name	dropdown<br>56 Nangla Lakki singh<br>57 Hanuman Garh<br>58 Basant Vihar<br>59 Shyam Vihar<br>60 I.T.I Palwal<br>61 Parkash Vihar<br>62 Kailash Nagar<br>63 Railway Colony<br>64 Mohan Nagar<br>65 Rajiv Nagar<br>66 Krishna Colony<br>67 Railway Colony 2

			68 Anaj Mandi<br>69 Friends Colony<br>70 Shiv Colony<br>71 Ram Nagar<br>72 Mangla Udyog<br>73 J.N. Rajpoot<br>74 J.Nagar Palwal<br>75 J.Nagar Camp<br>76 Bali Nagar<br>77 New Colony 8 marla<br>78 New Colony 4 marla<br>79 New Ext. colony<br>80 H.B.E.S Colony<br>81 Bye pass colony<br>82 Adarsh Colony<br>83 Nai basti Sallagarh<br>84 Sallagarh
16	[village5]<br>Show the field ONLY if:<br>[phc2] = '5'	4. Village Name	dropdown<br>85 Dudhola<br>86 N. Bhiku<br>87 Sikenderpur<br>88 Mandpuri<br>89 Kalwaka<br>90 kalwaka Dhani<br>91 Gadpuri<br>92 Chhaprola<br>93 Sehrala<br>94 Duandsha<br>95 Softa<br>96 Harphali<br>97 Prithla<br>98 Prithla Bhatta<br>99 Devli<br>100 Mandkol<br>101 Tatarpur<br>102 B.K.Bhatta<br>103 Baghola<br>104 Amru<br>105 Meerapur<br>106 Allahpur<br>107 Allahpur.Nangla<br>108 Allahapur SRS<br>109 Patli.Khurd

				110 Patli Kala<br>111 Patli. Old<br>112 Patli.Barhman<br>113 Firojpur<br>114 Agwanpur<br>115 Ashawatl 1<br>116 Jatola<br>117 Medapur
	17	[village6]<br>Show the field ONLY if:<br>[phc2] = '6'	4. Village Name	dropdown<br>118 Allika<br>119 Ghuhgera<br>120 Kisorpur<br>121 Kisorpur.Bass<br>122 Rajupur<br>123 Dhatir<br>124 Bhurja<br>125 Teharki<br>126 Jaindapur<br>127 Chandpur<br>128 Paroli<br>129 Dehlaka<br>130 Attarchatta<br>131 Dungarpur<br>132 Karna<br>133 Gailpur<br>134 Lalwa<br>135 Rajolka<br>136 Bamnika<br>137 Meghpur<br>138 Kakrali<br>139 N. Gwar<br>140 Patli shukiram<br>141 Maheshpur<br>142 Yadupur<br>143 Karaka<br>144 Badha<br>145 Rakhota<br>146 Johar Khera
	18	[village7]<br>Show the field ONLY if:<br>[phc2] = '7'	4. Village Name	dropdown<br>147 Rasulpur<br>148 Farijanpur Khedla<br>149 A.Dakora<br>150 Hosangabad

				151 Asawata<br>152 Chajjunagar<br>153 Muner Ghari<br>154 Hari nagar<br>155 Loha Garh<br>156 Nagla lohagarh<br>157 Rohnija<br>158 Chirawata<br>159 Rehrana<br>160 Dholagarh<br>161 Kashipur<br>162 Kuslipur 1<br>163 Kuslipur 2
	19	[village8]<br>Show the field ONLY if:<br>[phc3] = '8'	4. Village Name	dropdown<br>164 Alawal pur<br>165 Janoli<br>166 Fazalpur<br>167 Basant garh<br>168 Ramgarh<br>169 Kithwari<br>170 Shamsha Bad<br>171 Islama bad<br>172 Kateshra<br>173 Ghaghot<br>174 Gopi Khera<br>175 Sadarpur<br>176 Kakripur<br>177 N.kakri pur<br>178 N.Nai<br>179 Dadhota<br>180 Lalpura<br>181 Badram<br>182 Khajurka<br>183 Kurara Shapur<br>184 N.Azad<br>185 N.Basanta
	20	[village9]<br>Show the field ONLY if:<br>[phc3] = '9'	4. Village Name	dropdown<br>186 Amarpur<br>187 Kulena / MAKSOODPUR<br>188 Jalaka<br>189 Thanthri<br>190 Hasapur<br>191 Balai

				192 Rampur Khor<br>193 Nagla khor<br>194 N.Ghori<br>195 Sujwari<br>196 Chirwari<br>197 Ghori<br>198 Phaledpur<br>199 Gurwari<br>200 N.Gurwari
	21	[village10]<br>Show the field ONLY if:<br>[phc3] = '10'	4. Village Name	dropdown<br>201 Sihol<br>202 Pelak<br>203 Malik Gannika<br>204 Chant<br>205 Missa<br>206 Taraka<br>207 Jeetgarh
	22	[village11]<br>Show the field ONLY if:<br>[phc3] = '11'	4. Village Name	dropdown<br>208 Solra<br>209 Bolra<br>210 Rajupur<br>211 Dostpur<br>212 M.S.Faram<br>213 Pehruka<br>214 Sohan.F<br>215 Dewan.F<br>216 Jeewan.F<br>217 Baghpur<br>218 Bhambhuka<br>219 Sunehrika<br>220 Bhood<br>221 Khedli<br>222 Shekpur<br>223 Jeet.F<br>224 Panchyat<br>225 Jhupa<br>226 Nagaliya<br>227 Focal.H
	23	[village12]<br>Show the field ONLY if:<br>[phc4] = '12'	4. Village Name	dropdown<br>228 Hathin<br>229 Andhrola<br>230 Guraksar<br>231 Khilluka

				232 Mithaka<br>233 Kondal<br>234 Mohdemka<br>235 Gahlab<br>236 B.Pahadi<br>237 Jalalpur<br>238 Dhiranki
	24	[village13]<br>Show the field ONLY if:<br>[phc4] = '13'	4. Village Name	dropdown<br>239 Bhanguri<br>240 Malokhara<br>241 Mamolaka<br>242 F.P.Rajput<br>243 Garhi Vinoda<br>244 Sapanki<br>245 Bichpuri<br>246 Kalsada<br>247 Bhamrola<br>248 Rindka<br>249 Hathin Bass<br>250 Tikri Brahman<br>251 Durgapur<br>252 Raipur<br>253 Ratipur<br>254 Jodhpur<br>255 Aherwan<br>256 Dhamaka<br>257 Rajpura<br>258 Jalalpur<br>259 Nagli
	25	[village14]<br>Show the field ONLY if:<br>[phc4] = '14'	4. Village Name	dropdown<br>260 Mandkola<br>261 Syaroli<br>262 Kanoli<br>263 Khedli Jeeta<br>264 Mandkola B<br>265 Pondri<br>266 Reeber<br>267 N.Bad<br>268 Mandkola C<br>269 Mandnaka<br>270 Mandori<br>271 Aluka<br>272 Gharrot

				273 Khokiyaka<br>274 Janacholi<br>275 Puthli<br>276 Jainpur
	26	[village15]<br>Show the field ONLY if:<br>[phc4] = '15'	4. Village Name	dropdown<br>277 Bhighawali<br>278 Meerpur<br>279 Durenchi<br>280 Mathepur<br>281 A.P.Natol<br>282 Swamika<br>283 Chhainsa<br>284 Ghigdaka<br>285 Hudithal<br>286 Buraka<br>287 Khedli Brahman<br>288 Ladmaki<br>289 Mankaki<br>290 Babupur<br>291 Ransika<br>292 Mahaluka<br>293 H.P Kalan<br>294 H.P.Khurd
	27	[village16]<br>Show the field ONLY if:<br>[phc4] = '16'	4. Village Name	dropdown<br>295 Uttawar<br>296 Ghudawali<br>297 Kumrehda<br>298 Tonka<br>299 Uttawae B<br>300 Malai<br>301 Malai Bag<br>302 Bheemsika<br>303 Dhakalpur<br>304 Guleshra<br>305 Rupraka<br>306 Bhudpur<br>307 Jarali<br>308 Malpuri<br>309 Gohpur<br>310 Chilli<br>311 Chilla<br>312 Pachanka<br>313 Garhi Hathin

				314 Immam Colony<br>315 Lakhnaka<br>316 Paharpur<br>317 Kukarchati<br>318 Islam Bass
	28	[village17]<br>Show the field ONLY if:<br>[phc4] = '17'	4. Village Name	dropdown<br>319 Nagal Jat<br>320 Manpur<br>321 Pahadi<br>322 Andhop<br>323 Khaika<br>324 Nagal Sabha<br>325 Bahin
	29	[village18]<br>Show the field ONLY if:<br>[phc4] = '18'	4. Village Name	dropdown<br>326 Ali Meo<br>327 Ali Brahman<br>328 Paosar<br>329 Natoli<br>330 Kot<br>331 Jhanda<br>332 Maluka
	30	[village19]<br>Show the field ONLY if:<br>[phc5] = '19'	4. Village Name	dropdown<br>333 AURANGABAD<br>334 GOPALGARH<br>335 N.BHAMARI<br>336 N.BAJHARA<br>337 GUDHRANA<br>338 TUMESRA<br>339 MITROL<br>340 SEVLI<br>341 SARAI<br>342 N.AHSANPUR<br>343 KHATELA<br>344 N.Rahimpur<br>345 PHOOLWARI<br>346 BAHROLA<br>347 ATOHAN<br>348 BAMNIKHERA<br>349 MANSHA GREEN<br>350 RAILWAY COLONY<br>351 SUGERMILL<br>352 SRINAGAR
	31	[village20]	4. Village Name	dropdown

		Show the field ONLY if: [phc5] = '20'		353 SELOTHI<br>354 NANGAL BRAHMAN<br>355 HIDAYAT PUR<br>356 RUNDHI<br>357 LADIYAKA<br>358 BATA<br>359 N.SAPERA<br>360 DEEGHOT<br>361 N.DEEGHOT<br>362 RAIDASKA<br>363 BAMARIYAKA<br>364 DUNGERWAS<br>365 AJIJABAD<br>366 BELA<br>367 ALIGARH<br>368 KANWARKA<br>369 PINGORE<br>370 NANGLA PINGORE<br>371 SIHA<br>372 NANGLA SIHA
	32	[village21]<br>Show the field ONLY if: [phc6] = '21'	4. Village Name	dropdown<br>373 HODAL-1<br>374 DADKA<br>375 BORAKA<br>376 URBAN HODAL<br>377 GARHI PATTI<br>378 HODAL-2
	33	[village22]<br>Show the field ONLY if: [phc7] = '22'	4. Village Name	dropdown<br>379 SONDH<br>380 LOHINA<br>381 KN LOHINA<br>382 KN SONDH<br>383 BANCHARI<br>384 DAKORA<br>385 PENGALTU<br>386 J.P MAFI<br>387 MARROLI<br>388 SHOLAKA<br>389 PALRI<br>390 NAGLA BABAJI
	34	[village23]<br>Show the field ONLY if: [phc7] = '23'	4. Village Name	dropdown<br>391 BHULWANA<br>392 KARMAN

				393 GOROTA<br>394 BEDHA<br>395 BANSWA<br>396 KHIRBI<br>397 SHASHAI<br>398 VIJAYGARH
	35	[village24]<br>Show the field ONLY if:<br>[phc7] = '24'	4. Village Name	dropdown<br>399 Hasanpur<br>400 N.Parsa<br>401 N.Ashref<br>402 N.Satica<br>403 Jattoli<br>404 Attarchtta<br>405 Laharpur<br>406 Satuaghari<br>407 N.Kanpur<br>408 N.Moruka<br>409 Khambi<br>410 Bhoop Garh<br>411 Nakhrola<br>412 Ghasara<br>413 Likhi<br>414 Machipura<br>415 N.ILLabad<br>416 Bhandoli<br>417 Sandoli<br>418 Ramgarh<br>419 Darana<br>420 Maholi<br>421 Bhiduki
	36	[village25]<br>Show the field ONLY if:<br>[phc7] = '25'	4. Village Name	dropdown<br>422 KUSHAK<br>423 N.SAPERA<br>424 ACHHEJA<br>425 ALEHABAD/BADKA<br>426 MUSTAFABAD/KHATKA<br>427 SAMSHTIPUR/INDRA NAGAR<br>428 MOBLIPUR<br>429 SULTANPUR<br>430 RAHIMPUR<br>431 N.RAHIMPUR<br>432 TAPPA<br>433 FATNAGAR

				434 N. SORUKA<br>435 KASHIPUR<br>436 ATWA<br>437 BILOCHPUR<br>438 PIRGARHI<br>439 N.SEHDEV<br>440 N.SURJAN<br>441 BADOLI<br>442 LALGARH<br>443 AYA NAGAR<br>444 AMROLI<br>445 BHAWANA<br>446 LULWARI<br>447 KAMRAWALI<br>448 T GURJJAR<br>449 NANDAWALA<br>450 N CHAWAN<br>451 GULAWAD<br>452 MIRPUR KORALI<br>453 INAYATPUR<br>454 N. MOHMMADPUR<br>455 N.NAGALA<br>456 KARIMPUR
	37	[village999]<br>Show the field ONLY if:<br>[phc9] = '99'	4. Village Name	dropdown<br>999 Out of District
	38	[worker_code]	5. Worker Code	dropdown, Required<br>1 201 - Pancham(00001-00400)<br>2 202 - Krishan Dagar(00401-00800)<br>3 401 - Durg pal (00801-01200)<br>4 402 - Bhupesh(01201-01600)<br>5 403 - Prem Kishor(01601-02000)<br>6 404 - Harphool(02001-02400)<br>7 405 - Rameshwar Sahu(02401-02800)<br>8 406 - Yashpal(02801-03200)<br>9 407 - Sunil(03201-03600)
	39	[id]	ID	text (number, Min: 0001), Required<br>Field Annotation: @CHARLIMIT=4
	40	[household_id]	6. Household_ID	calc, Required<br>Calculation: [chc]*10000000000 + if([phc1]>0, [phc1]*100000000,0)+ if([phc2]>0, [phc2]*100000000,0)+ if([phc3]>0, [phc3]*100000000,0)+ if([phc4]>0, [phc4]*100000000,0)+ if([phc5]>0, [phc5]*100000000,0)+ if([phc6]>0, [phc6]*100000000,0)+ if([phc7]>0,

				[phc7]*100000000,0)+ if([phc9]>0, [phc9]*100000000,0)+ if([village1]>0, [village1]*100000,0)+ if([village2]>0, [village2]*100000,0)+ if([village3]>0, [village3]*100000,0)+ if([village4]>0, [village4]*100000,0)+ if([village5]>0, [village5]*100000,0)+ if([village6]>0, [village6]*100000,0)+ if([village7]>0, [village7]*100000,0)+ if([village8]>0, [village8]*100000,0)+ if([village9]>0, [village9]*100000,0)+ if([village10]>0, [village10]*100000,0)+ if([village11]>0, [village11]*100000,0)+ if([village12]>0, [village12]*100000,0)+ if([village13]>0, [village13]*100000,0)+ if([village14]>0, [village14]*100000,0)+ if([village15]>0, [village15]*100000,0)+ if([village16]>0, [village16]*100000,0)+ if([village17]>0, [village17]*100000,0)+ if([village18]>0, [village18]*100000,0)+ if([village19]>0, [village19]*100000,0)+ if([village20]>0, [village20]*100000,0)+ if([village21]>0, [village21]*100000,0)+ if([village22]>0, [village22]*100000,0)+ if([village23]>0, [village23]*100000,0)+ if([village24]>0, [village24]*100000,0)+ if([village25]>0, [village25]*100000,0)+ if([village999]>0, [village999]*100000,0)+[id]
	41	[street_no]	7. Street No.<br>*0 - if NA*	text (number), Required
	42	[house_no]	8. House No.<br>*Fill 0 if N/A*	text (number), Required
	43	[ward_no]	9. Ward No<br>*Fill 0 if N/A*	text (number)
	44	[address_landmark]	10. Address + Landmark	notes, Required
	45	[info_avail]	11. Is any Informant available at house?	yesno, Required<br>1 Yes<br>0 No
	46	[info_avail_reason]<br>Show the field ONLY if:<br>[info_avail] = '0'	12. If no please select reason	dropdown, Required<br>1 House Locked<br>2 No one answered<br>3 Refusal<br>4 Other
	47	[info_avail_reason_oth]<br>Show the field ONLY if:<br>[info_avail_reason] = '4'	if other, then specify	text
	48	[primary_contact_number]<br>Show the field ONLY if:<br>[info_avail] = '1'	Primary Contact Number	text (number, Min: 1000000000, Max: 9999999999), Required<br>Field Annotation: @CHARLIMIT=10
	49	[name_of_head_of_the_househ]<br>Show the field ONLY if:<br>[info_avail] = '1'	13. Name of Head of the Household	text, Required
	50	[type_of_family]<br>Show the field ONLY if:	14. Type of Family	dropdown (autocomplete), Required<br>3 Nuclear

		[info_avail] = '1'		4 Joint<br>5 Three Generation
	51	[no_family_members]<br><br>Show the field ONLY if:<br>[info_avail] = '1'	15. Number of Family Members in the Household	text (number, Min: 1, Max: 100), Required
	52	[no_person_slept]<br><br>Show the field ONLY if:<br>[info_avail] = '1'	16. Number of Persons who stay in the house last night	text (number, Min: 2, Max: 100), Required<br>Field Annotation: @CHARLIMIT=2
	53	[extra_person_last_night]<br><br>Show the field ONLY if:<br>[info_avail] = '1'	Extra number of persons more then the family members who stayed last night in the house?	calc, Required<br>Calculation: [no_person_slept] - [no_family_members]
	54	[no_days_extra_per_stay]<br><br>Show the field ONLY if:<br>[extra_person_last_night] > 0	From how many days extra persons are staying in the house?	text (number), Required
	55	[assets_in_the_home]<br><br>Show the field ONLY if:<br>[info_avail] = '1'	Section Header: *ASSETS IN THE HOME*<br><br>17. ASSETS IN THE HOME	checkbox, Required<br>1 assets_in_the_home___1 Electricity<br>2 assets_in_the_home___2 A mattress<br>3 assets_in_the_home___3 A pressure cooker<br>4 assets_in_the_home___4 A chair<br>5 assets_in_the_home___5 A cot or bed<br>6 assets_in_the_home___6 A table<br>7 assets_in_the_home___7 An electric fan (even not working one)<br>8 assets_in_the_home___8 A radio or transistor (even not working one)<br>9 assets_in_the_home___9 A black and white television (even not working one)<br>10 assets_in_the_home___10 A color television (even not working one)<br>11 assets_in_the_home___11 A sewing machine (even not working one)<br>12 assets_in_the_home___12 A mobile telephone (even not working one)<br>13 assets_in_the_home___13 A landline telephone (even not working one)

				14 | assets_in_the_home___14 | Internet<br>15 | assets_in_the_home___15 | A computer (even not working one)<br>16 | assets_in_the_home___16 | A refrigerator (even not working one)<br>17 | assets_in_the_home___17 | An air conditioner/ cooler<br>18 | assets_in_the_home___18 | A Washing machine<br>19 | assets_in_the_home___19 | A watch or clock (even not working one)<br>20 | assets_in_the_home___20 | A bicycle (even not working one)<br>21 | assets_in_the_home___21 | A motorcycle or scooter (even not working one)<br>22 | assets_in_the_home___22 | An animal-drawn cart (even not working one)<br>23 | assets_in_the_home___23 | A car (even not working one)<br>24 | assets_in_the_home___24 | A water pump (even not working one)<br>25 | assets_in_the_home___25 | A thresher (even not working one)<br>26 | assets_in_the_home___26 | A tractor (even not working one)
	56	[fuel_type_cooking]<br>Show the field ONLY if: [info_avail] = '1'	18. What type of fuel does the household mainly use for cooking ?	radio, Required<br>11 | Electricity<br>12 | Liquid petroleum gas / Natural gas<br>13 | kerosene<br>14 | Coal / Lignite<br>15 | Charcoal<br>16 | Wood<br>17 | Straw /Shrub / Grass<br>18 | Agricultural crop waste<br>19 | Dung cakes<br>20 | Biogas<br>21 | Others
	57	[fuel_cooking_other]	If cooking fuel is "other", specify	text, Required

		Show the field ONLY if: [fuel_type_cooking] = 21		
	58	[main_floor_material]<br><br>Show the field ONLY if: [info_avail] = '1'	19. Main material of the floor	radio, Required<br>11 Mud/clay/earth<br>12 Sand<br>13 Dung<br>14 Raw wood planks<br>15 Palm/bamboo<br>16 Brick<br>17 Stone<br>18 Parquet or polished wood<br>19 Vinyl or asphalt<br>20 Ceramic tiles<br>21 Cement<br>22 Polished stone/marble/granite<br>23 other
	59	[main_floor_other]<br><br>Show the field ONLY if: [main_floor_material] = 24	If main material of the floor is "other", specify	text, Required
	60	[main_roof_material]<br><br>Show the field ONLY if: [info_avail] = '1'	20. Main material of the roof	radio, Required<br>11 No roof<br>12 Thatch/ palm leaf/reed/grass<br>13 Mud<br>14 Sod/mud and grass mixture<br>15 Plastic/polythene sheeting<br>16 Rustic mat<br>17 Palm/bamboo<br>18 Raw wood planks/timber<br>19 Unburnt brick<br>20 Loosely packed stone<br>21 Metal<br>22 Wood<br>23 Calamine/ cement fiber<br>24 Asbestos sheets<br>25 Reinforced cement concrete (RCC)/Reinforced brick concrete/cement/ concrete<br>26 Roofing shingles<br>27 Tiles<br>28 Slate<br>29 burnt brick<br>30 Gatter Pather<br>31 Other
	61	[main_roof_other]<br><br>Show the field ONLY if:	If main material of the roof is "other", specify	text, Required

		[main_roof_material] = 31		
	62	[main_mat_ext_wall]<br><br>Show the field ONLY if:<br>[info_avail] = '1'	21. Main material of the exterior walls	radio, Required<br>11 No walls<br>12 Cane/palm/trunks/ bamboo<br>13 Mud<br>14 Grass/ reeds/ thatch<br>15 Bamboo with mud<br>16 Stone with mud<br>17 Plywood<br>18 Cardboard<br>19 Unburnt brick<br>20 Raw wood/reused wood<br>21 Cement/concrete<br>22 Stone with lime/cement<br>23 Burnt bricks<br>24 Cement blocks<br>25 Wood planks/ shingles<br>26 Galvanized Iron/Metal/ asbestos sheets<br>27 Other
	63	[main_mat_ext_wall_oth]<br><br>Show the field ONLY if:<br>[main_mat_ext_wall] = 27	If main material of the exterior walls is "other", specify	text, Required
	64	[do_you_own_this_house]<br><br>Show the field ONLY if:<br>[info_avail] = '1'	22. Do you own this house?	yesno, Required<br>1 Yes<br>0 No
	65	[no_of_family_units]<br><br>Show the field ONLY if:<br>[info_avail] = '1'	23. Number of family units	text (number, Min: 1), Required<br>Field Annotation: @CHARLIMIT=1
	66	[family_unit_number]<br><br>Show the field ONLY if:<br>[no_of_family_units] > 0	Section Header: *Household Information to be filled for each family unit*<br><br>Family unit number	calc<br>Calculation: if([no_of_family_units] > 0,1,'')
	67	[head_unit_name_1]<br><br>Show the field ONLY if:<br>[no_of_family_units] > 0	Name of Head of the unit	text, Required
	68	[no_of_child_age_0_6_1]<br><br>Show the field ONLY if:<br>[no_of_family_units] > 0	(i). Number of children, age 0-6 days	text (number), Required<br>Field Annotation: @CHARLIMIT=1
	69	[no_of_infant_age_7_5_1]<br><br>Show the field ONLY if:<br>[no_of_family_units] > 0	(ii). Number of Infants aged 7-59 days	text (number), Required<br>Field Annotation: @CHARLIMIT=1
	70	[no_of_child_age_5y_1]<br><br>Show the field ONLY if:<br>[no_of_family_units] > 0	(iii). Number of children aged 2 to 59 months	text, Required<br>Field Annotation: @CHARLIMIT=1

	71	[num_age_less_than_59]<br><br>Show the field ONLY if:<br>[no_of_family_units] > 0	Number of children, age < 59 months in this family unit	calc, Required<br>Calculation: sum([no_of_child_age_0_6_1] , [no_of_infant_age_7_5_1] , [no_of_child_age_5y_1])
	72	[family_unit_number_2]<br><br>Show the field ONLY if:<br>[no_of_family_units] > 1	Family unit number	calc<br>Calculation: if([no_of_family_units] > 1,2,'')
	73	[head_unit_name_2]<br><br>Show the field ONLY if:<br>[no_of_family_units] > 1	Name of Head of the unit	text, Required
	74	[no_of_child_age_0_6_2]<br><br>Show the field ONLY if:<br>[no_of_family_units] > 1	(i). Number of children, age 0-6 days	text (number), Required
	75	[no_of_infant_age_7_5_2]<br><br>Show the field ONLY if:<br>[no_of_family_units] > 1	(ii). Number of Infants aged 7-59 days	text (number), Required
	76	[no_of_child_age_5y_2]<br><br>Show the field ONLY if:<br>[no_of_family_units] > 1	(iii). Number of children aged 2 to 59 months	text (number), Required
	77	[num_age_less_than_59_2]<br><br>Show the field ONLY if:<br>[no_of_family_units] > 1	Number of children, age < 59 months in this family unit	calc, Required<br>Calculation: sum([no_of_child_age_0_6_2] + [no_of_infant_age_7_5_2] + [no_of_child_age_5y_2])
	78	[family_unit_number_3]<br><br>Show the field ONLY if:<br>[no_of_family_units] > 2	Family unit number	calc<br>Calculation: if([no_of_family_units] > 2,3,'')
	79	[head_unit_name_3]<br><br>Show the field ONLY if:<br>[no_of_family_units] > 2	Name of Head of the unit	text, Required
	80	[no_of_child_age_0_6_3]<br><br>Show the field ONLY if:<br>[no_of_family_units] > 2	(i). Number of children, age 0-6 days	text (number), Required
	81	[no_of_infant_age_7_5_3]<br><br>Show the field ONLY if:<br>[no_of_family_units] > 2	(ii). Number of Infants aged 7-59 days	text (number), Required
	82	[no_of_child_age_5y_3]<br><br>Show the field ONLY if:<br>[no_of_family_units] > 2	(iii) Number of children aged 2 to 59 months	text (number), Required
	83	[num_age_less_than_59_3]<br><br>Show the field ONLY if:<br>[no_of_family_units] > 2	Number of children, age < 59 months in this family unit	calc, Required<br>Calculation: sum([no_of_child_age_0_6_3] , [no_of_infant_age_7_5_3] , [no_of_child_age_5y_3])
	84	[family_unit_number_4]<br><br>Show the field ONLY if:<br>[no_of_family_units] > 3	Family unit number	calc<br>Calculation: if([no_of_family_units] > 3,4,'')
	85	[head_unit_name_4]<br><br>Show the field ONLY if:<br>[no_of_family_units] > 3	Name of Head of the unit	text, Required
	86	[no_of_child_age_0_6_4]<br><br>Show the field ONLY if:	(i). Number of children, age 0-6 days	text (number), Required

		[no_of_family_units] > 3		
	87	[no_of_infant_age_7_5_4]<br>Show the field ONLY if:<br>[no_of_family_units] > 3	(ii). Number of Infants aged 7-59 days	text (number), Required
	88	[no_of_child_age_5y_4]<br>Show the field ONLY if:<br>[no_of_family_units] > 3	(iii). Number of children aged 2 to 59 months	text (number), Required
	89	[num_age_less_than_59_4]<br>Show the field ONLY if:<br>[no_of_family_units] > 3	Number of children, age < 59 months in this family unit	calc, Required<br>Calculation: sum([no_of_child_age_0_6_4] , [no_of_infant_age_7_5_4] , [no_of_child_age_5y_4])
	90	[family_unit_number_5]<br>Show the field ONLY if:<br>[no_of_family_units] > 4	Family unit number	calc<br>Calculation: if([no_of_family_units] > 4,5,'')
	91	[head_unit_name_5]<br>Show the field ONLY if:<br>[no_of_family_units] > 4	Name of Head of the unit	text, Required
	92	[no_of_child_age_0_6_5]<br>Show the field ONLY if:<br>[no_of_family_units] > 4	(i). Number of children, age 0-6 days	text (number), Required
	93	[no_of_infant_age_7_5_5]<br>Show the field ONLY if:<br>[no_of_family_units] > 4	(ii). Number of Infants aged 7-59 days	text (number), Required
	94	[no_of_child_age_5y_5]<br>Show the field ONLY if:<br>[no_of_family_units] > 4	(iii). Number of children aged 2 to 59 months	text (number), Required
	95	[num_age_less_than_59_5]<br>Show the field ONLY if:<br>[no_of_family_units] > 4	Number of children, age < 59 months in this family unit	calc, Required<br>Calculation: sum([no_of_child_age_0_6_5] , [no_of_infant_age_7_5_5] , [no_of_child_age_5y_5])
	96	[eligible_units]<br>Show the field ONLY if:<br>[info_avail] = '1'	No of Eligible Units	calc<br>Calculation: if([num_age_less_than_59]>0,1,0) + if([num_age_less_than_59_2]>0,1,0) + if([num_age_less_than_59_3]>0,1,0) + if([num_age_less_than_59_4]>0,1,0) + if([num_age_less_than_59_5]>0,1,0)
	97	[base_complete]	Section Header: *Form Status*<br>Complete?	dropdown<br>0 Incomplete<br>1 Unverified<br>2 Complete
**Instrument: Unit (unit)**				
	98	[informant]	1. Informant	dropdown, Required<br>11 Mother<br>12 Father<br>13 Other family members<br>14 Other guardian/caretakers<br>15 Both father and mother
	99	[if_informant_other]<br>Show the field ONLY if:	If informant other, please specify	text

		[informant] = '13' or [informant] = '14'		
	100	[mother_not_present_reason]<br><br>Show the field ONLY if: [informant] = '12' or [informant] = '13' or [informant] = '14'	Please specify the reason why mother is not present at house?	text, Required
	101	[name_of_father]	2. Name of father	text, Required
	102	[father_age]	3. Father's age	text (number, Min: 0, Max: 99), Required
	103	[father_schooling]<br><br>Show the field ONLY if: [father_age] > 0	4. Father's year of schooling<br>*Fill 0 if father expired*	dropdown, Required<br>1 less than primary(5th) / Illiterate<br>2 Primary(5th)<br>3 Above Primary(5th) but less than secondary(10th)<br>4 Secondary(10th)<br>5 Higher secondary(12th)/ Diploma<br>6 Graduation<br>7 Post graduation<br>8 Above post graduation
	104	[father_occupation]<br><br>Show the field ONLY if: [father_age] > 0	5. Father's current occupation	dropdown, Required<br>11 Government Service<br>12 Private service<br>13 Self-employed<br>14 Farming only<br>15 Daily wage<br>16 Does not work<br>99 Not applicable
	105	[father_religion]	6. Father's religion	dropdown, Required<br>11 Christian<br>12 Muslim<br>13 Hindu<br>14 Sikh<br>15 Buddhist/neo-Buddhist<br>16 Jain<br>17 None<br>18 other
	106	[father_religion_oth]<br><br>Show the field ONLY if: [father_religion] = 18	If other, specify	text, Required
	107	[ethnic_caste_father]	7. Ethnic group (caste/tribe) of father	dropdown<br>11 Scheduled caste<br>12 Scheduled tribe<br>13 OBC<br>14 Unreserved<br>15 None of them

	108	[secondary_contact_number]	Secondary Contact Number	text (number, Min: 1000000000, Max: 9999999999)<br>Field Annotation: @CHARLIMIT=10
	109	[relation_of_secondary_cont]<br><br>Show the field ONLY if:<br>[secondary_contact_number] > 1000000000	Relation of secondary contact	dropdown, Required<br>1 Uncle<br>2 Aunty<br>3 Grand father<br>4 Grand mother<br>5 Father<br>6 Mother<br>7 Other
	110	[if_other_please_specify]<br><br>Show the field ONLY if:<br>[relation_of_secondary_cont] = '7'	If other, please specify	text, Required
	111	[mother_age]	8. Mother/primary caregivers age (years)	text (number, Min: 0, Max: 99), Required
	112	[mother_schooling]<br><br>Show the field ONLY if:<br>[mother_age] > 0	9. Mother or primary caregiver's years of schooling	dropdown, Required<br>1 less than primary(5th) / Illiterate<br>2 Primary(5th)<br>3 Above Primary(5th) but less than secondary(10th)<br>4 Secondary(10th)<br>5 Higher secondary(12th)/ Diploma<br>6 Graduation<br>7 Post graduation<br>8 Above post graduation
	113	[mother_occupation]<br><br>Show the field ONLY if:<br>[mother_age] > 0	10. Mother or primary caregivers current occupation	dropdown, Required<br>11 Government service<br>12 Private service<br>13 Self-employed<br>14 Farming only<br>15 Daily wage earner<br>16 Does not work<br>18 Home Maker
	114	[child_food]	11. Does the child consume the following food	checkbox, Required<br>1 child_food___1 Eggs<br>2 child_food___2 Milk<br>3 child_food___3 Mutton<br>4 child_food___4 Fish<br>5 child_food___5 Chicken<br>6 child_food___6 Other animal meat<br>7 child_food___7 Fruits<br>8 child_food___8 Vegetables

	115	[main_water_source]	12. What is the main source of drinking water for members of your household?	dropdown, Required<br>11 Piped water<br>12 Public tap<br>13 Tube well or borehole or hand pump<br>14 Open well<br>15 Closed well<br>16 Water from spring<br>17 Rain water<br>18 Tanker truck<br>19 Small cart with tank<br>20 Surface water [river/dam/lake/ pond/ stream/canal]<br>21 Bottled water<br>22 Other
	116	[main_water_source_oth]<br>Show the field ONLY if:<br>[main_water_source] = 22	If source of water is "other", specify	text
	117	[ro_filter_available]	13. Is the family uses R.O filtration ?	yesno<br>1 Yes<br>0 No
	118	[ro_use_since_from_when]<br>Show the field ONLY if:<br>[ro_filter_available] = '1'	If yes, Since from when	radio, Required<br>1 Less then one year<br>2 1 - 2 years<br>3 2 - 3 years<br>4 3 - 4 years<br>5 4 - 5 years<br>6 More than 5 years
	119	[type_of_toilet]	14. Type of toilet	dropdown, Required<br>3 Independent<br>4 Shared with other households<br>5 Open/No-toilet
	120	[kind_of_toilet]<br>Show the field ONLY if:<br>[type_of_toilet] = '3' or [type_of_toilet] = '4'	15. What kind of toilet facility available in house?	radio, Required<br>11 Flush or pour flush toilet<br>12 Pit latrine<br>13 Dry toilet<br>15 Twin pit/Composting toilet<br>16 No toilet facility/ Open space<br>17 Other
	121	[kind_toilet_other]<br>Show the field ONLY if:<br>[kind_of_toilet] = 17	If toilet facility is "other", specify	text, Required
	122	[bank_acc]	16. Does any usual member of this household have a bank account?	yesno<br>1 Yes<br>0 No

	123	[post_off_acc]	17. Does any usual member of this household have a post office account?	yesno<br>1 Yes<br>0 No
	124	[possess_bpl_card]	18. Possess a below poverty line card (BPL card)	yesno, Required<br>1 Yes<br>0 No
	125	[health_insurance]	19. Is any usual member of this household covered by a health scheme or health insurance?	yesno, Required<br>1 Yes<br>0 No
	126	[type_insurance]<br>Show the field ONLY if: [health_insurance] = '1'	Please select the scheme : -	checkbox, Required<br>1 type_insurance___1 Ayushman Bharat Scheme<br>2 type_insurance___2 CGHS<br>3 type_insurance___3 ECHS<br>4 type_insurance___4 ESI<br>5 type_insurance___5 Private
	127	[specify_private_insurance]<br>Show the field ONLY if: [type_insurance(5)] = '1'	Please specify (name of company), if insurance covered by private company	text, Required
	128	[name_of_person_insured]<br>Show the field ONLY if: [health_insurance] = '1'	20. Name of person insured under insurance scheme ?	text, Required
	129	[no_of_member_covered]<br>Show the field ONLY if: [health_insurance] = '1'	21. Number of members covered under insurance scheme	text (number), Required
	130	[take_ayushman_benefit]<br>Show the field ONLY if: [type_insurance(1)] = '1'	22. Did you take any benefit under Ayushman Bharat Scheme?	yesno, Required<br>1 Yes<br>0 No
	131	[when_you_take_benefit]<br>Show the field ONLY if: [take_ayushman_benefit] = '1'	When did you take the benefit of Ayushman Bharat Scheme ?	text (date_dmy), Required
	132	[for_what_disease]<br>Show the field ONLY if: [take_ayushman_benefit] = '1'	And for what disease ?	text, Required
	133	[annual_income]	23. Annual Income	text (number), Required
	134	[unit_complete]	Section Header: *Form Status*<br>Complete?	dropdown<br>0 Incomplete<br>1 Unverified<br>2 Complete
**Instrument: Child (child)**				
	135	[child_ill_prev_month]	Did the children have any illnesses any time in the previous month?	yesno, Required<br>1 Yes<br>0 No

	136	[name_child_ill_1]<br>Show the field ONLY if:<br>[child_ill_prev_month] = '1'	1. Name	text, Required, Identifier
	137	[father_name_child]<br>Show the field ONLY if:<br>[child_ill_prev_month] = '1'	2. Name of Father	text, Required
	138	[sex]<br>Show the field ONLY if:<br>[child_ill_prev_month] = '1'	3. Gender	radio, Required<br>3 Male<br>4 Female
	139	[dob]<br>Show the field ONLY if:<br>[child_ill_prev_month] = '1'	4. Date of birth	text (date_dmy, Min: 2017-01-01, Max: 2024-01-01), Required
	140	[age_of_child]<br>Show the field ONLY if:<br>[child_ill_prev_month] = '1'	5. Age of child (in days)	calc, Required<br>Calculation: datediff ([dob],[date_of_filling], "d","dmy",true)
	141	[is_child_lbw_1]<br>Show the field ONLY if:<br>[child_ill_prev_month] = '1'	6. Was the child born low birth weight?<br>*Weight less than 2.5 kg*	yesno, Required<br>1 Yes<br>0 No
	142	[weight_child_lbw_1]<br>Show the field ONLY if:<br>[is_child_lbw_1] = 1	7. Weight of the child in grams	text (number, Min: 200, Max: 2500), Required<br>Field Annotation: @CHARLIMIT = 4
	143	[was_child_lbw_preterm_1]<br>Show the field ONLY if:<br>[is_child_lbw_1] = 1	8. Was the child born preterm ?	yesno, Required<br>1 Yes<br>0 No
	144	[child_gest_age_in_weeks_1]<br>Show the field ONLY if:<br>[is_child_lbw_1] = 1	9. Gestational age at birth in weeks	text (number, Min: 20, Max: 38), Required
	145	[date_sym_iden]<br>Show the field ONLY if:<br>[child_ill_prev_month] = '1'	10. When did symptoms were identified ?	text (date_dmy), Required
	146	[age_when_symptoms_ident]<br>Show the field ONLY if:<br>[child_ill_prev_month] = '1'	Age of child when symptoms were identified?	calc<br>Calculation: datediff([dob], [date_sym_iden],"d","dmy",true)
	147	[age_ch_sym_iden_days_1]<br>Show the field ONLY if:<br>[child_ill_prev_month] = '1'	Number of days before symptoms were identified ?	calc, Required<br>Calculation: [age_of_child] - [age_when_symptoms_ident]
	148	[cough_ch_1]<br>Show the field ONLY if:<br>[child_ill_prev_month] = '1'	11. Cough	yesno, Required<br>1 Yes<br>0 No<br>Custom alignment: RH
	149	[cough_rep_ch_1]<br>Show the field ONLY if:<br>[cough_ch_1] = 1	Illness reported	radio, Required<br>3 Spontaneous<br>4 Prompted
	150	[cough_days]	From how many days?	text (number)

		Show the field ONLY if: [cough_ch_1] = '1'		
	151	[cough_persists] Show the field ONLY if: [cough_ch_1] = 1	Duration for which symptom persists?	text (number), Required
	152	[cold_running_nose_ch_1] Show the field ONLY if: [child_ill_prev_month] = '1'	12. Cold/running nose	yesno, Required<br>1 Yes<br>0 No<br>Custom alignment: RH
	153	[cold_rep_ch_1] Show the field ONLY if: [cold_running_nose_ch_1] = 1	Illness reported	radio, Required<br>3 Spontaneous<br>4 Prompted
	154	[day_run_nose_ch_1] Show the field ONLY if: [cold_running_nose_ch_1] = 1	From how many days	text (number, Min: 1), Required
	155	[difficult_breathing_ch_1] Show the field ONLY if: [child_ill_prev_month] = '1'	13. Difficult breathing	yesno, Required<br>1 Yes<br>0 No<br>Custom alignment: RH
	156	[diff_breath_ch_1] Show the field ONLY if: [difficult_breathing_ch_1] = 1	Illness reported	radio, Required<br>3 Spontaneous<br>4 Prompted
	157	[diff_breath_days] Show the field ONLY if: [difficult_breathing_ch_1] = '1'	From how many days?	text
	158	[days_diff_breath_ch_1] Show the field ONLY if: [difficult_breathing_ch_1] = 1	Duration for which symptom persists?	text (number)
	159	[pasli_chalna_ch_1] Show the field ONLY if: [child_ill_prev_month] = '1'	14. Chest indrawing / Pasli chalna	yesno, Required<br>1 Yes<br>0 No<br>Custom alignment: RH
	160	[pasli_rep_ch_1] Show the field ONLY if: [pasli_chalna_ch_1] = 1	Illness reported	radio, Required<br>3 Spontaneous<br>4 Prompted
	161	[pasli_chalna_days] Show the field ONLY if: [pasli_chalna_ch_1] = '1'	From how many days?	text (number)
	162	[days_pasli_ch_1] Show the field ONLY if: [pasli_chalna_ch_1] = 1	Duration for which symptom persists?	text (number)
	163	[fast_breathing_ch_1]	15. Fast breathing	yesno, Required

		Show the field ONLY if: [child_ill_prev_month] = '1'		1 Yes<br>0 No<br><br>Custom alignment: RH
	164	[fast_breath_rep_ch_1]<br><br>Show the field ONLY if: [fast_breathing_ch_1] = 1	Illness reported	radio, Required<br>3 Spontaneous<br>4 Prompted
	165	[fast_breath_days]<br><br>Show the field ONLY if: [fast_breathing_ch_1] = '1'	From how many days	text
	166	[fast_breath_duration]<br><br>Show the field ONLY if: [fast_breathing_ch_1] = '1'	Duration for which symptom persists?	text (number)
	167	[wheezing_ch_1]<br><br>Show the field ONLY if: [child_ill_prev_month] = '1'	16. Wheezing	yesno, Required<br>1 Yes<br>0 No<br><br>Custom alignment: RH
	168	[wheezing_rep_ch_1]<br><br>Show the field ONLY if: [wheezing_ch_1] = 1	Illness reported	radio, Required<br>3 Spontaneous<br>4 Prompted
	169	[days_wheezing_ch_1]<br><br>Show the field ONLY if: [wheezing_ch_1] = 1	From how many days	text (number, Min: 1), Required
	170	[stop_feed_ch_1]<br><br>Show the field ONLY if: [child_ill_prev_month] = '1'	17. Not able to breastfeed/drink/stop feeding	yesno, Required<br>1 Yes<br>0 No<br><br>Custom alignment: RH
	171	[stop_feed_rep_ch_1]<br><br>Show the field ONLY if: [stop_feed_ch_1] = 1	Illness reported	radio, Required<br>3 Spontaneous<br>4 Prompted
	172	[days_stop_feed_1]<br><br>Show the field ONLY if: [stop_feed_ch_1] = 1	From how many days	text (number, Min: 1), Required
	173	[vomiting_ch_1]<br><br>Show the field ONLY if: [child_ill_prev_month] = '1'	18. Vomiting	yesno, Required<br>1 Yes<br>0 No<br><br>Custom alignment: RH
	174	[vomit_rep_ch_1]<br><br>Show the field ONLY if: [vomiting_ch_1] = 1	Illness reported	radio, Required<br>3 Spontaneous<br>4 Prompted
	175	[days_vomiting_1]<br><br>Show the field ONLY if: [vomiting_ch_1] = 1	From how many days	text (number, Min: 1), Required
	176	[convulsions_ch_1]	19. Convulsions	yesno, Required

		Show the field ONLY if: [child_ill_prev_month] = '1'		1 Yes<br>0 No<br><br>Custom alignment: RH
	177	[illness_rep_ch_17]<br><br>Show the field ONLY if: [convulsions_ch_1] = 1	Illness reported	radio, Required<br>3 Spontaneous<br>4 Prompted
	178	[days_convuls_ch_1]<br><br>Show the field ONLY if: [convulsions_ch_1] = 1	From how many days	text (number, Min: 1), Required
	179	[lethargic_ch_1]<br><br>Show the field ONLY if: [child_ill_prev_month] = '1'	20. lethargic	yesno, Required<br>1 Yes<br>0 No<br><br>Custom alignment: RH
	180	[illness_rep_ch_18]<br><br>Show the field ONLY if: [lethargic_ch_1] = 1	Illness reported	radio, Required<br>3 Spontaneous<br>4 Prompted
	181	[days_lethargic_ch_1]<br><br>Show the field ONLY if: [lethargic_ch_1] = 1	From how many days	text (number, Min: 1), Required
	182	[unconscious_ch_1]<br><br>Show the field ONLY if: [child_ill_prev_month] = '1'	21. Unconscious	yesno, Required<br>1 Yes<br>0 No<br><br>Custom alignment: RH
	183	[uncon_rep_ch_1]<br><br>Show the field ONLY if: [unconscious_ch_1] = 1	Illness reported	radio, Required<br>3 Spontaneous<br>4 Prompted
	184	[days_unconscious_ch_1]<br><br>Show the field ONLY if: [unconscious_ch_1] = 1	From how many days	text (number, Min: 1), Required
	185	[stirdor_ch_1]<br><br>Show the field ONLY if: [child_ill_prev_month] = '1'	22. Stirdor (Sound in breathing)	yesno, Required<br>1 Yes<br>0 No<br><br>Custom alignment: RH
	186	[stirdor_rep_ch_1]<br><br>Show the field ONLY if: [stirdor_ch_1] = 1	Illness reported	radio, Required<br>3 Spontaneous<br>4 Prompted
	187	[days_stirdor_ch_1]<br><br>Show the field ONLY if: [stirdor_ch_1] = 1	From how many days	text (number, Min: 1), Required
	188	[temp_high_ch_1]<br><br>Show the field ONLY if: [child_ill_prev_month] = '1'	23. Temperature more than 37.5?C	yesno, Required<br>1 Yes<br>0 No

				Custom alignment: RH
	189	[temp_high_rep_ch_1]<br>Show the field ONLY if:<br>[temp_high_ch_1] = 1	Illness reported	radio, Required<br>3 Spontaneous<br>4 Prompted
	190	[days_temp_high_ch_1]<br>Show the field ONLY if:<br>[temp_high_ch_1] = 1	From how many days	text (number, Min: 1), Required
	191	[hypothermia_ch_1]<br>Show the field ONLY if:<br>[child_ill_prev_month] = '1'	24. Low body temperature / Hypothermia	yesno, Required<br>1 Yes<br>0 No<br>Custom alignment: RH
	192	[hypotherm_rep_ch_1]<br>Show the field ONLY if:<br>[hypothermia_ch_1] = 1	Illness reported	radio, Required<br>3 Spontaneous<br>4 Prompted
	193	[day_hypotherm_ch_1]<br>Show the field ONLY if:<br>[hypothermia_ch_1] = 1	From how many days	text (number, Min: 1), Required
	194	[spo2_ch_1]<br>Show the field ONLY if:<br>[child_ill_prev_month] = '1'	25. spO2 < 90%	yesno, Required<br>1 Yes<br>0 No<br>Custom alignment: RH
	195	[spo2_rep_ch_1]<br>Show the field ONLY if:<br>[spo2_ch_1] = 1	Illness reported	radio, Required<br>3 Spontaneous<br>4 Prompted
	196	[day_spo2_ch_2]<br>Show the field ONLY if:<br>[spo2_ch_1] = 1	From how many days	text (number, Min: 1), Required
	197	[diarrhea_ch_1]<br>Show the field ONLY if:<br>[child_ill_prev_month] = '1'	26. Diarrhea	yesno, Required<br>1 Yes<br>0 No<br>Custom alignment: RH
	198	[diarrhea_rep_ch_1]<br>Show the field ONLY if:<br>[diarrhea_ch_1] = 1	Illness reported	radio, Required<br>3 Spontaneous<br>4 Prompted
	199	[days_diarrhea_ch_1]<br>Show the field ONLY if:<br>[diarrhea_ch_1] = 1	From how many days	text (number, Min: 1), Required
	200	[dehydration_ch_1]<br>Show the field ONLY if:<br>[child_ill_prev_month] = '1'	27. Dehydration	yesno, Required<br>1 Yes<br>0 No<br>Custom alignment: RH
	201	[dehydrat_rep_ch_1]	Illness reported	radio, Required

		Show the field ONLY if: [dehydration_ch_1] = 1		3 Spontaneous<br>4 Prompted
	202	[days_dehydration_ch_1]<br><br>Show the field ONLY if: [dehydration_ch_1] = 1	From how many days	text (number, Min: 1), Required
	203	[sunken_eyes_ch_1]<br><br>Show the field ONLY if: [child_ill_prev_month] = '1'	28. Sunken eyes	yesno, Required<br>1 Yes<br>0 No<br><br>Custom alignment: RH
	204	[sunken_rep_ch_1]<br><br>Show the field ONLY if: [sunken_eyes_ch_1] = 1	Illness reported	radio, Required<br>3 Spontaneous<br>4 Prompted
	205	[days_sunken_eye_ch_1]<br><br>Show the field ONLY if: [sunken_eyes_ch_1] = 1	From how many days	text (number, Min: 1), Required
	206	[restless_irritable_ch_1]<br><br>Show the field ONLY if: [child_ill_prev_month] = '1'	29. Restless/Irritable	yesno, Required<br>1 Yes<br>0 No<br><br>Custom alignment: RH
	207	[restless_rep_ch_1]<br><br>Show the field ONLY if: [restless_irritable_ch_1] = 1	Illness reported	radio, Required<br>3 Spontaneous<br>4 Prompted
	208	[days_rest_irritable_ch_1]<br><br>Show the field ONLY if: [restless_irritable_ch_1] = 1	From how many days	text (number, Min: 1), Required
	209	[blood_in_stool_ch_1]<br><br>Show the field ONLY if: [child_ill_prev_month] = '1'	30. Blood in stool	yesno, Required<br>1 Yes<br>0 No<br><br>Custom alignment: RH
	210	[blood_rep_ch_1]<br><br>Show the field ONLY if: [blood_in_stool_ch_1] = 1	Illness reported	radio, Required<br>3 Spontaneous<br>4 Prompted
	211	[days_blood_stool_ch_1]<br><br>Show the field ONLY if: [blood_in_stool_ch_1] = 1	From how many days	text (number, Min: 1), Required
	212	[stiff_neck_ch_1]<br><br>Show the field ONLY if: [child_ill_prev_month] = '1'	31. Stiff neck	yesno, Required<br>1 Yes<br>0 No<br><br>Custom alignment: RH
	213	[stiff_neck_rep_ch_1]<br><br>Show the field ONLY if:	Illness reported	radio, Required<br>3 Spontaneous

		[stiff_neck_ch_1] = 1		4 Prompted
	214	[days_stiff_neck_ch_1]<br>Show the field ONLY if:<br>[stiff_neck_ch_1] = 1	From how many days	text (number, Min: 1), Required
	215	[eye_discharge_ch_1]<br>Show the field ONLY if:<br>[child_ill_prev_month] = '1'	32. Eye discharge	yesno, Required<br>1 Yes<br>0 No<br>Custom alignment: RH
	216	[eye_dis_rep_ch_1]<br>Show the field ONLY if:<br>[eye_discharge_ch_1] = 1	Illness reported	radio, Required<br>3 Spontaneous<br>4 Prompted
	217	[days_eye_dis_ch_1]<br>Show the field ONLY if:<br>[eye_discharge_ch_1] = 1	From how many days	text (number, Min: 1), Required
	218	[ear_discharge_ch_1]<br>Show the field ONLY if:<br>[child_ill_prev_month] = '1'	33. Ear discharge	yesno, Required<br>1 Yes<br>0 No<br>Custom alignment: RH
	219	[ear_dis_rep_ch_1]<br>Show the field ONLY if:<br>[ear_discharge_ch_1] = 1	Illness reported	radio, Required<br>3 Spontaneous<br>4 Prompted
	220	[days_ear_dis_ch_1]<br>Show the field ONLY if:<br>[ear_discharge_ch_1] = 1	From how many days	text (number, Min: 1), Required
	221	[skin_infections_ch_1]<br>Show the field ONLY if:<br>[child_ill_prev_month] = '1'	34. Skin infections	yesno, Required<br>1 Yes<br>0 No<br>Custom alignment: RH
	222	[skin_inf_rep_ch_1]<br>Show the field ONLY if:<br>[skin_infections_ch_1] = 1	Illness reported	radio, Required<br>3 Spontaneous<br>4 Prompted
	223	[days_skin_inf_ch_1]<br>Show the field ONLY if:<br>[skin_infections_ch_1] = 1	From how many days	text (number, Min: 1), Required
	224	[bulging_fontanelle_ch_1]<br>Show the field ONLY if:<br>[child_ill_prev_month] = '1'	35. Bulging fontanelle	yesno, Required<br>1 Yes<br>0 No<br>Custom alignment: RH
	225	[bulg_font_rep_ch_1]<br>Show the field ONLY if:<br>[bulging_fontanelle_ch_1] = 1	Illness reported	radio, Required<br>3 Spontaneous<br>4 Prompted
	226	[days_bulg_ch_1]	From how many days	text (number, Min: 1), Required

		Show the field ONLY if: [bulging_fontanelle_ch_1] = 1		
	227	[malnutrition_ch_1]<br><br>Show the field ONLY if: [child_ill_prev_month] = '1'	36. Very weak / malnutrition	yesno, Required<br>1 Yes<br>0 No<br><br>Custom alignment: RH
	228	[mal_nutri_rep_ch_1]<br><br>Show the field ONLY if: [malnutrition_ch_1] = 1	Illness reported	radio, Required<br>3 Spontaneous<br>4 Prompted
	229	[day_mal_nut_ch_1]<br><br>Show the field ONLY if: [malnutrition_ch_1] = 1	From how many days	text (number, Min: 1), Required
	230	[measles_ch_1]<br><br>Show the field ONLY if: [child_ill_prev_month] = '1'	37. Measles	yesno, Required<br>1 Yes<br>0 No<br><br>Custom alignment: RH
	231	[measles_rep_ch_1]<br><br>Show the field ONLY if: [measles_ch_1] = 1	Illness reported	radio, Required<br>3 Spontaneous<br>4 Prompted
	232	[days_measles_ch_1]<br><br>Show the field ONLY if: [measles_ch_1] = 1	From how many days	text (number, Min: 1), Required
	233	[mouth_ulcer_ch_1]<br><br>Show the field ONLY if: [child_ill_prev_month] = '1'	38. Mouth ulcer	yesno, Required<br>1 Yes<br>0 No<br><br>Custom alignment: RH
	234	[ulcer_rep_ch_1]<br><br>Show the field ONLY if: [mouth_ulcer_ch_1] = 1	Illness reported	radio, Required<br>3 Spontaneous<br>4 Prompted
	235	[day_mouth_ul_ch_1]<br><br>Show the field ONLY if: [mouth_ulcer_ch_1] = 1	From how many days	text (number, Min: 1), Required
	236	[umbilicus_red_ch_1]<br><br>Show the field ONLY if: [child_ill_prev_month] = '1'	39. Umbilicus red or draining pus	yesno, Required<br>1 Yes<br>0 No<br><br>Custom alignment: RH
	237	[umbilicus_rep_ch_1]<br><br>Show the field ONLY if: [umbilicus_red_ch_1] = 1	Illness reported	radio, Required<br>3 Spontaneous<br>4 Prompted
	238	[days_umbilic_ch_1]<br><br>Show the field ONLY if: [umbilicus_red_ch_1] = 1	From how many days	text (number, Min: 1), Required
	239	[skin_pustules_ch_1]	40. Skin pustules	yesno, Required

		Show the field ONLY if:<br>[child_ill_prev_month] = '1'		1 Yes<br>0 No<br><br>Custom alignment: RH
	240	[pustules_rep_ch_1]<br><br>Show the field ONLY if:<br>[skin_pustules_ch_1] = 1	Illness reported	radio, Required<br>3 Spontaneous<br>4 Prompted
	241	[day_pustules_ch_1]<br><br>Show the field ONLY if:<br>[skin_pustules_ch_1] = 1	From how many days	text (number, Min: 1), Required
	242	[yellow_soles_ch_1]<br><br>Show the field ONLY if:<br>[child_ill_prev_month] = '1'	41. Yellow soles	yesno, Required<br>1 Yes<br>0 No<br><br>Custom alignment: RH
	243	[yel_soles_ch_1]<br><br>Show the field ONLY if:<br>[yellow_soles_ch_1] = 1	Illness reported	radio, Required<br>3 Spontaneous<br>4 Prompted
	244	[day_yell_sole_ch_1]<br><br>Show the field ONLY if:<br>[yellow_soles_ch_1] = 1	From how many days	text (number, Min: 1), Required
	245	[others_illness_ch_1]<br><br>Show the field ONLY if:<br>[child_ill_prev_month] = '1'	42. Others	yesno, Required<br>1 Yes<br>0 No<br><br>Custom alignment: RH
	246	[other_rep_ch_1]<br><br>Show the field ONLY if:<br>[others_illness_ch_1] = 1	Illness reported	radio, Required<br>3 Spontaneous<br>4 Prompted
	247	[days_oth_ill_ch_1]<br><br>Show the field ONLY if:<br>[others_illness_ch_1] = 1	From how many days	text (number, Min: 1), Required
	248	[specify_other_ch_1]<br><br>Show the field ONLY if:<br>[others_illness_ch_1] = 1	Specify other	text, Required<br>Custom alignment: RH
	249	[desc_1]<br><br>Show the field ONLY if:<br>([cough_ch_1] = '1') and ([difficult_breathing_ch_1] ='1' or [fast_breathing_ch_1] = '1' or [pasli_chalna_ch_1] = '1' or [stirdor_ch_1] = '1' or [wheezing_ch_1] = '1')	If the child had cough with fast breathing/difficult breathing/chest indrawing/stridor/wheezing ask for the following :-	descriptive
	250	[dur_ill_stop_feed_ch_1]<br><br>Show the field ONLY if:<br>([cough_ch_1] = '1') and ([difficult_breathing_ch_1] ='1' or [fast_breathing_ch_1] = '1' or [pasli_chalna_ch	43. Was the child unable to feed or had stop feeding well on any day during the illness	yesno, Required<br>1 Yes<br>0 No<br><br>Custom alignment: RH

		_1] = '1' or [stirdor_ch_1] = '1' or [wheezing_ch_1] = '1')		
	251	[nasal_flaring_ch_1]<br><br>Show the field ONLY if: ([cough_ch_1] = '1') and ([difficult_breathing_ch_1] ='1' or [fast_breathing_ch_1] = '1' or [pasli_chalna_ch_1] = '1' or [stirdor_ch_1] = '1' or [wheezing_ch_1] = '1')	44. Did the child have nasal flaring during the illness	yesno, Required<br>1 Yes<br>0 No<br><br>Custom alignment: RH
	252	[central_cyanosis_ch_1]<br><br>Show the field ONLY if: ([cough_ch_1] = '1') and ([difficult_breathing_ch_1] ='1' or [fast_breathing_ch_1] = '1' or [pasli_chalna_ch_1] = '1' or [stirdor_ch_1] = '1' or [wheezing_ch_1] = '1')	45. Did the child have central cyanosis during the illness	yesno, Required<br>1 Yes<br>0 No<br><br>Custom alignment: RH
	253	[stridor_when_calm_ch_1]<br><br>Show the field ONLY if: ([cough_ch_1] = '1') and ([difficult_breathing_ch_1] ='1' or [fast_breathing_ch_1] = '1' or [pasli_chalna_ch_1] = '1' or [stirdor_ch_1] = '1' or [wheezing_ch_1] = '1')	46. Did the child have stridor when he was calm	yesno, Required<br>1 Yes<br>0 No<br><br>Custom alignment: RH
	254	[child_move_stumlate_ch_1]<br><br>Show the field ONLY if: ([cough_ch_1] = '1') and ([difficult_breathing_ch_1] ='1' or [fast_breathing_ch_1] = '1' or [pasli_chalna_ch_1] = '1' or [stirdor_ch_1] = '1' or [wheezing_ch_1] = '1')	47. Was the child moving only when stimulated on any day during the illness	yesno, Required<br>1 Yes<br>0 No<br><br>Custom alignment: RH
	255	[child_not_moving_ch_1]<br><br>Show the field ONLY if: ([cough_ch_1] = '1') and ([difficult_breathing_ch_1] ='1' or [fast_breathing_ch_1] = '1' or [pasli_chalna_ch_1] = '1' or [stirdor_ch_1] = '1' or [wheezing_ch_1] = '1')	48. Was the child not moving at all	yesno, Required<br>1 Yes<br>0 No<br><br>Custom alignment: RH
	256	[child_have_conv_fits_ch_1]<br><br>Show the field ONLY if: ([cough_ch_1] = '1') and ([difficult_breathing_ch_1] ='1' or [fast_breathing_ch_1] = '1' or [pasli_chalna_ch_1] = '1' or [stirdor_ch_1] = '1' or [wheezing_ch_1] = '1')	49. Did the child have convulsions or fits	yesno, Required<br>1 Yes<br>0 No<br><br>Custom alignment: RH

	257	[child_low_temp_ch_1]<br><br>Show the field ONLY if:<br>([cough_ch_1] = '1') and ([difficult_breathing_ch_1] ='1' or [fast_breathing_ch_1] = '1' or [pasli_chalna_ch_1] = '1' or [stirdor_ch_1] = '1' or [wheezing_ch_1] = '1')	50. Did the child have low body temperature	yesno, Required<br>1 Yes<br>0 No<br><br>Custom alignment: RH
	258	[other_last_ch_1]<br><br>Show the field ONLY if:<br>([cough_ch_1] = '1') and ([difficult_breathing_ch_1] ='1' or [fast_breathing_ch_1] = '1' or [pasli_chalna_ch_1] = '1' or [stirdor_ch_1] = '1' or [wheezing_ch_1] = '1')	Other	yesno, Required<br>1 Yes<br>0 No<br><br>Custom alignment: RH
	259	[specify_other_last_ch_1]<br><br>Show the field ONLY if:<br>[other_last_ch_1] = 1	Specify other	text, Required<br>Custom alignment: RH
	260	[child_recover_ot_ch_1]<br><br>Show the field ONLY if:<br>[pasli_chalna_ch_1] = '1' or [stirdor_ch_1] = '1' or [wheezing_ch_1] = '1' or [fast_breathing_ch_1] = '1' or [difficult_breathing_ch_1] = '1'	51. Did the child recover with treatment for pneumonia(fast breathing/difficult breathing/chest-indrawing)	yesno, Required<br>1 Yes<br>0 No
	261	[if_not_how_ot_ch_1]<br><br>Show the field ONLY if:<br>[child_recover_ot_ch_1] = '0'	52. If not how is the child now	radio, Required<br>1 Persistence of fast breathing<br>2 Persistence of chest indrawing<br>3 Child hospitalized during treatment<br>4 Developed SAE (Anaphylaxis reaction, severe diarrhea, severe rash all over body)<br>5 Child died
	262	[symptom_taking_med]<br><br>Show the field ONLY if:<br>[child_recover_ot_ch_1] = '0'	Does the child currently taking any medicines ?	yesno, Required<br>1 Yes<br>0 No
	263	[case_identified]<br><br>Show the field ONLY if:<br>[pasli_chalna_ch_1] = '1' or [stirdor_ch_1] = '1' or [wheezing_ch_1] = '1' or [fast_breathing_ch_1] = '1' or [difficult_breathing_ch_1] = '1'	CASE - IDENTIFIED	descriptive
	264	[did_you_seek_care_or_advic]<br><br>Show the field ONLY if:<br>[pasli_chalna_ch_1] = '1' or [stirdor_ch_1] = '1' or [wheezing_ch_1] = '1' or [fast_breathing_ch_1] = '1' or [difficult_breathing_ch_1] = '1'	Section Header: *Details of Care Seeking and Treatment Received*<br>53. Did you seek care or advice or treatment for Pneumonia symptom outside home?	yesno<br>1 Yes<br>0 No

	265	[care_or_advice_out_ch_1]<br><br>Show the field ONLY if:<br>[did_you_seek_care_or_advic] = '1'	For which illness :<br>(Tick the symptoms for which treatment is sought or advised)	checkbox, Required<br>6 | care_or_advice_out_ch_1___6 | Cough<br>7 | care_or_advice_out_ch_1___7 | Difficulty in b[illegible]<br>8 | care_or_advice_out_ch_1___8 | Fast breathin[illegible]<br>9 | care_or_advice_out_ch_1___9 | Pneumonia<br>10 | care_or_advice_out_ch_1___10 | Severe chest indrawing<br>11 | care_or_advice_out_ch_1___11 | Stridor (sou[illegible] breathing)<br>12 | care_or_advice_out_ch_1___12 | Wheezing<br>13 | care_or_advice_out_ch_1___13 | Diarrhea/das[illegible]<br>14 | care_or_advice_out_ch_1___14 | Dehydration/[illegible] kami<br>15 | care_or_advice_out_ch_1___15 | Sunken eyes<br>16 | care_or_advice_out_ch_1___16 | Restless/irrita[illegible]<br>17 | care_or_advice_out_ch_1___17 | Blood in stool<br>18 | care_or_advice_out_ch_1___18 | Fever 100.4° [illegible]<br>19 | care_or_advice_out_ch_1___19 | Stiff neck<br>20 | care_or_advice_out_ch_1___20 | Vomiting<br>21 | care_or_advice_out_ch_1___21 | Not able to breastfeed/d[illegible] feeding<br>22 | care_or_advice_out_ch_1___22 | Convulsions<br>23 | care_or_advice_out_ch_1___23 | Lethargic<br>24 | care_or_advice_out_ch_1___24 | Unconscious<br>25 | care_or_advice_out_ch_1___25 | Eye discharge<br>26 | care_or_advice_out_ch_1___26 | Ear discharge<br>27 | care_or_advice_out_ch_1___27 | Skin infection<br>28 | care_or_advice_out_ch_1___28 | Bulging fonta[illegible]<br>29 | care_or_advice_out_ch_1___29 | Cold/running<br>30 | care_or_advice_out_ch_1___30 | Very weak/malnut[illegible]<br>31 | care_or_advice_out_ch_1___31 | Measles<br>32 | care_or_advice_out_ch_1___32 | Mouth ulcer<br>33 | care_or_advice_out_ch_1___33 | Umbilicus re[illegible] draining pus<br>34 | care_or_advice_out_ch_1___34 | Skin pustules<br>35 | care_or_advice_out_ch_1___35 | Yellow soles<br>36 | care_or_advice_out_ch_1___36 | Low body temperature/ Hypothermia
	266	[other_ill_care_trt_ch_1]<br><br>Show the field ONLY if:<br>[did_you_seek_care_or_advic] = '1'	Any other illness different then above?	yesno, Required<br>1 | Yes<br>0 | No
	267	[sp_oth_ill_care]	Specify other	text

		Show the field ONLY if:<br>[other_ill_care_trt_ch_1] = '1'		
	268	[asha]<br><br>Show the field ONLY if:<br>[did_you_seek_care_or_advic] = '1'	Section Header: *54. Where did you seek care or advise or treatment outside home?. Fill the order in which the treatment was sought.*<br>ASHA	checkbox<br>1 | asha___1 | 1<br>2 | asha___2 | 2<br>3 | asha___3 | 3<br>4 | asha___4 | 4<br>5 | asha___5 | 5<br>6 | asha___6 | 6
	269	[anm]<br><br>Show the field ONLY if:<br>[did_you_seek_care_or_advic] = '1'	ANM	checkbox<br>1 | anm___1 | 1<br>2 | anm___2 | 2<br>3 | anm___3 | 3<br>4 | anm___4 | 4<br>5 | anm___5 | 5<br>6 | anm___6 | 6
	270	[aww]<br><br>Show the field ONLY if:<br>[did_you_seek_care_or_advic] = '1'	AWW	checkbox<br>1 | aww___1 | 1<br>2 | aww___2 | 2<br>3 | aww___3 | 3<br>4 | aww___4 | 4<br>5 | aww___5 | 5<br>6 | aww___6 | 6
	271	[sub_center]<br><br>Show the field ONLY if:<br>[did_you_seek_care_or_advic] = '1'	Sub center	checkbox<br>1 | sub_center___1 | 1<br>2 | sub_center___2 | 2<br>3 | sub_center___3 | 3<br>4 | sub_center___4 | 4<br>5 | sub_center___5 | 5<br>6 | sub_center___6 | 6
	272	[health_and_wellness_center]<br><br>Show the field ONLY if:<br>[did_you_seek_care_or_advic] = '1'	Health and wellness center	checkbox<br>1 | health_and_wellness_center___1 | 1<br>2 | health_and_wellness_center___2 | 2<br>3 | health_and_wellness_center___3 | 3<br>4 | health_and_wellness_center___4 | 4<br>5 | health_and_wellness_center___5 | 5<br>6 | health_and_wellness_center___6 | 6
	273	[primary_health_centre]<br><br>Show the field ONLY if:<br>[did_you_seek_care_or_advic] = '1'	Primary Health Centre	checkbox<br>1 | primary_health_centre___1 | 1<br>2 | primary_health_centre___2 | 2<br>3 | primary_health_centre___3 | 3<br>4 | primary_health_centre___4 | 4<br>5 | primary_health_centre___5 | 5

				6 primary_health_centre___6 6
	274	[community_health_center]<br>Show the field ONLY if:<br>[did_you_seek_care_or_advic] = '1'	Community health center	checkbox<br>1 community_health_center___1 1<br>2 community_health_center___2 2<br>3 community_health_center___3 3<br>4 community_health_center___4 4<br>5 community_health_center___5 5<br>6 community_health_center___6 6
	275	[district_government_hospit]<br>Show the field ONLY if:<br>[did_you_seek_care_or_advic] = '1'	District/Government hospital/ Sub district hospital	checkbox<br>1 district_government_hospit___1 1<br>2 district_government_hospit___2 2<br>3 district_government_hospit___3 3<br>4 district_government_hospit___4 4<br>5 district_government_hospit___5 5<br>6 district_government_hospit___6 6
	276	[chemist_shop]<br>Show the field ONLY if:<br>[did_you_seek_care_or_advic] = '1'	Chemist shop	checkbox<br>1 chemist_shop___1 1<br>2 chemist_shop___2 2<br>3 chemist_shop___3 3<br>4 chemist_shop___4 4<br>5 chemist_shop___5 5<br>6 chemist_shop___6 6
	277	[private_mbbs_specilization]<br>Show the field ONLY if:<br>[did_you_seek_care_or_advic] = '1'	Private practitioner (MBBS + specialization)	checkbox<br>1 private_mbbs_specilization___1 1<br>2 private_mbbs_specilization___2 2<br>3 private_mbbs_specilization___3 3<br>4 private_mbbs_specilization___4 4<br>5 private_mbbs_specilization___5 5<br>6 private_mbbs_specilization___6 6
	278	[private_practitioner_mbbs]<br>Show the field ONLY if:<br>[did_you_seek_care_or_advic] = '1'	Private practitioner (Medical Doctor, MBBS)	checkbox<br>1 private_practitioner_mbbs___1 1<br>2 private_practitioner_mbbs___2 2<br>3 private_practitioner_mbbs___3 3<br>4 private_practitioner_mbbs___4 4<br>5 private_practitioner_mbbs___5 5<br>6 private_practitioner_mbbs___6 6
	279	[homeopathy_bhms]<br>Show the field ONLY if:<br>[did_you_seek_care_or_advic] = '1'	Private practitioner (Homeopathy, BHMS)	checkbox<br>1 homeopathy_bhms___1 1<br>2 homeopathy_bhms___2 2<br>3 homeopathy_bhms___3 3<br>4 homeopathy_bhms___4 4<br>5 homeopathy_bhms___5 5<br>6 homeopathy_bhms___6 6

	280	[ayurveda_bams]<br>Show the field ONLY if: [did_you_seek_care_or_advic] = '1'	Private practitioner (Ayurveda, BAMS)	checkbox<br>1 ayurveda_bams___1 1<br>2 ayurveda_bams___2 2<br>3 ayurveda_bams___3 3<br>4 ayurveda_bams___4 4<br>5 ayurveda_bams___5 5<br>6 ayurveda_bams___6 6
	281	[rmp]<br>Show the field ONLY if: [did_you_seek_care_or_advic] = '1'	Private practitioner (RMP)	checkbox<br>1 rmp___1 1<br>2 rmp___2 2<br>3 rmp___3 3<br>4 rmp___4 4<br>5 rmp___5 5<br>6 rmp___6 6
	282	[degree_unknown]<br>Show the field ONLY if: [did_you_seek_care_or_advic] = '1'	Private practitioner (No degree/ degree not known)	checkbox<br>1 degree_unknown___1 1<br>2 degree_unknown___2 2<br>3 degree_unknown___3 3<br>4 degree_unknown___4 4<br>5 degree_unknown___5 5<br>6 degree_unknown___6 6
	283	[private_other]<br>Show the field ONLY if: [did_you_seek_care_or_advic] = '1'	Private practitioner (Others, specify)	checkbox<br>1 private_other___1 1<br>2 private_other___2 2<br>3 private_other___3 3<br>4 private_other___4 4<br>5 private_other___5 5<br>6 private_other___6 6
	284	[private_nursing_home_hospi]<br>Show the field ONLY if: [did_you_seek_care_or_advic] = '1'	Private nursing home/hospital	checkbox<br>1 private_nursing_home_hospi___1 1<br>2 private_nursing_home_hospi___2 2<br>3 private_nursing_home_hospi___3 3<br>4 private_nursing_home_hospi___4 4<br>5 private_nursing_home_hospi___5 5<br>6 private_nursing_home_hospi___6 6
	285	[not_sought]<br>Show the field ONLY if: [did_you_seek_care_or_advic] = '1'	Not sought	checkbox<br>1 not_sought___1 1<br>2 not_sought___2 2<br>3 not_sought___3 3<br>4 not_sought___4 4<br>5 not_sought___5 5<br>6 not_sought___6 6
	286	[yes_how_much_c_t_ch_1]	56. If yes, how much time after identification of the illness did you seek care. Days {identification_day}	descriptive

		Show the field ONLY if: [did_you_seek_care_or_advic] = '1'	Hours {identification_hours}	
	287	[identification_hours]	Hours	text (number, Min: 0, Max: 23)
	288	[identification_day]	Days	text (number, Min: 0, Max: 40)
	289	[cough_syrup_cst_ch_1]<br><br>Show the field ONLY if: [did_you_seek_care_or_advic] = '1'	Section Header: *57. What treatment was given by any source to child for the illness (check the medicines if possible. If not possible, record what the caregiver says).*<br><br>Cough syrup	radio (Matrix)<br>3 Seen<br>4 Reported
	290	[gentamycin_cst_ch_1]<br><br>Show the field ONLY if: [did_you_seek_care_or_advic] = '1'	Gentamycin	radio (Matrix)<br>3 Seen<br>4 Reported
	291	[amoxycillin_cst_ch_1]<br><br>Show the field ONLY if: [did_you_seek_care_or_advic] = '1'	Amoxycillin	radio (Matrix)<br>3 Seen<br>4 Reported
	292	[antibiotic_cst_ch_1]<br><br>Show the field ONLY if: [did_you_seek_care_or_advic] = '1'	Antibiotic	radio (Matrix)<br>3 Seen<br>4 Reported
	293	[unknown_tablet_cst_ch_1]<br><br>Show the field ONLY if: [did_you_seek_care_or_advic] = '1'	Unknown tablet	radio (Matrix)<br>3 Seen<br>4 Reported
	294	[unknown_syrup_cst_ch_1]<br><br>Show the field ONLY if: [did_you_seek_care_or_advic] = '1'	Unknown syrup	radio (Matrix)<br>3 Seen<br>4 Reported
	295	[unknown_powder_cst_ch_1]<br><br>Show the field ONLY if: [did_you_seek_care_or_advic] = '1'	Unknown powder	radio (Matrix)<br>3 Seen<br>4 Reported
	296	[injection_cst_ch_1]<br><br>Show the field ONLY if: [did_you_seek_care_or_advic] = '1'	Injection	radio (Matrix)<br>3 Seen<br>4 Reported
	297	[injection_unknown_cst_ch_1]<br><br>Show the field ONLY if: [did_you_seek_care_or_advic] = '1'	Injection unknown	radio (Matrix)<br>3 Seen<br>4 Reported
	298	[inhalation_cst_ch_1]<br><br>Show the field ONLY if: [did_you_seek_care_or_advic] = '1'	Inhalation	radio (Matrix)<br>3 Seen<br>4 Reported
	299	[nebulization_cst_ch_1]<br><br>Show the field ONLY if: [did_you_seek_care_or_advic] = '1'	Nebulization	radio (Matrix)<br>3 Seen<br>4 Reported

	300	[iv_fluids_cst_ch_1]<br><br>Show the field ONLY if:<br>[did_you_seek_care_or_advic] = '1'	IV Fluids	radio (Matrix)<br>3 Seen<br>4 Reported
	301	[paracetamol_cst_ch_1]<br><br>Show the field ONLY if:<br>[did_you_seek_care_or_advic] = '1'	Paracetamol	radio (Matrix)<br>3 Seen<br>4 Reported
	302	[oxygen_therapy_cst_ch_1]<br><br>Show the field ONLY if:<br>[did_you_seek_care_or_advic] = '1'	Oxygen therapy	radio (Matrix)<br>3 Seen<br>4 Reported
	303	[bronchodilators_cst_ch_1]<br><br>Show the field ONLY if:<br>[did_you_seek_care_or_advic] = '1'	Bronchodilators	radio (Matrix)<br>3 Seen<br>4 Reported
	304	[zinc_cst_ch_1]<br><br>Show the field ONLY if:<br>[did_you_seek_care_or_advic] = '1'	Zinc	radio (Matrix)<br>3 Seen<br>4 Reported
	305	[ors_cst_ch_1]<br><br>Show the field ONLY if:<br>[did_you_seek_care_or_advic] = '1'	ORS	radio (Matrix)<br>3 Seen<br>4 Reported
	306	[other_med_cst_ch_1]<br><br>Show the field ONLY if:<br>[did_you_seek_care_or_advic] = '1'	Other	radio (Matrix)<br>3 Seen<br>4 Reported
	307	[sp_oth_med_cst_ch_1]<br><br>Show the field ONLY if:<br>[other_med_cst_ch_1] > 0	Specify other	text, Required
	308	[amox_advise_who_cst_ch_1]<br><br>Show the field ONLY if:<br>[amoxycillin_cst_ch_1] = '3' or [amoxycillin_cst_ch_1] = '4'	58. Who advised Amoxycillin ?	radio, Required<br>1 ASHA<br>2 ANM<br>3 AWW<br>4 Sub center<br>5 Health and wellness center<br>6 Primary Health Centre<br>7 Community health center<br>8 District/Government hospital/ Sub district hospital<br>9 Chemist shop<br>10 Private practitioner (MBBS + specialization)<br>11 Private practitioner (Medical Doctor, MBBS)<br>12 Private practitioner (Homeopathy, BHMS)<br>13 Private practitioner (Ayurveda, BAMS)

				14 Private practitioner (RMP)<br>15 Private practitioner (No degree/ degree not known)<br>16 Private practitioner (Others, specify)<br>17 Private nursing home/hospital<br>99 Other
	309	[amox_oth_advise_cst_c_1]<br>Show the field ONLY if: [amox_advise_who_cst_ch_1] = 99	Other Specify	text, Required
	310	[amox_receive_day]<br>Show the field ONLY if: [amoxycillin_cst_ch_1] = '3' or [amoxycillin_cst_ch_1] = '4'	If Amoxycillin advised, how many days did the child receive Amoxycillin	text (number)
	311	[amox_no_time]<br>Show the field ONLY if: [amoxycillin_cst_ch_1] = '3' or [amoxycillin_cst_ch_1] = '4'	If amoxicillin advised, number of times in a day it was advised for	text (number, Min: 1, Max: 12)
	312	[amox_given]<br>Show the field ONLY if: [amoxycillin_cst_ch_1] = '3' or [amoxycillin_cst_ch_1] = '4'	How many days did you give amoxicillin (Enter 0 if not given).	text (number, Min: 0, Max: 12)
	313	[amox_given_times]<br>Show the field ONLY if: [amoxycillin_cst_ch_1] = '3' or [amoxycillin_cst_ch_1] = '4'	How many times in a day did you give amoxicillin to your infant	text (number, Min: 1)
	314	[genta_advise_who_cst_ch_1]<br>Show the field ONLY if: [gentamycin_cst_ch_1] = '3' or [gentamycin_cst_ch_1] = '4'	59. Who advised Gentamycin?	radio, Required<br>1 ASHA<br>2 ANM<br>3 AWW<br>4 Sub center<br>5 Health and wellness center<br>6 Primary Health Centre<br>7 Community health center<br>8 District/Government hospital/ Sub district hospital<br>9 Chemist shop<br>10 Private practitioner (MBBS + specialization)<br>11 Private practitioner (Medical Doctor, MBBS)<br>12 Private practitioner (Homeopathy, BHMS)<br>13 Private practitioner (Ayurveda, BAMS)<br>14 Private practitioner (RMP)

				15 Private practitioner (No degree/ degree not known)<br>16 Private practitioner (Others, specify)<br>17 Private nursing home/hospital<br>99 Other
	315	[genta_other_advise]<br><br>Show the field ONLY if:<br>[genta_advise_who_cst_ch_1] = '99'	Other Specify	text, Required
	316	[amoxy_genta_table]<br><br>Show the field ONLY if:<br>[gentamycin_cst_ch_1] = '3' or [gentamycin_cst_ch_1] = '4' or [amoxycillin_cst_ch_1] = '3' or [amoxycillin_cst_ch_1] ='4'	Advise Advise followed No of Days Dosage per day No of Days Dosage per day Amoxycillin {amox_receive_day} {amox_no_time} {amox_given} {amox_given_times} Gentamycin {genta_advise_day} {genta_no_times} {gentamycin_days} {genta_given_times}	descriptive
	317	[genta_advise_day]<br><br>Show the field ONLY if:<br>[gentamycin_cst_ch_1] = '3' or [gentamycin_cst_ch_1] = '4'	If Gentamycin advised, how many days did the child receive Gentamycin?	text, Required
	318	[genta_no_times]<br><br>Show the field ONLY if:<br>[gentamycin_cst_ch_1] = '3' or [gentamycin_cst_ch_1] = '4'	If Gentamycin advised, number of times in a day it was advised for	text (number, Min: 1), Required
	319	[gentamycin_days]<br><br>Show the field ONLY if:<br>[gentamycin_cst_ch_1] = '3' or [gentamycin_cst_ch_1] = '4'	How many days did you give Gentamycin (Enter 0 if not given)	text (number), Required
	320	[genta_given_times]<br><br>Show the field ONLY if:<br>[gentamycin_cst_ch_1] = '3' or [gentamycin_cst_ch_1] = '4'	How many times in a day did you give Gentamycin to your infant ?	text, Required
	321	[amox_genta_conti_cst_ch_1]<br><br>Show the field ONLY if:<br>[gentamycin_cst_ch_1] = '3' or [gentamycin_cst_ch_1] = '4' or [amoxycillin_cst_ch_1] = '3' or [amoxycillin_cst_ch_1] = '4'	60. Did you continue medication (amoxicillin/gentamycin) as per recommendation given by the health care providers	yesno, Required<br>1 Yes<br>0 No
	322	[home_treat_given_cst_ch_1]<br><br>Show the field ONLY if:<br>[did_you_seek_care_or_advic] ='1'	61. Was any home treatment given for the illness	yesno, Required<br>1 Yes<br>0 No

	323	[yes_home_treat_cst_ch_1]<br>Show the field ONLY if:<br>[home_treat_given_cst_ch_1] = '1'	If yes what	radio, Required<br>1 Hot drink with ginger or lemon or tulsi leaves with or without sugar<br>2 Hot drink with ginger or lemon or mint with or without sugar<br>3 Tea with saunf or elaichi or ginger with or without sugar<br>4 Others
	324	[home_treat_oth_cst_ch_1]<br>Show the field ONLY if:<br>[yes_home_treat_cst_ch_1] = 4	Specify other	text, Required
	325	[no_treat_sought_cst_ch_1]<br>Show the field ONLY if:<br>[did_you_seek_care_or_advic] ='0'	62. If treatment was never sought, for cough/fast breathing/difficulty in breathing/fever, ask.<br>Why did you not seek treatment (spontaneous)	radio, Required<br>1 Do not know where to go for care seeking<br>2 Do not know whom to contact<br>3 Not aware about the availability of treatment for this illness<br>4 Did not realize that treatment was required for this illness<br>5 Not aware that the medicines were free of cost at the government facilities<br>6 Others
	326	[oth_treat_sought_cst_ch_1]<br>Show the field ONLY if:<br>[no_treat_sought_cst_ch_1] = 6	Specify, other	text, Required
	327	[was_reffered_cst_ch_1]<br>Show the field ONLY if:<br>[did_you_seek_care_or_advic] = '1'	Section Header: *Referral Information*<br>63. Was the child referred for any illness in the previous month?	yesno, Required<br>1 Yes<br>0 No
	328	[yes_which_illness_cst_ch_1]<br>Show the field ONLY if:<br>[was_reffered_cst_ch_1] = '1'	If yes, for which illness	checkbox, Required<br>1 yes_which_illness_cst_ch_1___1 Cough<br>2 yes_which_illness_cst_ch_1___2 Difficulty in<br>3 yes_which_illness_cst_ch_1___3 Fast breath<br>4 yes_which_illness_cst_ch_1___4 Fast breath < 90%<br>5 yes_which_illness_cst_ch_1___5 Pneumonia<br>6 yes_which_illness_cst_ch_1___6 Diarrhea<br>7 yes_which_illness_cst_ch_1___7 Fever 100.4<br>8 yes_which_illness_cst_ch_1___8 Severe ches<br>9 yes_which_illness_cst_ch_1___9 Unable to fe feeding wel<br>10 yes_which_illness_cst_ch_1___10 Low body temperatur<br>11 yes_which_illness_cst_ch_1___11 Umbilicus r pus<br>12 yes_which_illness_cst_ch_1___12 Skin pustule

				14 | yes_which_illness_cst_ch_1___14 | Others
	329	[yes_oth_illness_cst_ch_1]<br><br>Show the field ONLY if: ([yes_which_illness_cst_ch_1(14)] = '1') and ([yes_which_illness_cst_ch_1(13)] = '1' or [yes_which_illness_cst_ch_1(12)] = '1' or [yes_which_illness_cst_ch_1(11)] = '1' or [yes_which_illness_cst_ch_1(10)] = '1' or [yes_which_illness_cst_ch_1(9)] = '1' or [yes_which_illness_cst_ch_1(8)] = '1' or [yes_which_illness_cst_ch_1(7)] = '1' or [yes_which_illness_cst_ch_1(6)] = '1' or [yes_which_illness_cst_ch_1(5)] = '1' or [yes_which_illness_cst_ch_1(4)] = '1' or [yes_which_illness_cst_ch_1(3)] = '1' or [yes_which_illness_cst_ch_1(2)] = '1' or [yes_which_illness_cst_ch_1(1)] = '1')	Specify, Other	text, Required
	330	[who_refer_cst_ch_1]<br><br>Show the field ONLY if: [was_reffered_cst_ch_1] = '1'	64. Who referred the child	radio, Required<br>1 ASHA<br>2 ANM<br>3 HWC<br>4 PHC<br>5 CHC<br>6 Government hospital<br>7 Private hospital<br>8 Private clinic<br>9 Other
	331	[other_refer_cst_ch_1]<br><br>Show the field ONLY if: [who_refer_cst_ch_1] = 9	Specify other	text, Required
	332	[where_oth_refer_cst_ch_1]<br><br>Show the field ONLY if: [who_refer_cst_ch_1] = 9	65. Where was the child referred by other?	radio, Required<br>1 ANM<br>2 HWC<br>3 PHC<br>4 CHC<br>5 Government hospital<br>6 Private hospital<br>7 Private clinic<br>9 Other
	333	[comply_referral_cst_ch1]<br><br>Show the field ONLY if: [was_reffered_cst_ch_1] = '1'	66. Did you comply with referral recommendation made by health care providers?	yesno, Required<br>1 Yes<br>0 No

	334	[comply_reffer_no_reason]<br><br>Show the field ONLY if: [comply_referral_cst_ch1] = '0'	If no, please specify	text
	335	[refer_last_month_cst_ch_1]<br><br>Show the field ONLY if: [was_reffered_cst_ch_1] = '1'	67. In case your child was referred for any illness in the last 1 month and you took him/her to the referred source, what happened at the referral facility	radio, Required<br>1 They did not entertain us<br>2 They did not examine the child<br>3 They sent us back without any treatment<br>4 They examined the child and wrote/dispensed some medicines and sent us back<br>5 They did not examine the child but wrote some medicines to buy from outside<br>6 They admitted the child<br>9 Others
	336	[refer_last_oth_cst_ch_1]<br><br>Show the field ONLY if: [refer_last_month_cst_ch_1] = 9	Specify Other	text, Required
	337	[visit_refer_hos_cst_ch_1]<br><br>Show the field ONLY if: [was_reffered_cst_ch_1] = '1'	68. In case your child was referred for any illness in last 1 month and you took him/her to the source of referral, how long after you were advised, did you take the child (write in hours).<br>If you did not take the child for referral, fill '999'	text (number), Required
	338	[taken_to_hwc_phc_cst_ch_1]<br><br>Show the field ONLY if: [visit_refer_hos_cst_ch_1] > 0	69. If taken to the HWC/PHC/CHC/GH/private hospital for pneumonia, fast breathing, and chest in-drawing was the child given any medicine ?	radio, Required<br>1 Gentamycine<br>2 Amoxicillin<br>3 No medicine given
	339	[yes_inj_days_time_cst_ch_1]<br><br>Show the field ONLY if: [taken_to_hwc_phc_cst_ch_1] = '1' or [taken_to_hwc_phc_cst_ch_1] = '2'	70. If yes, for how many days was medicine given?	text (number), Required
	340	[yes_inj_time_cst_ch_1]<br><br>Show the field ONLY if: [taken_to_hwc_phc_cst_ch_1] = '1' or [taken_to_hwc_phc_cst_ch_1] = '2'	71. The number of times medicine given (per day)	text (number), Required
	341	[refer_no_reason_cst_ch_1]<br><br>Show the field ONLY if: [visit_refer_hos_cst_ch_1] = '999'	72. If you did not take the child for referral, what were the reason(s). (Select all that apply)	checkbox, Required<br>1 refer_no_reason_cst_ch_1___1 No transportatio<br>2 refer_no_reason_cst_ch_1___2 Nobody to accompany<br>3 refer_no_reason_cst_ch_1___3 Government facilities wer closed<br>4 refer_no_reason_cst_ch_1___4 Medicines ar not available

		at govt. facilities
5	refer_no_reason_cst_ch_1___5	Doctors are not available at govt. facilities
6	refer_no_reason_cst_ch_1___6	Facilities are not appropriate govt. centers
7	refer_no_reason_cst_ch_1___7	Private facilities are very expensive
8	refer_no_reason_cst_ch_1___8	Did not feel child was ill
9	refer_no_reason_cst_ch_1___9	Did not feel that the chil[illegible] was ill enoug[illegible] to require referral
10	refer_no_reason_cst_ch_1___10	Family members sa[illegible] that referral not required
11	refer_no_reason_cst_ch_1___11	Did not kno[illegible] where to go for referral
12	refer_no_reason_cst_ch_1___12	Referral places were far away fro[illegible] residence
13	refer_no_reason_cst_ch_1___13	Did not have money for transportatio[illegible]
14	refer_no_reason_cst_ch_1___14	Did not have money to pa[illegible] for the hospital cost
15	refer_no_reason_cst_ch_1___15	Others

	342	[refer_oth_reason_cst_ch_1]<br>Show the field ONLY if: [refer_no_reason_cst_ch_1(15)] = '1'	Specify other reason	text, Required
	343	[care_equip_cst_ch_1]<br>Show the field ONLY if: [visit_refer_hos_cst_ch_1] > 0	73. If care was sought for any illness, what equipment were used to assess the child:	checkbox, Required

1	care_equip_cst_ch_1___1	Digital Timer
2	care_equip_cst_ch_1___2	Digital Thermometer
3	care_equip_cst_ch_1___3	MUAC Tape
4	care_equip_cst_ch_1___4	Weighing Scale
5	care_equip_cst_ch_1___5	Pulse Oximeter
6	care_equip_cst_ch_1___6	Stethoscope
7	care_equip_cst_ch_1___7	Other

	344	[care_equip_oth_cst_ch_1]<br>Show the field ONLY if:<br>[care_equip_cst_ch_1(7)] = '1'	specify other	text, Required
	345	[fast_b_oxy_lvl_cst_ch_1]<br>Show the field ONLY if:<br>[visit_refer_hos_cst_ch_1] > 0	74. If child had fast breathing, was oxygen saturation level checked	yesno, Required<br>1 Yes<br>0 No
	346	[oxy_lvl_low_cst_ch_1]<br>Show the field ONLY if:<br>[fast_b_oxy_lvl_cst_ch_1] = '1'	75. Was the oxygen saturation level low (SpO2 < 90%)	yesno, Required<br>1 Yes<br>0 No
	347	[visit_hosp_csc_ch_1]<br>Show the field ONLY if:<br>[did_you_seek_care_or_advic] = '1'	Section Header: *Out-patient Care Expenditures*<br>77. How many times has your child visited a health facility for out-patient care due to any illness in the last 1 month	text (number, Min: 0), Required
	348	[drugs_cost_csc_v_1]<br>Show the field ONLY if:<br>[did_you_seek_care_or_advic] = '1'	Section Header: *How much did you spend on out-patient care for treatment of your child's illness ?*<br>78. Drugs	text (number, Max: 100000), Required
	349	[invest_cost_csc_v_1]<br>Show the field ONLY if:<br>[did_you_seek_care_or_advic] = '1'	79. Investigations/Tests	text (number, Max: 100000), Required
	350	[consult_cost_csc_v_1]<br>Show the field ONLY if:<br>[did_you_seek_care_or_advic] = '1'	80. Consultation Fee	text (number, Min: 0, Max: 100000), Required
	351	[transport_cost_csc_v_1]<br>Show the field ONLY if:<br>[did_you_seek_care_or_advic] = '1'	81. Transportation to and from health facility	text (number, Min: 0, Max: 100000), Required
	352	[extra_food_cost_csc_v_1]<br>Show the field ONLY if:<br>[did_you_seek_care_or_advic] = '1'	82. Extra food cost (any special food bought for the infant for example any milk or other food, fluid and food bought for caregivers who accompanied the infant to the treatment provider)	text (number, Min: 0, Max: 100000), Required
	353	[additional_cost_csc_v_1]<br>Show the field ONLY if:<br>[did_you_seek_care_or_advic] = '1'	83. Additional expenses for care giver	text (number, Min: 0, Max: 100000), Required
	354	[wages_lost_csc_v_1]<br>Show the field ONLY if:<br>[did_you_seek_care_or_advic] = '1'	84. Wages lost	text (number, Min: 0, Max: 100000), Required
	355	[trad_heal_cost_csc_v_1]<br>Show the field ONLY if:<br>[did_you_seek_care_or_advic] = '1'	85. If traditional healer visited for this episode, amount paid (total expenditure)	text (number, Min: 0, Max: 100000), Required

	356	[oth_cost_csc_v_1]<br><br>Show the field ONLY if:<br>[did_you_seek_care_or_advic] = '1'	86. Other Expense	text (number, Min: 0, Max: 100000), Required
	357	[total_expendi_csc_v_1]<br><br>Show the field ONLY if:<br>[did_you_seek_care_or_advic] = '1'	Total Expenditure	calc, Required<br>Calculation: [drugs_cost_csc_v_1] + [invest_cost_csc_v_1] + [consult_cost_csc_v_1] + [transport_cost_csc_v_1] + [extra_food_cost_csc_v_1] + [additional_cost_csc_v_1] + [wages_lost_csc_v_1] + [trad_heal_cost_csc_v_1] + [oth_cost_csc_v_1]
	358	[illness_1_csc_v_1]<br><br>Show the field ONLY if:<br>[did_you_seek_care_or_advic] = '1'	Section Header: *Home Treatment Expenditure*<br><br>87. Illness 1	text (number), Required
	359	[illness_2_csc_v_1]<br><br>Show the field ONLY if:<br>[did_you_seek_care_or_advic] = '1'	88. Illness 2	text (number), Required
	360	[illness_3_css_v_1]<br><br>Show the field ONLY if:<br>[did_you_seek_care_or_advic] = '1'	89. Illness 3	text (number), Required
	361	[t_cost_home_treat_csc_v_1]<br><br>Show the field ONLY if:<br>[did_you_seek_care_or_advic] = '1'	90. Total Amount Spent on home treatment, if given, for any illness	calc<br>Calculation: [illness_1_csc_v_1] + [illness_2_csc_v_1] + [illness_3_css_v_1]
	362	[child_complete]	Section Header: *Form Status*<br><br>Complete?	dropdown<br>0 Incomplete<br>1 Unverified<br>2 Complete
**Instrument: Hospitalization (hospitalization)**				
	363	[is_the_child_hospitalized]	Section Header: *Hospitalization (fill separately for each hospitalization) Fill if the child was hospitalized in the last 3 months.*<br><br>Is the child hospitalized in the previous 3 months?	yesno, Required<br>1 Yes<br>0 No
	364	[no_times_hosp_hi_ch_1]<br><br>Show the field ONLY if:<br>[is_the_child_hospitalized] = '1'	Number of times hospitalized	text (number), Required
	365	[name_child_hospitalized]<br><br>Show the field ONLY if:<br>[is_the_child_hospitalized] = '1'	1. Name	text, Required
	366	[hosp_father_name]	2. Father Name	text, Required
	367	[reasons_hosp_hi_ch_1]<br><br>Show the field ONLY if:<br>[is_the_child_hospitalized] = '1'	3. What were the reasons for hospitalization ?	radio, Required<br>3 Spontaneous<br>4 Prompted
	368	[select_reasons_hi_ch_1]	4. Select reasons for hospitalization	checkbox, Required

		Show the field ONLY if:<br>[is_the_child_hospitalized] = '1'		

6	select_reasons_hi_ch_1___6	Cough
7	select_reasons_hi_ch_1___7	Difficulty in bre
8	select_reasons_hi_ch_1___8	Fast breathing
9	select_reasons_hi_ch_1___9	Pneumonia
10	select_reasons_hi_ch_1___10	Severe chest indrawing
11	select_reasons_hi_ch_1___11	Stridor (sound i breathing)
12	select_reasons_hi_ch_1___12	Wheezing
13	select_reasons_hi_ch_1___13	Diarrhea/dast
14	select_reasons_hi_ch_1___14	Dehydration/pa kami
15	select_reasons_hi_ch_1___15	Sunken eyes
16	select_reasons_hi_ch_1___16	Restless/irritab
17	select_reasons_hi_ch_1___17	Blood in stool
18	select_reasons_hi_ch_1___18	Fever 100.4? F (C)
19	select_reasons_hi_ch_1___19	Stiff neck
20	select_reasons_hi_ch_1___20	Vomiting
21	select_reasons_hi_ch_1___21	Not able to breastfeed/drir feeding
22	select_reasons_hi_ch_1___22	Convulsions
23	select_reasons_hi_ch_1___23	Lethargic
24	select_reasons_hi_ch_1___24	Unconscious
25	select_reasons_hi_ch_1___25	Eye discharge
26	select_reasons_hi_ch_1___26	Ear discharge
27	select_reasons_hi_ch_1___27	Skin infections
28	select_reasons_hi_ch_1___28	Bulging fontane
29	select_reasons_hi_ch_1___29	Cold/running n
30	select_reasons_hi_ch_1___30	Very weak/malnutrit
31	select_reasons_hi_ch_1___31	Measles
32	select_reasons_hi_ch_1___32	Mouth ulcer
33	select_reasons_hi_ch_1___33	Umbilicus red c draining pus
34	select_reasons_hi_ch_1___34	Skin pustules
35	select_reasons_hi_ch_1___35	Yellow soles
37	select_reasons_hi_ch_1___37	Low body temperature/ Hypothermia

	369	[type_of_hospital_hi_ch_1]<br>Show the field ONLY if:<br>[is_the_child_hospitalized] = '1'	5. Type of Hospital	radio, Required<br>1 Government<br>2 Private<br>3 Other
	370	[remember_date_yes_no]	6. Did you remember the date of admission and date of discharge?	yesno, Required

		Show the field ONLY if: [is_the_child_hospitalized] = '1'		1 Yes<br>0 No
	371	[doa_hi_ch_1]<br>Show the field ONLY if: [remember_date_yes_no] = '1'	7. What was the date of admission	text (date_dmy, Max: now)
	372	[dod_hi_ch_1]<br>Show the field ONLY if: [remember_date_yes_no] = '1'	8. What was the date of discharge	text (date_dmy, Max: now)
	373	[no_of_days_hosp_hi_ch_1]<br>Show the field ONLY if: [remember_date_yes_no] = '0'	9. If unable to recall the dates, number of days of hospitalization	text (number), Required
	374	[document_hi_ch_1]<br>Show the field ONLY if: [is_the_child_hospitalized] = '1'	10. If documents available copy the diagnosis	file
	375	[admis_cost_csc_hv_1]<br>Show the field ONLY if: [is_the_child_hospitalized] = '1'	Section Header: *For the each hospitalization, how much did you spend on each of the following items during the total stay?*<br>11. Admission Fee	text (number), Required
	376	[bed_cost_csc_hv_1]<br>Show the field ONLY if: [is_the_child_hospitalized] = '1'	12. Hospital bed	text (number), Required
	377	[drugs_cost_csc_hv_1]<br>Show the field ONLY if: [is_the_child_hospitalized] = '1'	13. Drugs	text (number), Required
	378	[consult_cost_csc_hv_1]<br>Show the field ONLY if: [is_the_child_hospitalized] = '1'	14. Consultation fee	text (number), Required
	379	[invest_cost_csc_hv_1]<br>Show the field ONLY if: [is_the_child_hospitalized] = '1'	15. Investigations/tests	text (number), Required
	380	[food_cost_csc_hv_1]<br>Show the field ONLY if: [is_the_child_hospitalized] = '1'	16. Food (any special food bought for the infant for example any milk or other food, fluid and food bought for caregivers who stayed with the infant in the hospital)	text (number), Required
	381	[transport_cost_csc_hv_1]<br>Show the field ONLY if: [is_the_child_hospitalized] = '1'	17. Transportation to and from the hospital	text (number), Required
	382	[wages_lost_csc_hv_1]<br>Show the field ONLY if:	18. Wages lost	text (number), Required

		[is_the_child_hospitalized] = '1'		
	383	[additonal_cost_csc_hv_1]<br>Show the field ONLY if: [is_the_child_hospitalized] = '1'	19. Additional expenses for the care giver.	text (number), Required
	384	[other_cost_csc_hv_1]<br>Show the field ONLY if: [is_the_child_hospitalized] = '1'	Other	text (number), Required
	385	[total_expenditure]<br>Show the field ONLY if: [is_the_child_hospitalized] = '1'	Total expenditure	calc, Required<br>Calculation: [admis_cost_csc_hv_1] + [bed_cost_csc_hv_1] + [drugs_cost_csc_hv_1] + [consult_cost_csc_hv_1] + [invest_cost_csc_hv_1] + [food_cost_csc_hv_1] + [transport_cost_csc_hv_1] + [wages_lost_csc_hv_1] + [additonal_cost_csc_hv_1] + [other_cost_csc_hv_1]
	386	[hospitalization_complete]	Section Header: *Form Status*<br>Complete?	dropdown<br>0 Incomplete<br>1 Unverified<br>2 Complete
