# Supplementary material for: Determinants and Experiences of Care‐Seeking for Childhood Pneumonia in a Rural Indian Setting: A Mixed‐Methods Study
Source: Health Expect. 2025 Apr 16;28(2):e70263. doi: 10.1111/hex.70263 (PMC12002083; doi:10.1111/hex.70263)
Supplement: Supplementary file 3 — Annexure III knowledge. [file HEX-28-e70263-s002.pdf]

Protect Prevent Treatment Questions

Question	Outcomes
Name of respondent	
Respondent relation	1=Father, 2=Mother, 3=Grandmother, 4=Grand father
Name of child	
Name of father	
Knowledge assessment for under-five pneumonia	
Do you know about under-five pneumonia?	1=Yes, 0=No
If Yes, specify the symptoms of under-five pneumonia	
1. Cough (Khasi)	1=Yes, 0=No
2. Fast Breathing	1=Yes, 0=No
3. Chest Indrawing (or pasliyon ka chalna/Chaati me ghadde padna)	1=Yes, 0=No
4. Stridor (khar khar or khad khad ki Awaaz)	1=Yes, 0=No
5. Difficulty in Breathing	1=Yes, 0=No
Do you know danger sign of pneumonia?	1=Yes, 0=No
Tell us the danger signs	
Not able to drink or breastfeed	1=Yes, 0=No
Vomits everything	1=Yes, 0=No
Convulsions	1=Yes, 0=No
Lethargic/Unconsciousness	1=Yes, 0=No
Do you know where to take the child in case you identify and danger sign/symptom of pneumonia?	1=Yes, 0=No
Health and wellness centers(HWCs) /Sub-Centers/Govt. Dispensary	1=Yes, 0=No

Primary Health Centers (PHC)	1=Yes, 0=No
Community Health Centers (CHC)	1=Yes, 0=No
District Hospital (DH)	1=Yes, 0=No
Chemist	1=Yes, 0=No
Local Private Doctor	1=Yes, 0=No
Registered Medical Practitioner	1=Yes, 0=No
Quack	1=Yes, 0=No
Other	
If any other facility, please specify	
Did/Are you exclusively breastfeeding your child for first 6 months?	1=Yes, 0=No
If No, please specify, what did you give	
Janam Ghutti (birth-tonic)	1=Yes, 0=No
Formula Milk	1=Yes, 0=No
Water	1=Yes, 0=No
Cow/Buffalo Milk	1=Yes, 0=No
Did your child get Vitamin A supplementation?	1=Yes, 0=No
Does the family have immunization card available at the time of visit?	1=Yes, 0=No
Pertussis	1=Yes, 2=No, 3=Don't Know
Measles	1=Yes, 2=No, 3=Don't Know
HIB	1=Yes, 2=No, 3=Don't Know
Pneumococcal conjugated vaccine	1=Yes, 2=No, 3=Don't Know
Rotavirus	1=Yes, 2=No, 3=Don't Know

Do you wash your and your child's hand daily before or after meal or after coming from outside?	1=Yes, 0=No
Wash hands without soap	1=Yes, 0=No
Do you have any exposer to the pneumonia awareness activity before ongoing Social behavioral change communication meeting?	1=Yes, 0=No
Exposer through	1=Community, 2=Media, 3=HWC,<br>4=Health Care Centre, 5=Other
Community	1=Accredited Social Health Activist
Media	1=Radio, 2=Pamphlet, 3=Film, 4=Information education communication (IEC) material, 5=TV Advertisement's, 6=Camps, 7=Internet
HWC	1=Community Health officer, 2=Auxiliary Nurse midwives
Health care centers	1=GH, 2=CHC, 3=PHC, 4=Private facility
Any other source of exposer, specify	
