# Supplementary material for: Determinants and Experiences of Care‐Seeking for Childhood Pneumonia in a Rural Indian Setting: A Mixed‐Methods Study
Source: Health Expect. 2025 Apr 16;28(2):e70263. doi: 10.1111/hex.70263 (PMC12002083; doi:10.1111/hex.70263)
Supplement: Supplementary file 4 — Annexure IV translated quantative. [file HEX-28-e70263-s004.pdf]

IR Pneumonia: Enrollment Form

[illegible]	[illegible]
**Instrument: First Day (first_day) साधन: पहला दिन (first_day)**	
[illegible]	[illegible]
[illegible]:	[illegible] (datetime_dmy)
[illegible]	[illegible]<br>1) 201 - [illegible]<br>2) 202 - [illegible]<br>3) 401 - [illegible]<br>4) 203 - [illegible]<br>5) 402 - [illegible]<br>6) 403 - [illegible]<br>7) 204 - [illegible]<br>8) 205 - [illegible]<br>9) 206 – [illegible]<br>10) 207 - [illegible]<br>11) 301 - [illegible]<br>12) 302 - [illegible]<br>13) 303 - [illegible]
[illegible]	[illegible]
[illegible]	[illegible]
[illegible]?	[illegible], [illegible]<br>1 [illegible]<br>0 [illegible]
[illegible], [illegible]	[illegible], [illegible]<br>1 [illegible]<br>2 [illegible]<br>3 [illegible]<br>4 [illegible]
[illegible]?	[illegible]
[illegible]?	[illegible]
[illegible]	[illegible] (date_dmy)
[illegible]	[illegible]
[illegible]	[illegible]
[illegible]	[illegible], [illegible]
[illegible]:	[illegible]<br>1 [illegible]<br>2 [illegible] ([illegible])<br>3 [illegible] ([illegible])
[illegible]:	[illegible]<br>1 [illegible]<br>2 [illegible]<br>3 [illegible]

	4 [illegible]
[illegible]	[illegible]<br>1 [illegible]<br>2 [illegible]<br>3 [illegible]<br>4 [illegible]
[illegible]	[illegible]<br>1 [illegible]<br>2 [illegible]<br>3 [illegible]<br>4 [illegible]<br>5 [illegible]<br>6 [illegible]
[illegible]	[illegible]<br>1 [illegible]<br>2 [illegible]<br>3 [illegible]<br>4 [illegible]<br>5 [illegible]<br>6 [illegible]<br>7 [illegible]<br>8 [illegible]<br>9 [illegible]<br>10 [illegible]<br>11 [illegible]
[illegible] ([illegible])	[illegible]
[illegible] ([illegible])	[illegible]
[illegible]. [illegible]	[illegible]
[illegible]:	[illegible]<br>1 [illegible]<br>2 [illegible]<br>3 [illegible]
[illegible]:	[illegible]<br>1 [illegible]<br>2 [illegible]<br>3 [illegible]
[illegible]:	[illegible]
[illegible]?	[illegible]<br>11 [illegible]<br>12 [illegible]<br>13 [illegible]<br>14 [illegible]<br>15 [illegible]

[illegible]?	[illegible], [illegible]<br>1 [illegible]<br>2 [illegible]<br>3 [illegible]<br>4 [illegible]<br>5 [illegible]<br>6 [illegible]
[illegible]	[illegible]
[illegible]?<br>[illegible]?	[illegible] ([illegible], [illegible]: 1)
[illegible]?<br>?	[illegible]
5 [illegible]?<br>[illegible]?	[illegible] ([illegible], [illegible]: 1)
[illegible]	[illegible], [illegible]
[illegible] ([illegible])	[illegible], [illegible]
[illegible]?	[illegible], [illegible]<br>1 [illegible] ([illegible], [illegible], [illegible])<br>2 [illegible]<br>3 [illegible]<br>4 [illegible]<br>5 [illegible]<br>6 [illegible]<br>7 [illegible]<br>8 [illegible]<br>9 [illegible]/[illegible]<br>10 [illegible]
[illegible], [illegible]	[illegible], [illegible]
[illegible]	[illegible], [illegible]
[illegible] ([illegible])	[illegible], [illegible]
[illegible]?	[illegible], [illegible]<br>1 [illegible] ([illegible], [illegible], [illegible])<br>2 [illegible]<br>3 [illegible]<br>4 [illegible]<br>5 [illegible]<br>6 [illegible]

	7 [illegible]<br>8 [illegible]<br>9 [illegible]/[illegible]<br>10 [illegible]
[illegible], [illegible]	[illegible], [illegible]
[illegible]?<br>[illegible]?	[illegible]
[illegible]?	[illegible], [illegible]<br>1 [illegible]<br>2 [illegible]<br>3 [illegible]-[illegible]<br>4 [illegible]
[illegible]	[illegible]
[illegible]?<br>[illegible]?	[illegible], [illegible]
[illegible]?<br>[illegible]?	[illegible]
[illegible]?<br>[illegible]?	[illegible], [illegible]<br>1 [illegible]/ [illegible]<br>2 [illegible]<br>3 [illegible]<br>4 [illegible]<br>5 [illegible]<br>6 [illegible]<br>7 [illegible]/ [illegible]/<br>[illegible]<br>8 [illegible]<br>9 [illegible]<br>10 [illegible]<br>11 [illegible]<br>12 [illegible]<br>13 [illegible]
[illegible], [illegible]	[illegible]
[illegible]?	[illegible], [illegible]<br>1 [illegible] ([illegible])<br>2 [illegible]

	3 [illegible]<br>4 [illegible]<br>5 [illegible]<br>6 [illegible]
[illegible], [illegible]?<br>[illegible]?	[illegible]<br>1 [illegible]<br>0 [illegible]
[illegible]?<br>[illegible]?	[illegible], [illegible]<br>1 [illegible]<br>2 [illegible]<br>3 [illegible]<br>4 [illegible]<br>5 [illegible]<br>6 [illegible]<br>7 [illegible]<br>([illegible]/[illegible]/[illegible]/[illegible]/[illegible]/[illegible])<br>8 [illegible]<br>9 [illegible]<br>10 [illegible]<br>11 [illegible]
[illegible], [illegible]	[illegible], [illegible]
[illegible]?<br>[illegible]?	[illegible], [illegible]<br>1 [illegible]<br>2 [illegible]<br>3 [illegible]<br>4 [illegible]<br>5 [illegible] ([illegible]) [illegible]<br>6 [illegible]
[illegible], [illegible]	[illegible], [illegible]
[illegible]?<br>[illegible]?	[illegible], [illegible]<br>1 [illegible]<br>2 [illegible]<br>3 [illegible]/[illegible]<br>4 [illegible]<br>5 [illegible]/[illegible]<br>6 [illegible]<br>7 [illegible]<br>8 [illegible]

	9 [illegible]
[illegible]?	[illegible], [illegible]<br>1 [illegible]<br>2 [illegible]<br>3 [illegible]<br>4 [illegible]/[illegible]<br>5 [illegible]<br>6 [illegible] ([illegible]) [illegible])
[illegible], [illegible]	
[illegible]?<br>[illegible]?	[illegible]<br>1 [illegible]/[illegible]<br>2 [illegible]/[illegible]<br>3 [illegible]/[illegible]<br>4 [illegible]/[illegible], [illegible]<br>5 [illegible]<br>6 [illegible]<br>7 [illegible]/[illegible]<br>8 [illegible]<br>9 [illegible]<br>10 [illegible]/[illegible]<br>11 [illegible]/[illegible]/[illegible]<br>12 [illegible]
[illegible], [illegible]	
[illegible]	[illegible], [illegible]<br>1 [illegible]<br>0 [illegible]
[illegible]	[illegible], [illegible]<br>1 [illegible]<br>0 [illegible]
[illegible]-[illegible]/[illegible]	[illegible]<br>1 [illegible]<br>0 [illegible]
[illegible]	[illegible] ([illegible]), [illegible]

[illegible]?<br>[illegible]?	[illegible], [illegible]<br>1 [illegible]<br>0 [illegible]
[illegible]	[illegible] ([illegible], [illegible]: 1000000000, [illegible]:<br>9999999999)
[illegible]?	[illegible], [illegible]<br>1 [illegible]<br>0 [illegible]
	[illegible]<br>1 [illegible], [illegible]<br>2 [illegible], [illegible]<br>[illegible]
[illegible]	[illegible]<br>1 [illegible]<br>0 [illegible]
[illegible]	[illegible]<br>1 [illegible]<br>0 [illegible]
[illegible]	[illegible]<br>1 [illegible]<br>0 [illegible]
[illegible]	[illegible]<br>1 [illegible]<br>0 [illegible]
[illegible]	[illegible]<br>1 [illegible]<br>0 [illegible]
[illegible]	[illegible]<br>1 [illegible]<br>0 [illegible]
[illegible]	[illegible]<br>1 [illegible]<br>0 [illegible]
[illegible]	[illegible]<br>1 [illegible]<br>0 [illegible]
[illegible]	[illegible]<br>1 [illegible]<br>0 [illegible]
[illegible]	[illegible]<br>1 [illegible]

	0 [illegible]
[illegible]	[illegible]<br>1 [illegible]<br>0 [illegible]
[illegible]?<br>[illegible]?	[illegible]<br>1 [illegible]<br>0 [illegible]
[illegible]?<br>[illegible], [illegible]/[illegible]?	[illegible]<br>1 [illegible]<br>0 [illegible]
[illegible]	[illegible]<br>1 [illegible]<br>0 [illegible]
[illegible]	[illegible]<br>1 [illegible]<br>0 [illegible]
[illegible]	[illegible]<br>1 [illegible]<br>0 [illegible]
[illegible]	[illegible]<br>1 [illegible]<br>0 [illegible]
[illegible]	[illegible]<br>1 [illegible]<br>0 [illegible]
[illegible]	[illegible]<br>1 [illegible]<br>0 [illegible]
[illegible]	[illegible]<br>1 [illegible]<br>0 [illegible]
[illegible]	[illegible]<br>1 [illegible]<br>0 [illegible]
[illegible], [illegible]	
[illegible]?<br>[illegible]?	[illegible]<br>1 [illegible]<br>2 [illegible]<br>3 [illegible]<br>4 [illegible]
[illegible]	
[illegible]	
[illegible]	

[illegible]?<br>[illegible], [illegible]?	[illegible]<br>1 [illegible]<br>2 [illegible]/[illegible]<br>3 [illegible]/[illegible]<br>[illegible]<br>4 [illegible]<br>5 [illegible]<br>6 [illegible]
[illegible]	
[illegible]	[illegible]<br>1 [illegible]___1 1<br>2 [illegible]___2 2<br>3 [illegible]___3 3<br>4 [illegible]___4 4<br>5 [illegible]___5 5<br>6 [illegible]___6 6
[illegible]	[illegible]<br>1 [illegible]___1 1<br>2 [illegible]___2 2<br>3 [illegible]___3 3<br>4 [illegible]___4 4<br>5 [illegible]___5 5<br>6 [illegible]___6 6
[illegible]	[illegible]<br>1 [illegible]___1 1<br>2 [illegible]___2 2<br>3 [illegible]___3 3<br>4 [illegible]___4 4<br>5 [illegible]___5 5<br>6 [illegible]___6 6
[illegible]	[illegible]<br>1 [illegible]_[illegible]___1 1<br>2 [illegible]_[illegible]___2 2<br>3 [illegible]_[illegible]___3 3<br>4 [illegible]_[illegible]___4 4<br>5 [illegible]_[illegible]___5 5<br>6 [illegible]_[illegible]___6 6
[illegible]	[illegible]<br>1<br>[illegible]_[illegible]_[illegible]_[illegible]<br>___1 1

	2<br>□□□□□□□□□□_□□_□□□□□□□_□□□□□□□<br>___2 2<br>3<br>□□□□□□□□□□_□□_□□□□□□□_□□□□□□□<br>___3 3<br>4<br>□□□□□□□□□□_□□_□□□□□□□_□□□□□□□<br>___4 4<br>5<br>□□□□□□□□□□_□□_□□□□□□□_□□□□□□□<br>___5 5<br>6<br>□□□□□□□□□□_□□_□□□□□□□_□□□□□□□<br>___6 6
□□□□□□□□□ □□□□□□□□□□<br>□□□□□□□	□□□ □□□□□□<br>1<br>□□□□□□□□□_□□□□□□□□□□_□□□□□□□□<br>___1 1<br>2<br>□□□□□□□□□_□□□□□□□□□□_□□□□□□□□<br>___2 2<br>3<br>□□□□□□□□□_□□□□□□□□□□_□□□□□□□□<br>___3 3<br>4<br>□□□□□□□□□_□□□□□□□□□□_□□□□□□□□<br>___4 4<br>5<br>□□□□□□□□□_□□□□□□□□□□_□□□□□□□□<br>___5 5<br>6<br>□□□□□□□□□_□□□□□□□□□□_□□□□□□□□<br>___6 6
□□□□□□□□□□ □□□□□□□□□□<br>□□□□□□□	□□□ □□□□□□<br>1<br>□□□□□□□_□□□□□□□□□□_□□□□□□□□___<br>1 1<br>2<br>□□□□□□□_□□□□□□□□□□_□□□□□□□□___<br>2 2<br>3<br>□□□□□□□_□□□□□□□□□□_□□□□□□□□___<br>3 3

	4<br>[illegible]_[illegible]_[illegible]___<br>4 4<br>5<br>[illegible]_[illegible]_[illegible]___<br>5 5<br>6<br>[illegible]_[illegible]_[illegible]___<br>6 6
[illegible]/[illegible] [illegible]/[illegible] [illegible] [illegible]	[illegible] [illegible]<br>1 [illegible]_[illegible]_[illegible]___1 1<br>2 [illegible]_[illegible]_[illegible]___2 2<br>3 [illegible]_[illegible]_[illegible]___3 3<br>4 [illegible]_[illegible]_[illegible]___4 4<br>5 [illegible]_[illegible]_[illegible]___5 5<br>6 [illegible]_[illegible]_[illegible]___6 6
[illegible] [illegible] [illegible] [illegible]	[illegible] [illegible]<br>1 [illegible]_[illegible]___1 1<br>2 [illegible]_[illegible]___2 2<br>3 [illegible]_[illegible] ___3 3<br>4 [illegible]_[illegible] ___4 4<br>5 [illegible]_[illegible] ___5 5<br>6 [illegible]_[illegible]___6 6
[illegible] [illegible] ([illegible] + [illegible])	[illegible] [illegible]<br>[illegible] ___1 1<br>[illegible] ___2 2<br>[illegible] ___3 3<br>[illegible] ___4 4<br>[illegible] ___5 5<br>[illegible] ___6 6
[illegible] [illegible] ([illegible] [illegible], [illegible])	[illegible] [illegible]<br>1 [illegible]_[illegible]_[illegible]___1<br>1<br>2 [illegible]_[illegible]_[illegible]___2<br>2<br>3 [illegible]_[illegible]_[illegible]___3<br>3<br>4 [illegible]_[illegible]_[illegible]___4<br>4<br>5 4 [illegible]_[illegible]_[illegible]<br>___5 5<br>6 [illegible]_[illegible]_[illegible]___6<br>6
[illegible] [illegible] ([illegible], [illegible])	[illegible] [illegible]

	1 [illegible]_[illegible]_[illegible]___1 1<br>2 [illegible]_[illegible]_[illegible]___2 2<br>3 [illegible]_[illegible]_[illegible]___3 3<br>4 [illegible]_[illegible]_[illegible]___4 4<br>5 [illegible]_[illegible]_[illegible]___5 5<br>6 [illegible]_[illegible]_[illegible]___6 6
[illegible] [illegible] ([illegible], [illegible].[illegible].[illegible].[illegible].)	
[illegible] [illegible] ([illegible])	
[illegible] [illegible] ([illegible] [illegible] [illegible]/[illegible] [illegible]) [illegible])	[illegible] [illegible]<br>1 [illegible]_[illegible]_[illegible]_[illegible]___1 1<br>2 [illegible]_[illegible]_[illegible]_[illegible]___2 2<br>3 [illegible]_[illegible]_[illegible]_[illegible]___3 3<br>4 [illegible]_[illegible]_[illegible]_[illegible]___4 4<br>5 [illegible]_[illegible]_[illegible]_[illegible]___5 5<br>6 [illegible]_[illegible]_[illegible]_[illegible]___6 6
[illegible] [illegible] [illegible]/[illegible]	[illegible] [illegible]<br>1 [illegible] [illegible] [illegible] [illegible]___1 1<br>2 [illegible] [illegible] [illegible] [illegible]___2 2<br>3 [illegible] [illegible] [illegible] [illegible]___3 3<br>4 [illegible] [illegible] [illegible] [illegible]___4 4<br>5 private_nursing_home_hospi___5 5

	6 [illegible]<br>___6 6
[illegible]	[illegible]<br>1 [illegible]_[illegible]___1 1<br>2 [illegible]_[illegible]___2 2<br>3 [illegible]_[illegible]___3 3<br>4 [illegible]_[illegible]___4 4<br>5 [illegible]_[illegible]___5 5<br>6 [illegible]_[illegible]___6 6
[illegible], [illegible]	
[illegible]?<br>[illegible]	[illegible], [illegible]<br>1 [illegible]<br>2 [illegible]<br>3 [illegible]<br>4 [illegible]<br>5 [illegible]<br>6 [illegible]<br>7 [illegible]<br>8 [illegible]<br>9 [illegible]
[illegible]?<br>[illegible]?	[illegible]<br>1 [illegible]<br>0 [illegible]
[illegible]?<br>[illegible]?	[illegible]<br>1 [illegible]<br>0 [illegible]
[illegible]?<br>[illegible]?	[illegible]<br>1 [illegible]<br>0 [illegible]
[illegible]: -	[illegible]<br>1 [illegible]<br>2 [illegible]<br>3 [illegible]
[illegible]?	[illegible]<br>1 [illegible]<br>2 [illegible]([illegible]) [illegible]).<br>3 [illegible]
[illegible]?	[illegible]<br>1 [illegible]<br>0 [illegible]

[illegible]?	[illegible]<br>1 [illegible]<br>0 [illegible]
[illegible]?	[illegible]<br>1 [illegible]<br>0 [illegible]
[illegible]<br>जेंटामाइसिन	[illegible]
[illegible]	[illegible] ([illegible])
[illegible], [illegible]	[illegible] ([illegible], [illegible]: 1, [illegible]: 12)
[illegible] ([illegible] 0 [illegible])<br>[illegible])[illegible]	[illegible] ([illegible], [illegible]: 0, [illegible]: 12)
[illegible]	[illegible] ([illegible], [illegible]: 1)
[illegible]?<br>[illegible]?	
[illegible], [illegible]	[illegible] ([illegible], [illegible]: 1), [illegible]
[illegible] (0 [illegible])<br>[illegible])	
[illegible]<br>Gentamycin [illegible]<br>[illegible] ?	

[illegible]?<br>[illegible]/[illegible]	[illegible]<br>1 [illegible]<br>0 [illegible]
[illegible], [illegible]?	
[illegible]?<br>([illegible]/[illegible]) [illegible]	[illegible], [illegible]<br>1 [illegible]<br>0 [illegible]
[illegible]?	[illegible], [illegible]<br>1 [illegible]<br>0 [illegible]
[illegible]	[illegible], [illegible]<br>1 [illegible], [illegible]<br>[illegible]<br>2 [illegible], [illegible]<br>[illegible]<br>3 [illegible]<br>[illegible]<br>4 [illegible]
[illegible]	
[illegible]?	[illegible]<br>0 [illegible]<br>1 [illegible]<br>2 [illegible]
**Instrument: Compliance Eighth Day (compliance_eighth_day) साधन: अनुपालन आठवां दिन (अनुपालन_आठवां_दिन)**	
[illegible]:	
[illegible]	[illegible]<br>1 201 - [illegible]<br>2 202 - [illegible]<br>3 401 - [illegible]<br>4 203 - [illegible]<br>5 402 - [illegible]<br>6 403 - [illegible]<br>7 204 - [illegible]<br>8 205 - [illegible]<br>9 206 - [illegible]

	10 207 - [illegible]<br>11 301 - [illegible]<br>12 302 - [illegible]<br>13 303 - [illegible]
[illegible]?	[illegible], [illegible]<br>1 [illegible]<br>0 [illegible]
[illegible], [illegible]	[illegible], [illegible]<br>1 [illegible]<br>2 [illegible]<br>3 [illegible]<br>4 [illegible]
[illegible]?	
[illegible]?	
[illegible]	([illegible]_[illegible])
[illegible]	[illegible]
[illegible]	
[illegible]?	[illegible]<br>1 [illegible]<br>0 [illegible]
[illegible]?	[illegible]<br>1 [illegible]<br>0 [illegible]
[illegible]	[illegible]
[illegible]	
[illegible], [illegible]	[illegible] ([illegible], [illegible]: 1,<br>[illegible]: 12)
[illegible] ([illegible] 0<br>[illegible])<br>[illegible])	[illegible] ([illegible], [illegible]: 0,<br>[illegible]: 12)

[illegible]	[illegible] ([illegible], [illegible]: 1)
[illegible]?<br>[illegible]?	
[illegible], [illegible]	[illegible] ([illegible], [illegible]: 1),<br>[illegible]
[illegible] (0 [illegible])<br>[illegible])	[illegible])<br>[illegible] ([illegible]), [illegible]
[illegible] ?	
[illegible]?<br>[illegible]/[illegible]	[illegible]<br>1 [illegible]<br>0 [illegible]
[illegible], [illegible]?	
[illegible]?<br>([illegible]/[illegible]) [illegible]	[illegible], [illegible]<br>1 [illegible]<br>0 [illegible]
[illegible]?	[illegible], [illegible]<br>1 [illegible]<br>0 [illegible]
[illegible]	[illegible], [illegible]<br>1 [illegible], [illegible]<br>2 [illegible], [illegible]<br>3 [illegible]

	4 □□□□
□□□□ □□□□□□□□□□ □□□□	
□□□□□ □□ □□□□□□□ □□□□□□□ □□□□ □□?	□□□□□□□□□, □□□□□□<br>1 □□□□□<br>2 □□□ □□□□ □□□/□□□ □□ □□□□□ □□□
□□□□ □□□□□ □□□ □□□□□ □□□ □□ □□ □□ □□□□ □□□□□ □□□? □□□□□□□□□□ □□□□□:	□□□□□ □□□□<br>1 □□□□□□□ □□□□□ □□ □□□□ □□□□ □□□ □□□□□□<br>2 □□□□□<br>3 □□□□□□□/□□□□□□□<br>4 □□□□□<br>5 □□□□ □□□□ □□□□□□□<br>6 □□□□ □□□□ □□□□<br>7 □□□□□<br>8 □□□□<br>9 □□□□□
□□□□?	□□□□□ □□□□<br>0 □□□□□□<br>1 □□□□□□□□□□<br>2 □□□□□
**Instrument: Sixteenth Day (sixteenth_day) साधन: सोलहवाँ दिन (सोलहवाँ दिन)**	
□□□□□ □□□□ □□ □□□□:	(□□□□□□_□□□_dmy)
□□□□□□□□□ □□□ □□□□□ □□□	□□□□□ □□□□<br>1 201 - □□□□<br>2 202 - □□□□□ □□□□<br>3 401 - □□□□□ □□□<br>4 203 - □□□□□<br>5 402 - □□□□□ □□□□□<br>6 403 - □□□□□ □□□□<br>7 204 - □□□□□□□□ □□□□<br>8 205 - □□□□□<br>9 206 - □□□□□<br>10 207 - □□□□<br>11 301 - □□□□□<br>12 302 - □□□□□□<br>13 303 - □□□□
□□□□ □□□□□ □□□□□□ □□?	□□□ □□□□<br>1 □□□<br>0 □□□□
□□□ □□□□□ □□□□□□ □□□□ □□, □□ □□□□□ □□□□ □□□□□	□□□□□ □□□□<br>1 □□□□□□□□ □□□□<br>2 □□□□□□□ □□□□□□□□ □□ □□□□ □□□□□□□□□□□□<br>3 □□□□□□<br>4 □□□□

[illegible]?	
[illegible]?	
[illegible]	
[illegible]?	[illegible]<br>1 [illegible]<br>2 [illegible]<br>3 [illegible]
[illegible]	([illegible]_[illegible])
3[illegible]?<br>[illegible]:	[illegible]<br>1 [illegible]<br>2 [illegible]<br>3 [illegible]/[illegible]<br>[illegible]<br>5 [illegible]<br>6 [illegible]<br>7 [illegible]<br>8 [illegible]<br>9 [illegible]
[illegible]?<br>[illegible]?	[illegible], [illegible]<br>1 [illegible]<br>0 [illegible]
[illegible] ?	[illegible] ([illegible], [illegible]: 1),<br>[illegible]
[illegible]?	
[illegible]?	[illegible]<br>0 [illegible]<br>1 [illegible]<br>2 [illegible]

Baseline Form: IR Pneumonia

**सवाल**	**विकल्प**
**Instrument: Base (base) साधन: आधार (बेस)**	
रिकॉर्ड आईडी	
भरने की तिथि	
सी.एच.सी	• सी एच पलवल<br>• दुधोला<br>• अलावलपुर<br>• हाथिन<br>• औरंगाबाद<br>• एसडीएच होडल<br>• सोंदहद<br>• जिले से बाहर
पी.एच.सी	• सीएच पलवल<br>• पलवल शहर<br>• श्याम नगर<br>• कृष्ण कॉलोनी<br>• दूधोला<br>• अल्लीका<br>• रसूलपुर<br>• अलावलपुर<br>• अमरपुर<br>• सिहोल<br>• सोलरा<br>• हथिन<br>• कलसड़ा<br>• मंडकोला<br>• छैंसा<br>• उत्तरावर<br>• नागल जाट<br>• कोट<br>• औरंगाबाद<br>• दीघोट<br>• होडल<br>• सोंधद<br>• भूलवाना<br>• हसनपुर<br>• टप्पा<br>• जिले से बाहर

गांव का नाम	
कर्मचारी कोड	• 201 पंचम 00001-00400<br>• 202 कृष्ण डागर 00401-00800<br>• 401 दुर्ग पाल 00801-01200<br>• 402 भूपेश 01201-01600<br>• 403 प्रेम किशोर 01601-02000<br>• 404 हरफूल 02001-02400<br>• 405 रामेश्वर साहू 02401-02800<br>• 406 यशपाल 02801-03200<br>• 407 सुनील 03201-03600
पहचान	
घरेलू पहचान	
गली संख्या	
मकान नंबर	
वार्ड नंबर	
पता + लैंडमार्क	
क्या घर पर कोई मुखबिर उपलब्ध है?	• नहीं<br>• हां
यदि नहीं तो कृपया कारण चुनें	• घर बंद<br>• किसी ने जवाब नहीं दिया<br>• इनकार<br>• अन्य
यदि अन्य, तो निर्दिष्ट करें	
प्राथमिक संपर्क नंबर	
परिवार के मुखिया का नाम	
परिवार का प्रकार	• संयुक्त<br>• तीन पीढ़ी<br>• नाभिकीय / परमाणु
घर में परिवार के सदस्यों की संख्या	
कल रात घर में रहने वाले व्यक्तियों की संख्या	
क्या घर में कल रात रुके परिवार के सदस्यों से अधिक व्यक्ति मौजूद हैं?	
कितने दिनों से घर में अतिरिक्त व्यक्ति रह रहे हैं?	
घर में संपत्ति	• बिजली<br>• गद्दा<br>• प्रेशर कुकर<br>• कुर्सी<br>• खाट या बिस्तर

	• मेज<br>• इलेक्ट्रिक पंखा भले ही खराब हो<br>• रेडियो या ट्रांजिस्टर भले ही खराब हो<br>• ब्लैक एंड व्हाइट टेलीविजन भले ही खराब हो<br>• रंगीन टेलीविजन भले ही खराब हो<br>• सिलाई मशीन भले ही खराब हो<br>• मोबाइल फोन भले ही खराब हो<br>• लैंडलाइन फोन भले ही खराब हो<br>• इंटरनेट<br>• कंप्यूटर भले ही खराब हो<br>• फ्रिज भले ही खराब हो<br>• ए.सी. / कूलर<br>• वॉशिंग मशीन<br>• घड़ी भले ही खराब हो<br>• साइकिल भले ही खराब हो<br>• मोटरसाइकिल या स्कूटर भले ही खराब हो<br>• पशु-चालित गाड़ी भले ही खराब हो<br>• कार भले ही खराब हो<br>• पानी का पंप भले ही खराब हो<br>• थ्रेशर भले ही खराब हो<br>• ट्रैक्टर भले ही खराब हो
खाना पकाने के लिए घर में मुख्यतः किस प्रकार के ईंधन का उपयोग किया जाता है?	• बिजली<br>• तरलीकृत पेट्रोलियम गैस / प्राकृतिक गैस<br>• मिट्टी का तेल<br>• कोयला / लिग्नाइट<br>• लकड़ी का कोयला<br>• लकड़ी<br>• पुआल / झाड़ी / घास<br>• कृषि फसल अपशिष्ट<br>• गोबर के उपले<br>• बायोगैस<br>• अन्य
यदि खाना पकाने का ईंधन "अन्य" है, तो स्पष्ट करें	
फर्श की मुख्य सामग्री	• कीचड़ / मिट्टी / धरती<br>• रेत<br>• गोबर<br>• कच्ची लकड़ी की तख्तियां<br>• ताड़ / बांस

	• ईंट<br>• पत्थर<br>• पार्केट या पॉलिश की हुई लकड़ी<br>• विनाइल या डामर<br>• सिरेमिक टाइल्स<br>• सीमेंट<br>• पॉलिश किया हुआ पत्थर / संगमरमर / ग्रेनाइट<br>• अन्य
यदि फर्श की मुख्य सामग्री "अन्य" है, तो निर्दिष्ट करें	
छत की मुख्य सामग्री	• कोई छत नहीं<br>• खप्पर / ताड़ पत्ता / सरकंडा / घास<br>• मिट्टी<br>• घास-मिट्टी का मिश्रण<br>• प्लास्टिक / पॉलीथिन शीटिंग<br>• ग्रामीण चटाई<br>• ताड़ / बांस<br>• कच्ची लकड़ी की तख्तियां / लट्ठे<br>• अधपकी ईंट<br>• ढीले पैक किए हुए पत्थर<br>• धातु<br>• लकड़ी<br>• कैलामाइन / सीमेंट फाइबर<br>• एस्बेस्टस शीट<br>• आर.सी.सी. / प्रबलित ईंट कंक्रीट / सीमेंट / कंक्रीट<br>• छत की पतली टाइलें<br>• टाइल्स<br>• स्लेट<br>• पकी हुई ईंट<br>• गट्टर पत्थर<br>• अन्य
यदि छत की मुख्य सामग्री "अन्य" है, तो निर्दिष्ट करें	
बाहरी दीवारों की मुख्य सामग्री	• कोई दीवार नहीं<br>• बेंत / ताड़ / तना / बांस<br>• मिट्टी<br>• घास / सरकंडा / खप्पर

	• बांस और मिट्टी<br>• मिट्टी के साथ पत्थर<br>• प्लाईवुड<br>• गत्ता<br>• अधपकी ईंट<br>• कच्ची लकड़ी / पुनः प्रयुक्त लकड़ी<br>• सीमेंट / कंक्रीट<br>• चूना / सीमेंट के साथ पत्थर<br>• पकी हुई ईंटें<br>• सीमेंट ब्लॉक<br><br>• लकड़ी की तख्तियां / छत की लकड़ी की पट्टियां<br>• गैल्वेनाइज्ड आयरन / धातु / एस्बेस्टस शीट<br><br>• अन्य
यदि बाहरी दीवारों की मुख्य सामग्री "अन्य" है, तो निर्दिष्ट करें	
क्या यह घर आपका है?	• 0. नहीं<br>• 1. हां
परिवार इकाइयों की संख्या	
इकाई प्रमुख का नाम	
बच्चों की संख्या, आयु 0-6 दिन	
7-59 दिन की आयु वाले शिशुओं की संख्या	
2 से 59 महीने की आयु के बच्चों की संख्या	
इस परिवार इकाई में बच्चों की संख्या, आयु < 59 महीने	
पात्र इकाइयों की संख्या	
पूरा?	• अधूरा<br>• असत्यापित<br>• पूर्ण
**Instrument: Unit (unit) उपकरण: इकाई (यूनिट)**	
सूचना देनेवाला	• माता<br>• पिता<br>• अन्य परिवार के सदस्य<br>• अन्य अभिभावक/संरक्षक<br>• माता और पिता दोनों
यदि मुखबिर कोई अन्य हो तो कृपया बताएं	
कृपया कारण बताएं कि माँ घर पर क्यों नहीं हैं?	
पिता का नाम	

पिता की आयु	
पिता की स्कूली शिक्षा का वर्ष	• प्राथमिक (5वीं) से कम / निरक्षर<br>• प्राथमिक (5वीं)<br>• प्राथमिक (5वीं) से अधिक लेकिन माध्यमिक (10वीं) से कम<br>• माध्यमिक (10वीं)<br>• उच्च माध्यमिक (12वीं) / डिप्लोमा<br>• स्नातक<br>• स्नातकोत्तर<br>• स्नातकोत्तर से अधिक
पिता का वर्तमान व्यवसाय	• सरकारी सेवा<br>• निजी सेवा<br>• स्व-नियोजित<br>• केवल कृषि<br>• दैनिक मजदूरी<br>• कार्य नहीं करता/करती<br>• लागू नहीं
पिता का धर्म	• ईसाई<br>• मुस्लिम<br>• हिंदू<br>• सिख<br>• बौद्ध/नव-बौद्ध<br>• जैन<br>• कोई नहीं<br>• अन्य
यदि अन्य हो तो निर्दिष्ट करें	
पिता का जातीय समूह जाति/जनजाति	• अनुसूचित जाति<br>• अनुसूचित जनजाति<br>• अन्य पिछड़ा वर्ग<br>• सामान्य वर्ग<br>• इनमें से कोई नहीं
द्वितीयक संपर्क नंबर	
द्वितीयक संपर्क का संबंध	• चाचा / मामा / ताऊ / फूफा<br>• चाची / मामी / ताई / बुआ<br>• दादा / नाना<br>• दादी / नानी<br>• पिता<br>• माता<br>• अन्य
अन्य हैं, तो कृपया स्पष्ट करें	

माता/प्राथमिक देखभालकर्ता की आयु वर्ष	
माँ या प्राथमिक देखभालकर्ता के स्कूली शिक्षा के वर्ष	• प्राइमरी (5वीं) से कम / निरक्षर<br>• प्राइमरी (5वीं)<br>• प्राइमरी (5वीं) से ऊपर लेकिन सेकेंडरी (10वीं) से कम<br>• सेकेंडरी (10वीं)<br>• उच्च माध्यमिक (12वीं) / डिप्लोमा<br>• स्नातक<br>• स्नातकोत्तर<br>• स्नातकोत्तर से ऊपर
माँ या प्राथमिक देखभालकर्ता का वर्तमान व्यवसाय	• सरकारी सेवा<br>• निजी सेवा<br>• स्वरोजगार<br>• केवल खेती<br>• दैनिक मजदूर<br>• कार्य नहीं करता/करती<br>• गृहिणी
क्या बच्चा निम्नलिखित भोजन का सेवन करता है	• अंडे<br>• दूध<br>• मटन<br>• मछली<br>• मुर्गा<br>• अन्य पशु मांस<br>• फल<br>• सब्ज़ियाँ
आपके घर के सदस्यों के लिए पीने के पानी का मुख्य स्रोत क्या है?	• पानी पहुंचाया<br>• सार्वजनिक नल<br>• ट्यूबवेल या बोरहोल या हैंडपंप<br>• खुला कुआं<br>• बंद कुआं<br>• झरने से पानी<br>• वर्षा का पानी<br>• टैंकर ट्रक<br>• टैंक के साथ छोटी गाड़ी<br>• सतही जल [नदी/बांध/झील/तालाब/धारा/नहर]<br>• बोतलबंद जल<br>• अन्य
यदि जल का स्रोत "अन्य" है, तो निर्दिष्ट करें	

क्या परिवार आरओ फ़िल्टरेशन का उपयोग करता है?	• 1 हाँ<br>• 0 नहीं
यदि हां, तो कब से	• एक वर्ष से कम<br>• 1 - 2 वर्ष<br>• 2 - 3 वर्ष<br>• 3 - 4 वर्ष<br>• 4 - 5 वर्ष<br>• 5 वर्ष से अधिक
शौचालय का प्रकार	• स्वतंत्र<br>• अन्य घरों के साथ साझा किया गया<br>• खुला/बिना शौचालय
घर में किस प्रकार की शौचालय सुविधा उपलब्ध है?	• फ्लश या उश शौचालय डालना<br>• गड्ढा शौचालय<br>• शुष्क शौचालय<br>• ट्विन पिट/कम्पोस्टिंग शौचालय<br>• शौचालय की सुविधा/खुली जगह नहीं<br>• अन्य
यदि शौचालय सुविधा "अन्य" है, तो निर्दिष्ट करें	
क्या इस घर के किसी सामान्य सदस्य के पास बैंक खाता है?	• 1 हाँ<br>• 0 नहीं
क्या इस घर के किसी सामान्य सदस्य का डाक घर खाता है?	• 1 हाँ<br>• 0 नहीं
गरीबी रेखा से नीचे का कार्ड (बीपीएल कार्ड) रखें	• 1 हाँ<br>• 0 नहीं
क्या इस परिवार का कोई सामान्य सदस्य किसी स्वास्थ्य योजना या स्वास्थ्य बीमा के अंतर्गत आता है?	• 1 हाँ<br>• 0 नहीं
कृपया योजना का चयन करें: -	• आयुष्मान भारत योजना<br>• सीजीएचएस<br>• ईसीएचएस<br>• ईएसआई<br>• निजी
यदि बीमा निजी कंपनी द्वारा कवर किया गया है तो कृपया (कंपनी का नाम) बताएं	
बीमा योजना के अंतर्गत बीमित व्यक्ति का नाम?	
बीमा योजना के अंतर्गत कवर सदस्यों की संख्या	
क्या आपने आयुष्मान भारत योजना के तहत कोई लाभ लिया?	• 1 हाँ<br>• 0 नहीं
आपने आयुष्मान भारत योजना का लाभ कब लिया?	

और किस बीमारी के लिए?	
वार्षिक आय	
पूरा?	• अपूर्ण<br>• असत्यापित<br>• पूर्ण
**Instrument: Child (child) साधन: बच्चा (बच्चा)**	
क्या पिछले महीने बच्चों को कभी कोई बीमारी हुई थी?	• हाँ<br>• नहीं
बीमार बच्चे का नाम	
पिता का नाम	
लिंग	• पुरुष<br>• महिला
जन्म तिथि	
बच्चे की आयु (दिनों में)	
क्या बच्चा कम वजन का पैदा हुआ था?	• 1 हाँ<br>• 0 नहीं
बच्चे का वजन ग्राम में	
क्या बच्चा समय से पहले पैदा हुआ था?	• 1 हाँ<br>• 0 नहीं
जन्म के समय गर्भावधि आयु (सप्ताह में)	
लक्षण कब पहचाने गए?	
लक्षण पहचाने जाने पर बच्चे की आयु क्या थी?	
लक्षण पहचाने जाने से पहले कितने दिन लगे?	
खाँसी	• 1 हाँ<br>• 0 नहीं
बीमारी की सूचना दी गई	• सहज<br>• संकेत दिया गया
कितने दिन से?	
लक्षण कितने समय तक बना रहता है?	
सर्दी/नाक बहना	• 1 हाँ<br>• 0 नहीं
बीमारी की सूचना दी गई	• अविरल<br>• के लिए प्रेरित किया
कितने दिन से	•
साँस लेने में कठिनाई	• हाँ<br>• नहीं
बीमारी की सूचना दी गई	• अविरल<br>• के लिए प्रेरित किया
कितने दिन से?	

लक्षण कितने समय तक बना रहता है?	
चेस्ट इनड्राइंग / पसली चालना	• हाँ<br>• नहीं
बीमारी की सूचना दी गई	• अविरल<br>• के लिए प्रेरित किया
कितने दिन से?	•
लक्षण कितने समय तक बना रहता है?	•
तेज़ साँस लेना	• हाँ<br>• नहीं
बीमारी की सूचना दी गई	• अविरल<br>• के लिए प्रेरित किया
कितने दिन से	
लक्षण कितने समय तक बना रहता है?	
घरघराहट	• हाँ<br>• नहीं
बीमारी की सूचना दी गई	• अविरल<br>• के लिए प्रेरित किया
कितने दिन से	
स्तनपान कराने/पानी पीने/खाना बंद करने में असमर्थ होना	• हाँ<br>• नहीं
बीमारी की सूचना दी गई	• अविरल<br>• के लिए प्रेरित किया
कितने दिन से	
उल्टी करना	• हाँ<br>• नहीं
बीमारी की सूचना दी गई	• अविरल<br>• के लिए प्रेरित किया
कितने दिन से	
आक्षेप	• हाँ<br>• नहीं
बीमारी की सूचना दी गई	• अविरल<br>• के लिए प्रेरित किया
कितने दिन से	
सुस्त	• हाँ<br>• नहीं
बीमारी की सूचना दी गई	• अविरल<br>• के लिए प्रेरित किया
कितने दिन से	
अचेत	• हाँ

	• नहीं
बीमारी की सूचना दी गई	• अविरल<br>• के लिए प्रेरित किया
कितने दिन से	
स्टिरडोर (सांस लेने में ध्वनि)	• हाँ<br>• नहीं
बीमारी की सूचना दी गई	• अविरल<br>• के लिए प्रेरित किया
कितने दिन से	
तापमान 37.5°C से अधिक	• हाँ<br>• नहीं
बीमारी की सूचना दी गई	• अविरल<br>• के लिए प्रेरित किया
कितने दिन से	
शरीर का कम तापमान / हाइपोथर्मिया	• हाँ<br>• नहीं
बीमारी की सूचना दी गई	• अविरल<br>• के लिए प्रेरित किया
कितने दिन से	
spO2 < 90%	• हाँ<br>• नहीं
बीमारी की सूचना दी गई	• अविरल<br>• के लिए प्रेरित किया
कितने दिन से	
दस्त	• हाँ<br>• नहीं
बीमारी की सूचना दी गई	• अविरल<br>• के लिए प्रेरित किया
कितने दिन से	
निर्जलीकरण	• हाँ<br>• नहीं
बीमारी की सूचना दी गई	• अविरल<br>• के लिए प्रेरित किया
कितने दिन से	
बेचैन/चिड़चिड़ा	• हाँ<br>• नहीं
बीमारी की सूचना दी गई	• अविरल<br>• के लिए प्रेरित किया
कितने दिन से	

मल में रक्त	• हाँ<br>• नहीं
बीमारी की सूचना दी गई	• अविरल<br>• के लिए प्रेरित किया
कितने दिन से	
गर्दन में अकड़न	• हाँ<br>• नहीं
बीमारी की सूचना दी गई	• अविरल<br>• के लिए प्रेरित किया
कितने दिन से	
आँख से स्राव	• हाँ<br>• नहीं
बीमारी की सूचना दी गई	• अविरल<br>• के लिए प्रेरित किया
कितने दिन से	
कान से स्राव	• हाँ<br>• नहीं
बीमारी की सूचना दी गई	• अविरल<br>• के लिए प्रेरित किया
कितने दिन से	
त्वचा संक्रमण	• हाँ<br>• नहीं
बीमारी की सूचना दी गई	• अविरल<br>• के लिए प्रेरित किया
कितने दिन से	
उभरा हुआ फॉन्टेनेल	• हाँ<br>• नहीं
बीमारी की सूचना दी गई	• अविरल<br>• के लिए प्रेरित किया
कितने दिन से	
बहुत कमज़ोर / कुपोषण	• हाँ<br>• नहीं
बीमारी की सूचना दी गई	• अविरल<br>• के लिए प्रेरित किया
कितने दिन से	
खसरा	• हाँ<br>• नहीं
बीमारी की सूचना दी गई	• अविरल<br>• के लिए प्रेरित किया

कितने दिन से	
मुंह में अल्सर	• हाँ<br>• नहीं
बीमारी की सूचना दी गई	• अविरल<br>• के लिए प्रेरित किया
कितने दिन से	
नाभि लाल हो जाना या उसमें से मवाद निकलना	• हाँ<br>• नहीं
बीमारी की सूचना दी गई	• अविरल<br>• के लिए प्रेरित किया
कितने दिन से	
त्वचा पर फुंसियां	• हाँ<br>• नहीं
बीमारी की सूचना दी गई	• अविरल<br>• के लिए प्रेरित किया
कितने दिन से	
पीले तलवे	• हाँ<br>• नहीं
बीमारी की सूचना दी गई	• अविरल<br>• के लिए प्रेरित किया
कितने दिन से	
अन्य	• हाँ<br>• नहीं
बीमारी की सूचना दी गई	• अविरल<br>• के लिए प्रेरित किया
कितने दिन से	
अन्य निर्दिष्ट करें	
यदि बच्चे को तेज सांस लेने/सांस लेने में कठिनाई/छाती में खिंचाव/कर्कश ध्वनि/घरघराहट के साथ खांसी हो तो निम्न बातें पूछें:	
क्या बच्चा बीमारी के दौरान किसी भी दिन ठीक से भोजन नहीं कर पा रहा था या उसने ठीक से भोजन करना बंद कर दिया था	• हाँ<br>• नहीं
क्या बीमारी के दौरान बच्चे की नाक फूली हुई थी	• हाँ<br>• नहीं
क्या बीमारी के दौरान बच्चे को केंद्रीय सायनोसिस था	• हाँ<br>• नहीं
क्या बच्चा शांत होने पर भी स्ट्राइडर से पीड़ित था?	• हाँ<br>• नहीं

क्या बीमारी के दौरान किसी दिन बच्चा केवल उत्तेजित होने पर ही हिलता-डुलता था?	• हाँ<br>• नहीं
क्या बच्चा बिल्कुल भी हिल नहीं रहा था	• हाँ<br>• नहीं
क्या बच्चे को ऐंठन या टीएस था	• हाँ<br>• नहीं
क्या बच्चे के शरीर का तापमान कम था	• हाँ<br>• नहीं
अन्य	• हाँ<br>• नहीं
अन्य निर्दिष्ट करें	• हाँ<br>• नहीं
क्या निमोनिया (तेज़ साँस लेना/साँस लेने में कठिनाई/छाती खींचना) के उपचार से बच्चा ठीक हो गया?	• हाँ<br>• नहीं
यदि नहीं तो बच्चा अब कैसा है?	• तेज़ गति से सांस लेना<br>• छाती के अंदरुनी भाग का बना रहना<br>• बच्चे को इलाज के दौरान अस्पताल में भर्ती कराया गया<br>• SAE विकसित होना (एनाफाइलैक्सिस प्रतिक्रिया, गंभीर दस्त, पूरे शरीर पर गंभीर दाने)<br>• बच्चे की मृत्यु हो गई
क्या बच्चा वर्तमान में कोई दवा ले रहा है?	• हाँ<br>• नहीं
मामला - पहचाना गया	
क्या आपने निमोनिया के लक्षण के लिए घर से बाहर देखभाल, सलाह या उपचार लिया?	• हाँ<br>• नहीं
किस बीमारी के लिए :	• खाँसी<br>• साँस लेने में कठिनाई<br>• तेज़ साँस लेना<br>• न्यूमोनिया<br>• गंभीर छाती अंदर की ओर खींचना<br>• स्ट्रिडोर (ध्वनि श्वास)<br>• घरघराहट 13<br>• दस्त/दस्त<br>• निर्जलीकरण/p हम<br>• धंसी हुई आंखें<br>• बेचैनी/चिड़चिड़ापन<br>• मल में रक्त

	• बुखार 100.4° F<br>• गर्दन में अकड़न<br>• उल्टी करना<br>• स्तनपान/डॉक्टर द्वारा दूध पिलाने में असमर्थ होना ऐंठन<br>• सुस्त<br>• अचेत<br>• स्राव होना<br>• कान से स्राव<br>• त्वचा संक्रमण<br>• उभरा हुआ फ़ॉन्ट<br>• ठंडा/चलना<br>• बहुत कमज़ोर/कुपोषित<br>• खसरा<br>• मुंह में अल्सर<br>• नाभि लाल हो गई, मवाद बह रहा है<br>• त्वचा पर फुंसियां<br>• पीले तलवे<br>• कम शारीरिक तापमान/ हाइपोथर्मिया
क्या उपरोक्त के अलावा कोई अन्य बीमारी है?	• हाँ<br>• नहीं
अन्य निर्दिष्ट करें	• आशा___1<br>• आशा___2<br>• आशा___3<br>• आशा___4<br>• आशा___5<br>• आशा___6
एएनएम	• एएनएम___1<br>• एएनएम___2<br>• एएनएम___3<br>• एएनएम___4<br>• एएनएम___5<br>• एएनएम___6
आंगनवाड़ी कार्यकर्ता	• ओह___1<br>• ओह___2<br>• ओह___3<br>• ओह___4<br>• ओह___5<br>• ओह___6
उप केंद्र	• उप_केन्द्र___1

	• उप_केन्द्र___2<br>• उप_केन्द्र___3<br>• उप_केन्द्र___4<br>• उप_केन्द्र___5<br>• उप_केन्द्र___6
स्वास्थ्य एवं कल्याण केंद्र	• स्वास्थ्य_और_कल्याण_केंद्र___1<br>स्वास्थ्य_और_कल्याण_केंद्र___2<br>स्वास्थ्य_और_कल्याण_केंद्र___3<br>• स्वास्थ्य_और_कल्याण_केंद्र___4<br>• स्वास्थ्य_और_कल्याण_केंद्र___5<br>स्वास्थ्य_और_कल्याण_केंद्र___6
प्राथमिक स्वास्थ्य केंद्र	• प्राथमिक_स्वास्थ्य_केन्द्र___1<br>• प्राथमिक_स्वास्थ्य_केन्द्र___2<br>• प्राथमिक_स्वास्थ्य_केन्द्र___3<br>• प्राथमिक_स्वास्थ्य_केन्द्र___4<br>• प्राथमिक_स्वास्थ्य_केन्द्र___5<br>• प्राथमिक_स्वास्थ्य_केन्द्र___6
सामुदायिक स्वास्थ्य केंद्र	• सामुदायिक_स्वास्थ्य_केन्द्र___1<br>• समुदाय_स्वास्थ्य_केन्द्र___2<br>• समुदाय_स्वास्थ्य_केन्द्र___3<br>• सामुदायिक_स्वास्थ्य_केन्द्र___4<br>• समुदाय_स्वास्थ्य_केन्द्र___5<br>• समुदाय_स्वास्थ्य_केन्द्र___6
जिला/सरकारी अस्पताल/उप जिला अस्पताल	• जिला_सरकारी_अस्पताल___1<br>• जिला_सरकारी_अस्पताल___2<br>• जिला_सरकारी_अस्पताल___3<br>• जिला_सरकारी_अस्पताल___4<br>• जिला_सरकारी_अस्पताल___5<br>• जिला_सरकारी_अस्पताल___6
औषधि विक्रेता की दुकान	• केमिस्ट_शॉप___1<br>• केमिस्ट_शॉप___2<br>• केमिस्ट_शॉप___3<br>• केमिस्ट_शॉप___4<br>• केमिस्ट_शॉप___5<br>• केमिस्ट_शॉप___6
निजी चिकित्सक (एमबीबीएस + विशेषज्ञता)	• प्राइवेट_एमबीबीएस_स्पेसिलाइजेशन___1<br>• प्राइवेट_एमबीबीएस_स्पेसिलाइजेशन___2<br>• प्राइवेट_एमबीबीएस_स्पेसिलाइजेशन___3<br>• प्राइवेट_एमबीबीएस_स्पेसिलाइजेशन___4<br>• प्राइवेट_एमबीबीएस_स्पेसिलाइजेशन___5

	• प्राइवेट_एमबीबीएस_स्पेसिलाइजेशन___6
निजी चिकित्सक (मेडिकल डॉक्टर, एमबीबीएस)	• प्राइवेट_प्रैक्टिशनर_एमबीबीएस___1<br>• प्राइवेट_प्रैक्टिशनर_एमबीबीएस___2<br>• प्राइवेट_प्रैक्टिशनर_एमबीबीएस _3<br>• प्राइवेट_प्रैक्टिशनर_एमबीबीएस___4<br>• प्राइवेट_प्रैक्टिशनर_एमबीबीएस _5<br>• • प्राइवेट_प्रैक्टिशनर_एमबीबीएस___6
निजी चिकित्सक (होम्योपैथी, बीएचएमएस)	• होम्योपैथी_बीएचएमएस___1<br>• होम्योपैथी_बीएचएमएस___2<br>• होम्योपैथी_बीएचएमएस___3<br>• होम्योपैथी_बीएचएमएस___4<br>• होम्योपैथी_बीएचएमएस ___5<br>• होम्योपैथी_बीएचएमएस ___6
निजी चिकित्सक (आयुर्वेद, बी.ए.एम.एस.)	• आयुर्वेद_ बीएचएमएस ___1<br>• आयुर्वेद_ बीएचएमएस ___2<br>• आयुर्वेद बीएचएमएस ___3<br>• आयुर्वेद_ बीएचएमएस ___4<br>• आयुर्वेद_ बीएचएमएस ___5<br>• आयुर्वेद_ बीएचएमएस ___6
निजी चिकित्सक (आरएमपी)	• आरएमपी___1<br>• आरएमपी___2<br>• आरएमपी___3<br>• आरएमपी___4<br>• आरएमपी___5<br>• आरएमपी___6
निजी चिकित्सक (कोई डिग्री नहीं/डिग्री ज्ञात नहीं)	• डिग्री_अज्ञात___1<br>• डिग्री_अज्ञात___2<br>• डिग्री_अज्ञात___3<br>• डिग्री_अज्ञात___4<br>• डिग्री_अज्ञात___5<br>• डिग्री_अज्ञात___6
निजी चिकित्सक (अन्य, निर्दिष्ट करें)	• निजी_अन्य___1<br>• निजी_अन्य___2<br>• निजी_अन्य___3<br>• निजी_अन्य___4<br>• निजी_अन्य___5<br>• निजी_अन्य___6
निजी नर्सिंग होम/अस्पताल	• निजी_नर्सिंग_होम_हॉस्पि_1<br>• निजी_नर्सिंग_होम_हॉस्पि_2<br>• निजी_नर्सिंग_होम_हॉस्पि ___3

	• निजी_नर्सिंग_होम_हॉस्पि ___4<br>• निजी_नर्सिंग_होम_हॉस्पि_5<br>• निजी_नर्सिंग_होम_हॉस्पि_6
नहीं मांगा गया	• नहीं_मांगा___1<br>• नहीं_मांगा___2<br>• नहीं_मांगा___3<br>• नहीं_मांगा___4<br>• नहीं_मांगा___5<br>• नहीं_मांगा___6
यदि हाँ, तो बीमारी की पहचान के कितने समय बाद आपने देखभाल की। दिन {identication_day}	
घंटे	
दिन	
खांसी की दवाई	• देखा<br>• रिपोर्ट
जेंटामाइसिन	• देखा<br>• रिपोर्ट
एमोक्सीसाईक्लिन	• देखा<br>• रिपोर्ट
एंटीबायोटिक दवाओं	• देखा<br>• रिपोर्ट
अज्ञात टैबलेट	• देखा<br>• रिपोर्ट
अज्ञात सिरप	• देखा<br>• रिपोर्ट
अज्ञात पाउडर	• देखा<br>• रिपोर्ट
इंजेक्शन	• देखा<br>• रिपोर्ट
इंजेक्शन अज्ञात	• देखा<br>• रिपोर्ट
साँस लेना	• देखा<br>• रिपोर्ट
नेबुलाइजेशन	• देखा<br>• रिपोर्ट
IV द्रव्य	• देखा<br>• रिपोर्ट
खुमारी भगाने	• देखा<br>• रिपोर्ट

ऑक्सीजन थेरेपी	• देखा<br>• रिपोर्ट
ब्रोंकोडाईलेटर्स	• देखा<br>• रिपोर्ट
जस्ता	• देखा<br>• रिपोर्ट
अन्य बनाम	• देखा<br>• रिपोर्ट
अन्य	• देखा<br>• रिपोर्ट
अन्य निर्दिष्ट करें	
एमोक्सिसिलिन की सलाह किसने दी?	• आशा<br>• एएनएम<br>• एडब्ल्यूडब्ल्यू<br>• उप केंद्र<br>• स्वास्थ्य और कल्याण केंद्र<br>• प्राथमिक स्वास्थ्य केंद्र<br>• सामुदायिक स्वास्थ्य केंद्र<br>• जिला/सरकारी अस्पताल/उप जिला अस्पताल<br>• केमिस्ट की दुकान<br>• निजी चिकित्सक (एमबीबीएस + विशेषज्ञता)<br>• निजी चिकित्सक (मेडिकल डॉक्टर, एमबीबीएस)<br>• निजी चिकित्सक (होम्योपैथी, बीएचएमएस)<br>• निजी चिकित्सक (आयुर्वेद, बीएएमएस)<br>• निजी चिकित्सक (आरएमपी)<br>• निजी व्यवसायी (कोई डिग्री नहीं/डिग्री ज्ञात नहीं)<br>• निजी चिकित्सक (अन्य, निर्दिष्ट करें)<br>• निजी नर्सिंग होम/अस्पताल<br>• अन्य
अन्य (निर्दिष्ट करे	
यदि एमोक्सिसिलिन की सलाह दी गई, तो बच्चे को कितने दिनों तक एमोक्सिसिलिन दी गई	
यदि एमोक्सिसिलिन की सलाह दी जाती है, तो दिन में कितनी बार इसकी सलाह दी जाती है	
आपने कितने दिनों तक एमोक्सिसिलिन दी (यदि नहीं दी तो 0 दर्ज करें)।	

आपने अपने शिशु को दिन में कितनी बार एमोक्सिसिलिन दी?	
जेंटामाइसिन की सलाह किसने दी?	• आशा<br>• एएनएम<br>• एडब्ल्यूडब्ल्यू<br>• उप केंद्र<br>• स्वास्थ्य और कल्याण केंद्र<br>• प्राथमिक स्वास्थ्य केंद्र<br>• सामुदायिक स्वास्थ्य केंद्र<br>• जिला/सरकारी अस्पताल/उप जिला अस्पताल<br>• केमिस्ट की दुकान<br>• निजी चिकित्सक (एमबीबीएस+ विशेषज्ञता)<br>• निजी चिकित्सक (मेडिकल डॉक्टर,एमबीबीएस)<br>• निजी चिकित्सक (होम्योपैथी, बीएचएमएस)<br>• निजी चिकित्सक (आयुर्वेद, बीएएमएस)<br>• निजी चिकित्सक (आरएमपी)<br>• निजी व्यवसायी (कोई डिग्री नहीं/डिग्री ज्ञात नहीं)<br>• निजी चिकित्सक (अन्य, निर्दिष्ट करें)<br>• निजी नर्सिंग होम/अस्पताल 99 अन्य
अन्य (निर्दिष्ट करे	
सलाह सलाह दी गई पालन की गई दिनों की संख्या प्रतिदिन खुराक दिनों की संख्या प्रतिदिन खुराक एमोक्सिसिलिन	
यदि जेंटामाइसिन की सलाह दी गई, तो बच्चे को जेंटामाइसिन कितने दिनों तक दिया गया?	
यदि जेंटामाइसिन की सलाह दी गई है, तो दिन में कितनी बार इसकी सलाह दी गई है	
आपने कितने दिनों तक जेंटामाइसिन दिया (यदि नहीं दिया तो 0 दर्ज करें)	
आपने अपने शिशु को दिन में कितनी बार जेंटामाइसिन दिया?	
क्या आपने स्वास्थ्य सेवा प्रदाताओं द्वारा दी गई सिफारिश के अनुसार दवा (एमोक्सिसिलिन/जेंटामाइसिन) लेना जारी रखा?	• हाँ<br>• नहीं

क्या बीमारी के लिए कोई घरेलू उपचार दिया गया था	• हाँ<br>• नहीं
यदि हां तो क्या	• अदरक, नींबू या तुलसी के पत्तों वाला गर्म पेय, चीनी के साथ या बिना चीनी के<br>• अदरक, नींबू या पुदीने वाला गर्म पेय, चीनी के साथ या बिना चीनी के<br>• सौंफ या इलायची या अदरक वाली चाय चीनी के साथ या बिना चीनी के<br>• अन्य
अन्य निर्दिष्ट करें	
अगर खांसी/तेज़ साँस लेने/साँस लेने में कठिनाई/बुखार के लिए कभी इलाज नहीं करवाया गया, तो पूछें। आपने इलाज क्यों नहीं करवाया (स्वतःस्फूर्त)	• देखभाल के लिए कहां जाएं, यह पता नहीं<br>• पता नहीं किससे संपर्क करें<br>• इस बीमारी के इलाज की उपलब्धता के बारे में जानकारी नहीं<br>• इस बीमारी के लिए इलाज की जरूरत थी इसका एहसास नहीं था<br>• यह नहीं पता था कि सरकारी सुविधाओं पर दवाइयां मुफ्त हैं<br>• अन्य
अन्य निर्दिष्ट करें	
क्या बच्चे को पिछले महीने किसी बीमारी के कारण रेफर किया गया था?	• हाँ<br>• नहीं
यदि हां, तो किस बीमारी के लिए	• खांसी<br>• सांस लेने में कठिनाई<br>• तेज़ साँस लेना<br>• तेज़ साँस < 90%<br>• निमोनिया<br>• दस्त<br>• बुखार 100.4<br>• गंभीर छाती इन्ड्राविंग<br>• अच्छी तरह से खिलाने में असमर्थ<br>• शरीर का कम तापमान<br>• नाभि में मवाद<br>• त्वचा फुंसी<br>• अन्य
निर्दिष्ट करें, अन्य	
बच्चे को किसने रेफर किया?	रेडियो, आवश्यक<br>• आशा<br>• एएनएम<br>• एचडब्ल्यूसी

	• पीएचसी<br>• सीएचसी<br>• सरकारी अस्पताल<br>• निजी अस्पताल<br>• निजी क्लिनिक<br>• अन्य
अन्य निर्दिष्ट करें	
बच्चे को अन्य द्वारा कहां रेफर किया गया था?	रेडियो, आवश्यक<br>• एएनएम<br>• एचडब्ल्यूसी<br>• पीएचसी<br>• सीएचसी<br>• सरकारी अस्पताल<br>• निजी अस्पताल<br>• निजी क्लिनिक<br>• अन्य
क्या आपने रेफरल अनुशंसा का अनुपालन किया स्वास्थ्य देखभाल प्रदाताओं द्वारा बनाई गई?	हाँनहीं, आवश्यक<br>• 1 हाँ<br>• 0 नहीं
यदि नहीं, तो कृपया बताएं	
यदि आपके बच्चे को किसी बीमारी के कारण<br>रेफर किया गया हो<br>पिछले 1 महीने और आप उसे ले गए<br>संदर्भित स्रोत, रेफरल पर क्या हुआ<br>सुविधा	रेडियो, आवश्यक<br>• उन्होंने हमारा मनोरंजन नहीं किया<br>• उन्होंने बच्चे की जांच नहीं की<br>• उन्होंने हमें बिना किसी उपचार के वापस भेज दिया<br>• उन्होंने बच्चे की जांच की और कुछ दवाइयाँ लिखीं/वितरित कीं और भेज दीं हमें वापस<br>• उन्होंने बच्चे की जांच नहीं की बल्कि लिखा कुछ दवाइयां बाहर से खरीदनी होंगी<br>• उन्होंने बच्चे को भर्ती कराया<br>• अन्य
अन्य निर्दिष्ट करें	
यदि आपके बच्चे को किसी बीमारी के कारण<br>रेफर किया गया हो<br>पिछले 1 महीने से और आप उसे स्रोत के पास ले गए<br>रेफरल, आपको सलाह दिए जाने के कितने समय बाद, क्या आपने<br>बच्चे को ले जाएं (घंटों में लिखें)।	

यदि आपने बच्चे को रेफरल के लिए नहीं लिया है तो '999 भरें	
यदि स्वास्थ्य एवं परिवार कल्याण केंद्र/पीएचसी/सीएचसी/जीएच/निजी अस्पताल में ले जाया जाए<br>निमोनिया, तेज़ साँस लेने और छाती के लिए अस्पताल<br>क्या बच्चे को कोई दवा दी गई थी?	रेडियो, आवश्यक<br>• जेंटामाइसिन<br>• एमोक्सिसिलिन<br>• कोई दवा नहीं दी गई
यदि हाँ, तो कितने दिनों तक दवा दी गई?	• पाठ (संख्या), आवश्यक
दी गई दवा की संख्या (प्रतिदिन)	
यदि आप बच्चे को रेफरल के लिए नहीं ले गए तो क्या होगा?<br>कारण थे। (सभी लागू होने वाले का चयन करें)	• चेकबॉक्स, आवश्यक<br>• नहीं परिवहन<br>• कोई नहीं साथ<br>• सरकार सुविधाएं बद किया हुआ<br>• दवाइयाँ उपलब्ध नहीं है सरकार पर सुविधाएँ<br>• डॉक्टर हैं उपलब्ध नहीं है सरकार पर सुविधाएँ<br>• सुविधाएं हैं नहीं उपयुक्त सरकारी केंद्र<br>• निजी सुविधाएं हैं बहुत महँगा<br>• महसूस नहीं हुआ बच्चा बीमार था<br>• महसूस नहीं हुआ कि बच्चा काफी बीमार था रेफरल की आवश्यकता<br>• परिवार सदस्य सा वह रेफरल आवश्यक नहीं<br>• पता नहीं था कहाँ जाए रेफरल के लिए<br>• रेफरल स्थान थे दूर से निवास स्थान<br>• नहीं था के लिए पैसा परिवहन<br>• नहीं था पापा को पैसा के लिए अस्पताल का खर्च<br>• अन्य
अन्य कारण बताएं	
यदि किसी बीमारी के लिए देखभाल की मांग की गई थी, तो क्या<br>बच्चे का मूल्यांकन करने के लिए निम्नलिखित उपकरणों का उपयोग किया गया:	चेकबॉक्स, आवश्यक<br>• डिजिटल टाइमर<br>• डिजिटल थर्मामीटर<br>• MUAC टेप<br>• वजन तौलने का पैमाना<br>• पल्स ऑक्सीमीटर<br>• स्टेथोस्कोप<br>• अन्य

अन्य निर्दिष्ट करें	
यदि बच्चे की साँस तेज़ चल रही थी, तो क्या ऑक्सीजन दी जा रही थी?<br>संतृप्ति स्तर की जाँच की गई	हाँनहीं, आवश्यक<br>• हाँ<br>• नहीं
क्या ऑक्सीजन संतृप्ति स्तर कम था (SpO2 < 90%)	हाँनहीं, आवश्यक<br>• हाँ<br>• नहीं
आपका बच्चा कितनी बार स्वास्थ्य केंद्र गया है? किसी भी बीमारी के कारण बाह्य रोगी देखभाल की सुविधा<br>पिछले 1 महीने	
ड्रग्स	
जांच/परीक्षण	
परामर्श शुल्क	
स्वास्थ्य सुविधा तक और वहां से परिवहन	
अतिरिक्त भोजन लागत (बच्चे के लिए खरीदा गया कोई विशेष भोजन)<br>शिशु के लिए उदाहरण के लिए कोई भी दूध या अन्य भोजन, तरल पदार्थ और<br>साथ आए देखभाल करने वालों के लिए भोजन खरीदा गया<br>शिशु को उपचार प्रदाता को सौंपना)	
देखभालकर्ता के लिए अतिरिक्त व्यय	
मजदूरी का नुकसान	
यदि पारंपरिक चिकित्सक इस प्रकरण के लिए आए,<br>भुगतान की गई राशि (कुल व्यय)	
अन्य व्यय	
कुल व्यय	
बीमारी 1	
बीमारी 2	
बीमारी 3	
घरेलू उपचार पर खर्च की गई कुल राशि, यदि किसी भी बीमारी के लिए दिया गया	
पूरा?	• अपूर्ण<br>• असत्यापित<br>• पूर्ण
**Instrument: Hospitalization (hospitalization) साधन: अस्पताल में भर्ती (अस्पताल में भर्ती)**	

क्या बच्चा पिछले तीन महीनों में अस्पताल में भर्ती हुआ है?	हाँनहीं, आवश्यक<br>• हाँ<br>• नहीं
अस्पताल में भर्ती होने की संख्या	
नाम	
पिता का नाम	
अस्पताल में भर्ती होने के क्या कारण थे?	• रेडियो, आवश्यक<br>• सहज<br>• संकेत दिया गया
अस्पताल में भर्ती होने के कारण चुनें	• खाँसी<br>• साँस लेने में कठिनाई<br>• तेज़ साँस लेना<br>• न्यूमोनिया<br>• गंभीर छाती अंदर की ओर खींचना<br>• स्ट्रिडोर (सांस लेने में ध्वनि)<br>• घरघराहट<br>• दस्त<br>• निर्जलीकरण/पानी की कमी<br>• धंसी हुई आंखें<br>• बेचेन होना<br>• मल में रक्त<br>• बुखार 100.4? F<br>• गर्दन में अकड़न<br>• उल्टी करना<br>• स्तनपान/पानी पीने में असमर्थ होना<br>• आक्षेप<br>• सुस्त<br>• अचेत<br>• आँख से स्राव<br>• कान से स्राव<br>• त्वचा संक्रमण<br>• उभार<br>• ठंडा/चल रहा n<br>• बहुत कमज़ोर/कुपोषण<br>• खसरा<br>• मुंह में अल्सर<br>• नाभि लाल या जल निकासी<br>• त्वचा पर फुंसियां<br>• पीले तलवे<br>• कम शारीरिक तापमान/ हाइपोथर्मिया

अस्पताल का प्रकार	• सरकार<br>• प्राइवेट<br>• अन्य
क्या आपको एडमिशन की तारीख याद है?<br>मुक्ति की तारीख?	• हाँ<br>• नहीं
प्रवेश की तिथि क्या थी?	
डिस्चार्ज की तारीख क्या थी?	
यदि तारीखें, अस्पताल में भर्ती के दिनों की संख्या याद न आ सके	
यदि दस्तावेज उपलब्ध हों तो निदान की प्रतिलिपि प्रस्तुत करें	
प्रवेश शुल्क	
अस्पताल का बिस्तर	
ड्रग्स	
परामर्श शुल्क	
जांच/परीक्षण	
भोजन (शिशु के लिए खरीदा गया कोई विशेष भोजन, उदाहरण के लिए दूध या अन्य भोजन, तरल पदार्थ और अस्पताल में शिशु के साथ रहने वाले देखभालकर्ताओं के लिए खरीदा गया भोजन)	
अस्पताल तक और वहां से आने-जाने का परिवहन	
मजदूरी का नुकसान	
देखभालकर्ता के लिए अतिरिक्त व्यय	
अन्य	
कुल व्यय	
पूरा?	• अपूर्ण<br>• असत्यापित<br>• पूर्ण

Protect Prevent Treatment Questions

**Question (प्रश्न)**	**Outcome (परिणाम)**
Name of respondent<br>(उत्तरदाता का नाम)	
Respondent relation<br>(उत्तरदाता का संबंध)	1=Father (पिता)<br>2=Mother (माँ)<br>3=Grandmother (दादी)<br>4=Grandfather (दादा)
Name of child<br>(बच्चे का नाम)	
Name of father<br>(पिता का नाम)	
**Knowledge assessment for under-five pneumonia**<br>**(पाँच वर्ष से कम उम्र के बच्चों में निमोनिया की जानकारी का आकलन)**	
Do you know about under-five pneumonia?<br>(क्या आपको पाँच वर्ष से कम उम्र के बच्चों में निमोनिया के बारे में पता है?)	
If Yes, specify the symptoms of under-five pneumonia<br>(यदि हाँ, तो निमोनिया के लक्षण बताएं)	
Cough (Khasi)<br>(खाँसी)	1=Yes (हाँ)<br>0=No (नहीं)
Fast Breathing	1=Yes (हाँ)

(तेज सांस लेना)	0=No (नहीं)
Chest Indrawing (or pasliyon ka chalna/Chaati me ghadde padna) (पसलियों का चलना/छाती में गड्ढे पड़ना)	1=Yes (हाँ)<br>0=No (नहीं)
Stridor (khar khar or khad khad ki Awaaz)<br>(घरघराहट या खरखराहट की आवाज)	1=Yes (हाँ)<br>0=No (नहीं)
Difficulty in Breathing<br>(सांस लेने में कठिनाई)	1=Yes (हाँ)<br>0=No (नहीं)
Do you know danger signs of pneumonia?<br>(क्या आपको निमोनिया के खतरनाक लक्षणों के बारे में पता है?)	1=Yes (हाँ)<br>0=No (नहीं)
Tell us the danger signs<br>(खतरनाक लक्षण बताएं)	
Not able to drink or breastfeed<br>(दूध पीने या स्तनपान करने में असमर्थ)	1=Yes (हाँ)<br>0=No (नहीं)
Vomits everything<br>(हर चीज उगल देना)	1=Yes (हाँ)<br>0=No (नहीं)
Convulsions<br>(झटके आना)	1=Yes (हाँ)<br>0=No (नहीं)
Lethargic/Unconsciousness<br>(सुस्ती/बेहोशी)	1=Yes (हाँ)<br>0=No (नहीं)
Do you know where to take the child in case you identify any danger sign/symptom of pneumonia?<br>(यदि आपको निमोनिया के कोई खतरनाक लक्षण दिखते हैं, तो क्या आपको पता है कि बच्चे को कहाँ ले जाना चाहिए?)	1=Yes (हाँ)<br>0=No (नहीं)

Health and wellness centers (HWCs) / Sub-Centers / Govt. Dispensary<br>(स्वास्थ्य और कल्याण केंद्र (एचडब्ल्यूसी) / उप-केंद्र / सरकारी औषधालय)	1=Yes (हाँ)<br>0=No (नहीं)
Primary Health Centers (PHC)<br>(प्राथमिक स्वास्थ्य केंद्र (पीएचसी))	1=Yes (हाँ)<br>0=No (नहीं)
Community Health Centers (CHC)<br>(सामुदायिक स्वास्थ्य केंद्र (सीएचसी))	1=Yes (हाँ)<br>0=No (नहीं)
District Hospital (DH)<br>(जिला अस्पताल (डीएच))	1=Yes (हाँ)<br>0=No (नहीं)
Chemist<br>(केमिस्ट)	1=Yes (हाँ)<br>0=No (नहीं)
Local Private Doctor<br>(स्थानीय निजी डॉक्टर)	1=Yes (हाँ)<br>0=No (नहीं)
Registered Medical Practitioner<br>(पंजीकृत चिकित्सा व्यवसायी)	1=Yes (हाँ)<br>0=No (नहीं)
Quack<br>(झोलाछाप डॉक्टर)	1=Yes (हाँ)<br>0=No (नहीं)
Other (If any, please specify)<br>(अन्य (यदि कोई हो, कृपया बताएं))	
Did/Are you exclusively breastfeeding your child for first 6 months? (क्या आपने पहले 6 महीनों तक अपने बच्चे को विशेष रूप से स्तनपान कराया?)	1=Yes (हाँ)<br>0=No (नहीं)
If No, please specify, what did you give?<br>(यदि नहीं, तो कृपया बताएं, आपने क्या दिया?)	

Janam Ghutti (birth-tonic)<br>(जन्म घुट्टी)	1=Yes (हाँ)<br>0=No (नहीं)
Formula Milk<br>(फार्मूला दूध)	1=Yes (हाँ)<br>0=No (नहीं)
Water (पानी)	1=Yes (हाँ)<br>0=No (नहीं)
Cow/Buffalo Milk (गाय/भैंस का दूध)	1=Yes (हाँ)<br>0=No (नहीं)
Did your child get Vitamin A supplementation?<br>(क्या आपके बच्चे को विटामिन ए अनुपूरण मिला?)	1=Yes (हाँ)<br>0=No (नहीं)
Does the family have immunization card available at the time of visit? (क्या परिवार के पास यात्रा के समय टीकाकरण कार्ड उपलब्ध था?)	1=Yes (हाँ)<br>0=No (नहीं)
Pertussis<br>(काली खांसी)	1=Yes (हाँ)<br>0=No (नहीं)<br>Don't Know (नहीं पता)
Measles<br>(खसरा)	1=Yes (हाँ)<br>0=No (नहीं)<br>Don't Know (नहीं पता)
HIB<br>(एचआईबी)	1=Yes (हाँ)<br>0=No (नहीं)

	Don’t Know (नहीं पता)
Pneumococcal conjugated vaccine<br>(न्यूमोकोकल संयुग्मित टीका)	1=Yes (हाँ)<br>0=No (नहीं)<br>Don’t Know (नहीं पता)
Rotavirus (रोटावायरस)	1=Yes (हाँ)<br>0=No (नहीं)<br>Don’t Know (नहीं पता)
Do you wash your and your child's hand daily before or after meals or after coming from outside? (क्या आप और आपका बच्चा रोजाना भोजन से पहले या बाद में या बाहर से आने के बाद हाथ धोते हैं?)	1=Yes (हाँ)<br>0=No (नहीं)
Wash hands without soap<br>(बिना साबुन के हाथ धोएं)	1=Yes (हाँ)<br>0=No (नहीं)
Do you have any exposer to the pneumonia awareness activity before ongoing Social behavioral change communication meeting?<br>क्या आपको चल रही सामाजिक व्यवहार परिवर्तन संचार बैठक से पहले निमोनिया जागरूकता गतिविधि का कोई अनुभव है?	1=Yes (हाँ)<br>0=No (नहीं)
Exposer through<br>(एक्सपोज़र के माध्यम से)	1 = Community (समुदाय)<br>2 = Media (मीडिया)<br>3 = HWC (स्वास्थ्य और कल्याण केंद्र)<br>4 = Health Care Centre (स्वास्थ्य देखभाल केंद्र)<br>5 = Other (अन्य)

समुदाय	1 = Accredited Social Health Activist (प्रमाणित सामाजिक स्वास्थ्य कार्यकर्ता)
Media<br>(मिडिया)	1 = Radio (रेडियो)<br>2 = Pamphlet (पंपलेट)<br>3 = Film (फिल्म)<br>4 = Information education communication (IEC) material (सूचना, शिक्षा, संचार (आईईसी) सामग्री)<br>5 = TV Advertisements (टीवी विज्ञापन)<br>6 = Camps (शिविर)<br>7 = Internet (इंटरनेट)
Health and Wellness Centre<br>(स्वास्थ्य और कल्याण केंद्र)	1 = Community Health Officer (सामुदायिक स्वास्थ्य अधिकारी)<br>2 = Auxiliary Nurse Midwives (सहायक नर्स दाई)
Health care centers<br>(स्वास्थ्य देखभाल केंद्र)	1 = GH (सरकारी अस्पताल)<br>2 = CHC (सामुदायिक स्वास्थ्य केंद्र)<br>3 = PHC (प्राथमिक

	स्वास्थ्य केंद्र)<br>4 = Private facility<br>(निजी सुविधा)
Any other source of exposer, specify<br><br>(एक्सपोजर का कोई अन्य स्रोत, निर्दिष्ट करें)	
