# Supplementary material for: Determinants and Experiences of Care‐Seeking for Childhood Pneumonia in a Rural Indian Setting: A Mixed‐Methods Study
Source: Health Expect. 2025 Apr 16;28(2):e70263. doi: 10.1111/hex.70263 (PMC12002083; doi:10.1111/hex.70263)
Supplement: Supplementary file 5 — Annexure V IDI Guide qualitative. [file HEX-28-e70263-s006.pdf]

In-depth Interview Guide

Primary Caregivers/ Mother/Father/Family

1. Ice Breaking
    a. How are you today?
    b. How many children do you have? Boy… Girl… How many of them are of less than 5 years?
    c. How are your children today?

2. Knowledge
    a. How do you identify if a child ill?
    b. Which are the signs and symptoms which indicate that the child is suffering from pneumonia? Spontaneous / probe. How did you come to know about these?
    c. If a child experiences any symptoms of pneumonia, what should be done? Probe: treatment at home, treatment from health care provider, does it vary with symptoms and signs or age of the child
    d. Is there any difference in care seeking for a child if child is a boy of a girl? If yes, what is the difference and why?/ Why not?

3. Practice:
    a. What do you do if you observe any sign and symptoms of pneumonia in your child? Probe: symptoms: Cough & cold, fast breathing, chest indrawing, danger signs.
    b. Which is the most preferred source of care for pneumonia? Where do you take your child for treatment of respiratory symptoms/symptoms of pneumonia? Why
    c. What are the issues that you need to consider in order seek care. Probe: availability of health care provider, agreement of the decision maker in the family, transportation, service availability time of the facility/availability of the health care provider, cost of treatment, somebody to accompany.
    d. How easy or difficult is it for you to seek treatment of your child from the care provider of your choice?
    e. From whom do you take advice to decide on the source of care for your sick child?
    f. If the health care provider prescribes any medicines, from where do you get them?
    g. Do you face any challenge in complying with full treatment course or follow up care?
    h. Did ASHA visit your home to observe your child during sickness? What did the ASHA observe or do (measure temperature, count respiratory rate, examine the child) during her visit? What did ASHA advice?
