# Supplementary material for: Determinants and Experiences of Care‐Seeking for Childhood Pneumonia in a Rural Indian Setting: A Mixed‐Methods Study
Source: Health Expect. 2025 Apr 16;28(2):e70263. doi: 10.1111/hex.70263 (PMC12002083; doi:10.1111/hex.70263)
Supplement: Supplementary file 6 — Annexure VI translated qualitative. [file HEX-28-e70263-s007.pdf]

GUIDE FOR STAKEHOLDERS

(Healthcare staff and Local representative from the community)

□□□□

□□□□□□□□□ □□□□□□□□ □□ □□□□□□ □□ □□□□□□□ □□□□□□□□□)

Innovation Source:
What sources of information do you trust when it comes to learning about pneumonia prevention and treatment?

नवाचार स्रोत:
निमोनिया की रोकथाम और उपचार के बारे में सीखने के लिए आप किन जानकारी स्रोतों पर भरोसा करते हैं?

Innovation Evidence-Base:
What do you know about the effectiveness of different pneumonia prevention and treatment methods?

नवाचार प्रमाण-आधार:
आप विभिन्न निमोनिया रोकथाम और उपचार विधियों की प्रभावशीलता के बारे में क्या जानते हैं?

Innovation Relative Advantage:
How do you compare different pneumonia prevention and treatment methods in terms of their effectiveness, cost, and ease of use?

नवाचार तुलनात्मक लाभ:
आप विभिन्न निमोनिया रोकथाम और उपचार विधियों की प्रभावशीलता, लागत और उपयोग में सरलता के आधार पर उनकी तुलना कैसे करते हैं?

Innovation Adaptability:
How do you think pneumonia prevention and treatment methods can be adapted to fit the local context and needs of your community?

नवाचार अनुकूलता:
आपके अनुसार निमोनिया रोकथाम और उपचार विधियों को स्थानीय संदर्भ और आपकी समुदाय की आवश्यकताओं के अनुसार कैसे अनुकूलित किया जा सकता है?

Innovation Trialability:
Have you ever tried any new pneumonia prevention and treatment methods on a small scale? If so, what were the results?

नवाचार प्रयोज्यता:

क्या आपने कभी छोटे पैमाने पर कोई नई निमोनिया रोकथाम और उपचार विधियाँ आजमाई हैं? यदि हाँ, तो परिणाम क्या थे?

Innovation Complexity:
What do you think are the most challenging aspects of implementing pneumonia prevention and treatment methods in your community?

नवाचार जटिलता:
आपके अनुसार आपकी समुदाय में निमोनिया रोकथाम और उपचार विधियों को लागू करने में सबसे बड़ी चुनौतियाँ क्या हैं?

Who do people in the village trust to give them information about pneumonia prevention and treatment?

गांव के लोग निमोनिया की रोकथाम और उपचार के बारे में जानकारी देने के लिए किस पर भरोसा करते हैं?

What do people in the village know about the different ways to prevent and treat pneumonia?

गांव के लोग निमोनिया की रोकथाम और उपचार के विभिन्न तरीकों के बारे में क्या जानते हैं?

Which ways to prevent and treat pneumonia do people in the village think are the best, and why?

गांव के लोग निमोनिया की रोकथाम और उपचार के लिए कौन से तरीके सर्वोत्तम मानते हैं और क्यों?

How can the ways to prevent and treat pneumonia be changed to fit the needs of the village?

गांव की जरूरतों के अनुरूप निमोनिया की रोकथाम और उपचार के तरीकों को कैसे बदला जा सकता है?

Have people in the village ever tried new ways to prevent and treat pneumonia on a small scale? If so, what happened?

क्या गांव के लोगों ने कभी छोटे पैमाने पर निमोनिया की रोकथाम और उपचार के लिए नए तरीके आजमाए हैं? अगर हां, तो क्या हुआ?

What are the biggest challenges to implementing ways to prevent and treat pneumonia in the village?

गांव में निमोनिया की रोकथाम और उपचार के तरीकों को लागू करने में सबसे बड़ी चुनौतियां क्या हैं?

Inner Setting:
What are the existing relationships and networks within the village that can be leveraged to promote pneumonia prevention and treatment?

आंतरिक व्यवस्था:
गाँव में पहले से मौजूद कौन से संबंध और नेटवर्क निमोनिया रोकथाम और उपचार को बढ़ावा देने के लिए उपयोग किए जा सकते हैं?

Are there any formal or informal teams or groups within the village that are responsible for pneumonia prevention and treatment?

क्या गाँव में कोई औपचारिक या अनौपचारिक टीमें या समूह हैं जो निमोनिया रोकथाम और उपचार के लिए जिम्मेदार हैं?

How do people in the village communicate and share information about pneumonia prevention and treatment?

गांव के लोग निमोनिया की रोकथाम और उपचार के बारे में जानकारी कैसे साझा करते हैं?

Outer Setting:
Are there any government policies or programs that support pneumonia prevention and treatment in the village?

बाहरी व्यवस्था:
क्या गाँव में निमोनिया रोकथाम और उपचार का समर्थन करने वाली कोई सरकारी नीतियाँ या कार्यक्रम हैं?

Are there any external organizations or resources that can be leveraged to support pneumonia prevention and treatment in the village?

क्या कोई बाहरी संगठन या संसाधन हैं जिनका उपयोग गांव में निमोनिया की रोकथाम और उपचार में किया जा सकता है?

Characteristics of Individuals:
How does age affect people's knowledge and practices related to pneumonia prevention and treatment?

व्यक्तियों की विशेषताएँ:
उम्र का निमोनिया रोकथाम और उपचार से संबंधित ज्ञान और व्यवहारों पर क्या प्रभाव पड़ता है?

How does gender affect people's access to information and resources related to pneumonia prevention and treatment?

निमोनिया की रोकथाम और उपचार से संबंधित जानकारी और संसाधनों तक लोगों की पहुंच पर लिंग का क्या प्रभाव पड़ता है?

How does education level affect people's understanding of pneumonia prevention and treatment?

शिक्षा का स्तर निमोनिया की रोकथाम और उपचार के बारे में लोगों की समझ को कैसे प्रभावित करता है?

Process:
What are the specific steps involved in preventing and treating pneumonia in the village, and how are they currently being implemented?

प्रक्रिया:
गाँव में निमोनिया रोकथाम और उपचार की विशेष चरण कौन-कौन से हैं, और उन्हें वर्तमान में कैसे लागू किया जा रहा है?

Are there any gaps or challenges in the current process of pneumonia prevention and treatment in the village?

क्या गांव में निमोनिया की रोकथाम और उपचार की वर्तमान प्रक्रिया में कोई कमी या चुनौतियां हैं?

How can the process of pneumonia prevention and treatment be improved to better meet the needs of the village?

गांव की आवश्यकताओं को बेहतर ढंग से पूरा करने के लिए निमोनिया की रोकथाम और उपचार की प्रक्रिया को कैसे बेहतर बनाया जा सकता है?

Implementation:
Who is responsible for implementing and delivering pneumonia prevention and treatment methods in the village, and what are their roles and responsibilities?

कार्यान्वयन:
गाँव में निमोनिया रोकथाम और उपचार विधियों को लागू करने और वितरित करने की जिम्मेदारी किसकी है, और उनकी भूमिकाएँ और ज़िम्मेदारियाँ क्या हैं?

How are different stakeholders involved in the implementation and delivery of pneumonia prevention and treatment methods?

निमोनिया की रोकथाम और उपचार विधियों के कार्यान्वयन और वितरण में विभिन्न हितधारक किस प्रकार शामिल हैं?

Are there any challenges or barriers to implementing and delivering pneumonia prevention and treatment methods in the village?

क्या गांव में निमोनिया की रोकथाम और उपचार विधियों को लागू करने और वितरित करने में कोई चुनौतियां या बाधाएं हैं?

Innovation:
What specific pneumonia prevention and treatment methods are being used in the village, and how effective are they?

नवाचार:
गाँव में कौन सी विशेष निमोनिया रोकथाम और उपचार विधियाँ उपयोग की जा रही हैं, और वे कितनी प्रभावी हैं?

Are there any new or innovative pneumonia prevention and treatment methods that could be introduced in the village?

क्या गांव में निमोनिया की रोकथाम और उपचार के लिए कोई नई या अभिनव विधियां शुरू की जा सकती हैं?

How can existing pneumonia prevention and treatment methods be adapted to better fit the needs of the village?

निमोनिया की रोकथाम और उपचार की मौजूदा विधियों को गांव की आवश्यकताओं के अनुरूप कैसे अनुकूलित किया जा सकता है?

In-depth Interview Guide

गहन साक्षात्कार गाइड

Primary Caregivers/ Mother/Father/Family

प्राथमिक देखभालकर्ता/ माँ/पिता/परिवार

---

1. Ice Breaking (परिचयात्मक बातचीत)

a. How are you today?

• आज आप कैसे हैं?

b. How many children do you have? Boy… Girl… How many of them are of less than 5 years?

• आपके कितने बच्चे हैं? लड़का... लड़की... इनमें से कितने 5 वर्ष से कम उम्र के हैं?

c. How are your children today?

• आज आपके बच्चे कैसे हैं?

2. Knowledge (ज्ञान)

a. How do you identify if a child is ill?

• आप कैसे पहचानते हैं कि कोई बच्चा बीमार है?

b. Which are the signs and symptoms which indicate that the child is suffering from pneumonia? Spontaneous / probe. How did you come to know about these?

• कौन-कौन से लक्षण और संकेत होते हैं जो यह दर्शाते हैं कि बच्चा निमोनिया से पीड़ित है? यह जानकारी आपको कैसे मिली?

c. If a child experiences any symptoms of pneumonia, what should be done? Probe: treatment at home, treatment from health care provider, does it vary with symptoms and signs or age of the child

• यदि किसी बच्चे में निमोनिया के लक्षण दिखाई देते हैं, तो क्या किया जाना चाहिए? प्रश्न पूछने योग्य बिंदु: घरेलू उपचार, स्वास्थ्य सेवा प्रदाता से उपचार, क्या लक्षणों और बच्चे की उम्र के अनुसार उपचार भिन्न होता है?

d. Is there any difference in care-seeking for a child if the child is a boy or a girl? If yes, what is the difference and why? / Why not?

• क्या लड़का या लड़की होने पर बच्चे की देखभाल में कोई अंतर होता है? यदि हाँ, तो यह अंतर क्या है और क्यों? / यदि नहीं, तो क्यों नहीं?

3. Practice (अभ्यास)

a. What do you do if you observe any signs and symptoms of pneumonia in your child? Probe: symptoms: Cough & cold, fast breathing, chest indrawing, danger signs.

• यदि आपके बच्चे में निमोनिया के कोई लक्षण दिखाई दें, तो आप क्या करते हैं? प्रश्न पूछने योग्य बिंदु: लक्षण - खाँसी और जुकाम, तेज़ साँस चलना, छाती धँसना, गंभीर लक्षण।

b. Which is the preferred source of care for pneumonia? Where do you take your child for treatment of respiratory symptoms/symptoms of pneumonia? Why?

• निमोनिया के इलाज के लिए आप किस प्रकार की चिकित्सा सुविधा को प्राथमिकता देते हैं? आप अपने बच्चे को श्वसन संबंधी लक्षणों/निमोनिया के लक्षणों के लिए कहाँ ले जाते हैं? क्यों?

c. What are the issues that you need to consider in order to seek care? Probe: availability of health care provider, agreement of the decision maker in the family, transportation, service availability time of the facility/availability of the health care provider, cost of treatment, somebody to accompany.

• देखभाल प्राप्त करने के लिए किन मुद्दों पर विचार करने की आवश्यकता होती है? प्रश्न पूछने योग्य बिंदु: स्वास्थ्य सेवा प्रदाता की उपलब्धता, परिवार में निर्णय लेने वाले की सहमति, परिवहन, चिकित्सा सुविधा का समय, स्वास्थ्य सेवा प्रदाता की उपलब्धता, इलाज की लागत, किसी का साथ जाना।

d. How easy or difficult is it for you to seek treatment of your child from the care provider of your choice?

• अपनी पसंद के स्वास्थ्य सेवा प्रदाता से अपने बच्चे के लिए उपचार प्राप्त करना आपके लिए कितना आसान या कठिन है?

e.From whom do you take advice to decide on the source of care for your sick child?

• अपने बीमार बच्चे के इलाज के लिए चिकित्सा सुविधा चुनने में आप किससे सलाह लेते हैं?

f. If the health care provider prescribes any medicines, from where do you get them?

• यदि स्वास्थ्य सेवा प्रदाता कोई दवा लिखते हैं, तो आप उन्हें कहाँ से प्राप्त करते हैं?

g. Do you face any challenge in complying with full treatment course or follow-up care?

• क्या आपको पूरे इलाज को पूरा करने या फॉलो-अप देखभाल में किसी प्रकार की कठिनाई का सामना करना पड़ता है?

क्या आपको पूरे इलाज को पूरा करने या बार-बार दिखाने जाने में कई भी प्रकार की कठिनाई का सामना करना पड़ा हो?

h. Did ASHA visit your home to observe your child during sickness? What did the ASHA observe or do (measure temperature, count respiratory rate, examine the child) during her visit? What did ASHA advice?

• क्या आशा कार्यकर्ता आपके घर पर आपके बच्चे की बीमारी के दौरान उसे देखने आई थी?

प्रश्न पूछने योग्य बिंदु: आशा कार्यकर्ता ने क्या देखा या किया (जैसे - तापमान मापा, साँस लेने की दर गिनी, बच्चे की जाँच की)? आशा कार्यकर्ता ने क्या सलाह दी?
