# Supplementary material for: Determinants and Experiences of Care‐Seeking for Childhood Pneumonia in a Rural Indian Setting: A Mixed‐Methods Study
Source: Health Expect. 2025 Apr 16;28(2):e70263. doi: 10.1111/hex.70263 (PMC12002083; doi:10.1111/hex.70263)
Supplement: Supplementary file 7 — Annexure VII Stakeholders IDI. [file HEX-28-e70263-s001.pdf]

GUIDE FOR STAKEHOLDERS

(Healthcare staff and Local representative from the community)

Innovation Source:

What sources of information do you trust when it comes to learning about pneumonia prevention and treatment?

Innovation Evidence-Base:

What do you know about the effectiveness of different pneumonia prevention and treatment methods?

Innovation Relative Advantage: How do you compare different pneumonia prevention and treatment methods in terms of their effectiveness, cost, and ease of use?

Innovation Adaptability: How do you think pneumonia prevention and treatment methods can be adapted to fit the local context and needs of your community?

Innovation Trialability: Have you ever tried any new pneumonia prevention and treatment methods on a small scale? If so, what were the results?

Innovation Complexity: What do you think are the most challenging aspects of implementing pneumonia prevention and treatment methods in your community?

Who do people in the village trust to give them information about pneumonia prevention and treatment?

What do people in the village know about the different ways to prevent and treat pneumonia?

Which ways to prevent and treat pneumonia do people in the village think are the best, and why?

How can the ways to prevent and treat pneumonia be changed to fit the needs of the village?

Have people in the village ever tried new ways to prevent and treat pneumonia on a small scale? If so, what happened?

What are the biggest challenges to implementing ways to prevent and treat pneumonia in the village?

Inner Setting:

- What are the existing relationships and networks within the village that can be leveraged to promote pneumonia prevention and treatment?

- Are there any formal or informal teams or groups within the village that are responsible for pneumonia prevention and treatment?

- How do people in the village communicate and share information about pneumonia prevention and treatment?

2. Outer Setting:

- Are there any government policies or programs that support pneumonia prevention and treatment in the village?
- Are there any cultural beliefs or practices that affect pneumonia prevention and treatment in the village?
- Are there any external organizations or resources that can be leveraged to support pneumonia prevention and treatment in the village?

3. Characteristics of Individuals:

- How does age affect people's knowledge and practices related to pneumonia prevention and treatment?
- How does gender affect people's access to information and resources related to pneumonia prevention and treatment?
- How does education level affect people's understanding of pneumonia prevention and treatment?

4. Process:

- What are the specific steps involved in preventing and treating pneumonia in the village, and how are they currently being implemented?
- Are there any gaps or challenges in the current process of pneumonia prevention and treatment in the village?
- How can the process of pneumonia prevention and treatment be improved to better meet the needs of the village?

5. Implementation:

- Who is responsible for implementing and delivering pneumonia prevention and treatment methods in the village, and what are their roles and responsibilities?
- How are different stakeholders involved in the implementation and delivery of pneumonia prevention and treatment methods?
- Are there any challenges or barriers to implementing and delivering pneumonia prevention and treatment methods in the village?

6. Innovation:

- What specific pneumonia prevention and treatment methods are being used in the village, and how effective are they?
- Are there any new or innovative pneumonia prevention and treatment methods that could be introduced in the village?
- How can existing pneumonia prevention and treatment methods be adapted to better fit the needs of the village?
