# Supplementary material for: Determinants and Experiences of Care‐Seeking for Childhood Pneumonia in a Rural Indian Setting: A Mixed‐Methods Study
Source: Health Expect. 2025 Apr 16;28(2):e70263. doi: 10.1111/hex.70263 (PMC12002083; doi:10.1111/hex.70263)

**SUPPLEMENTARY DOCUMENT**

Title: **Determinants of Care-Seeking for Childhood Pneumonia in a North Indian Rural setting: A Mixed-Methods Study**

**Content**

Box 1: Operational definitions used in the analyses

Graph 1: Directed Acyclic Graphs (DAG) Model-I between wealth index and care-seeking from appropriate sources

Graph 2: DAG Model of relationship between maternal education and care-seeking from appropriate sources

Graph 3: DAG Model of relationship between maternal age and care-seeking from appropriate sources

Table 1**:** Health Insurance Coverage as per the socioeconomic tertiles

Table 2**:** Sources of Care sought for under-five Children with Suspected Pneumonia as per the Health Insurance status

Graph 4: Distribution of care seeking for under-five children with suspected pneumonia by socio-economic tertiles. (N=231)

**Supplementary Box 1:** Operational definitions used in the analyses

**Potential pneumonia -** caregivers reported at least one of the following signs and symptoms:

- *For children aged 0-59 days:* fast breathing, difficulty in breathing, hypothermia, hyperthermia, inability to feed, convulsions, lethargy, unconsciousness, chest indrawing, stridor, or any danger sign.
- *For children aged 2 to 59 months:* cough, fast breathing, difficulty in breathing, chest indrawing, stridor, or any danger sign.

**Danger signs** for children aged 2-59 months: vomiting everything, not being able to breastfeed/ drink, convulsion, lethargy, or reduced level of consciousness.

**Delay in appropriate care-seeking**: seeking healthcare at a hospital or health center more than 24 hours after the caregiver recognised the signs or symptoms of pneumonia and/or any danger signs (cough and/or breathing difficulties).

**Caregiver:** someone who attends to the needs of a child and takes responsibility for its well-being.

**Supplementary Graph 1**: Directed Acyclic Graphs (DAG) Model-I between wealth index and care-seeking from appropriate sources.


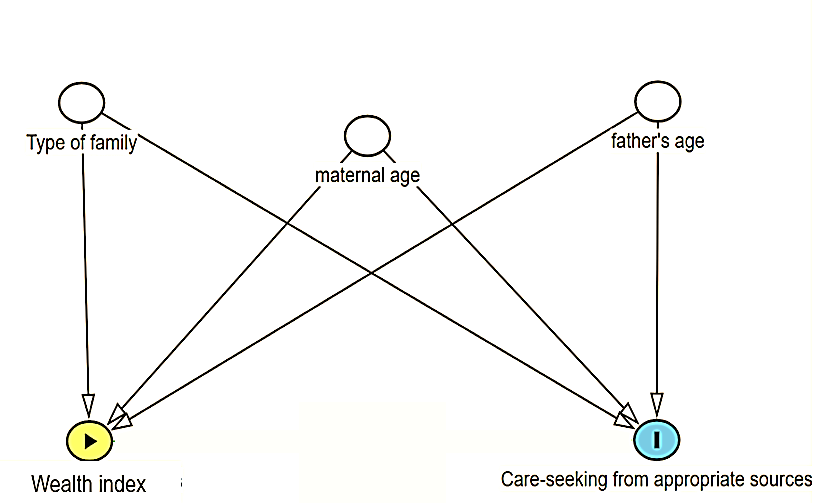


**Supplementary Graph 2:** DAG Model of relationship between maternal education and care-seeking from appropriate sources

**
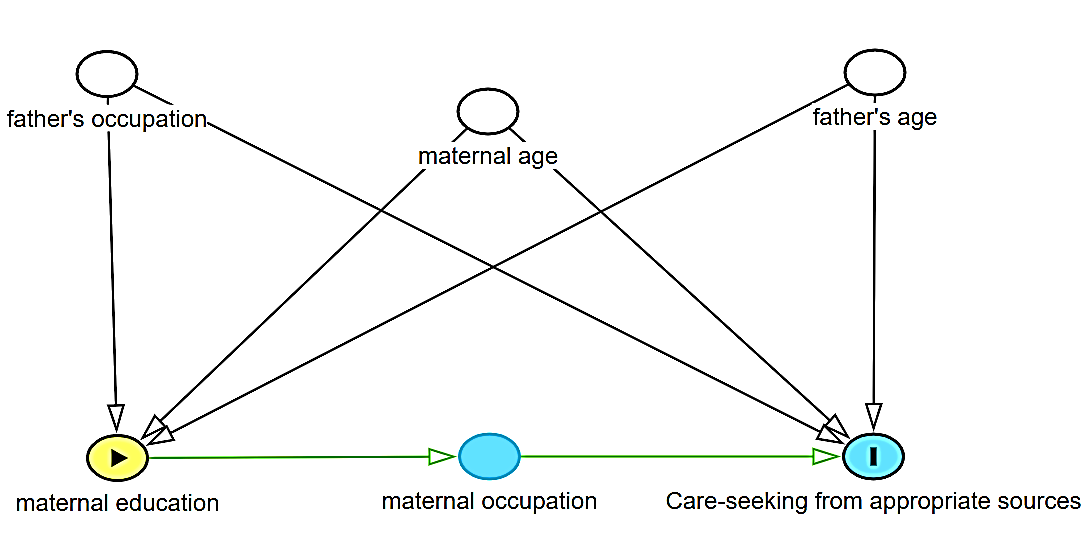
**

**Supplementary Graph 3**: DAG Model of relationship between maternal age and care-seeking from appropriate sources

**
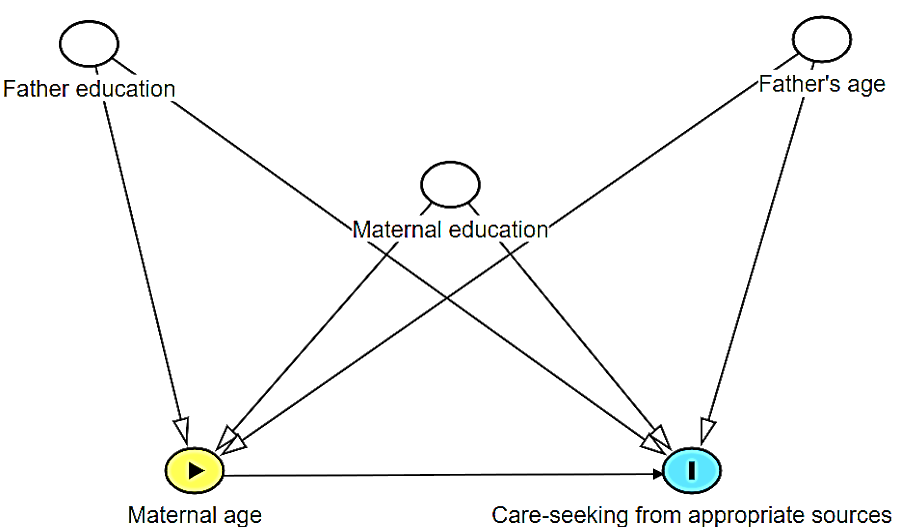
**

**Supplementary Table 1: Health Insurance Coverage as per the socioeconomic tertiles**

| **Health Insurance** | **Wealth Tertile** | | |
| --- | --- | --- | --- |
|  | **Poorest** | **Poor** | **Least poor** |
| Yes | 17 /76 (22.4%) | 31/76 (40.8%) | 32/79 (40.5%) |
| No | 59/76 (77.6%) | 45/76(59.2%) | 47/79 (59.5%) |

**Supplementary Table 2: Sources of Care sought for under-five Children with Suspected Pneumonia as per the Health Insurance status**

| **Sources of Care** | **Health Insurance** | | **Total** |
| --- | --- | --- | --- |
|  | **Yes** | **No** |  |
| Government | 2 (2.5%) | 6 (4.0%) | 8 (3.5%) |
| Private | 25 (31.3%) | 32 (31.3%) | 57 (24.7%) |
| Non-RMP | 50 (62.5%) | 109 (72.2%) | 159 (68.8%) |
| No Care | 3 (3.8%) | 4 (2.6%) | 7 (3.0%) |
| **Total** | 80 (100%) | 151 (100%) | 231 (100%) |

**Supplementary Graph 4**: Distribution of care seeking for under-five children with suspected pneumonia by socio-economic tertiles. (N=231)


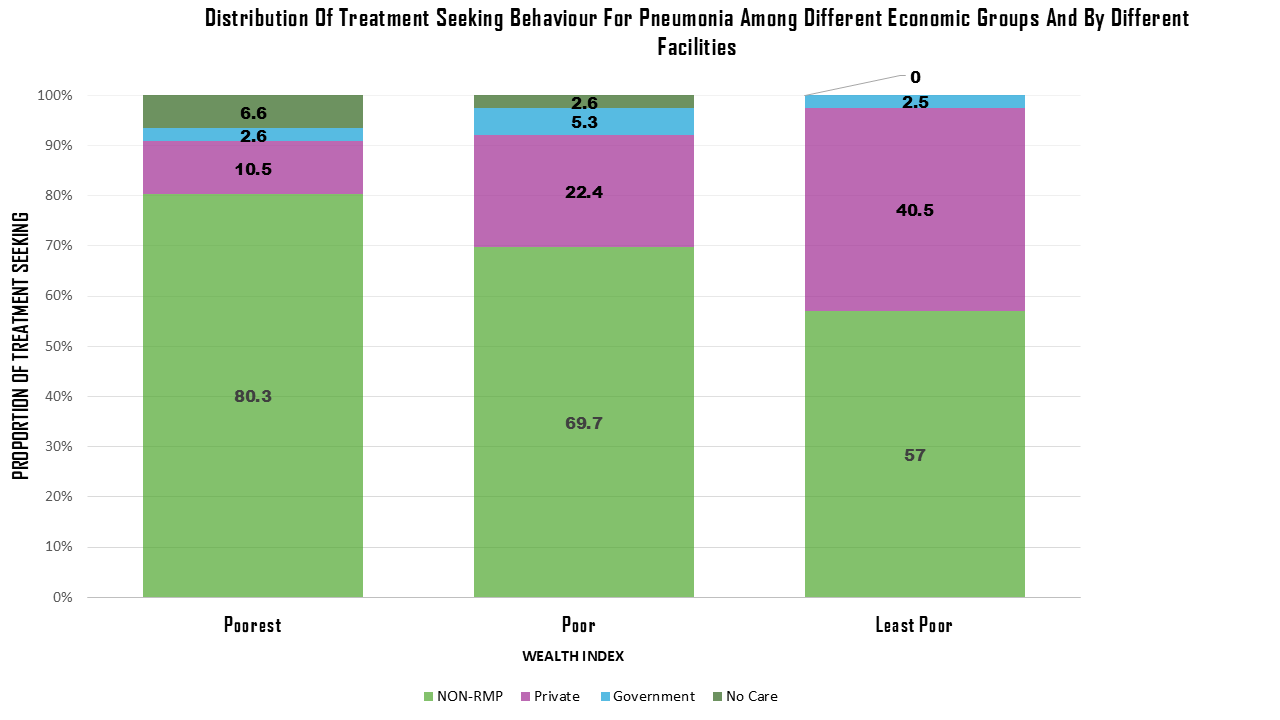

Supplement: Supplementary file 8 — SUPPLEMENTARY DOCUMENT healthcareseeking. [file HEX-28-e70263-s005.docx]
